# Identification of Gene Expression Signature Modulated by Nicotinamide in a Mouse Bladder Cancer Model

**DOI:** 10.1371/journal.pone.0026131

**Published:** 2011-10-10

**Authors:** Seon-Kyu Kim, Seok-Joong Yun, Jiyeon Kim, Ok-Jun Lee, Suk-Chul Bae, Wun-Jae Kim

**Affiliations:** 1 Department of Urology, Chungbuk National University College of Medicine, Cheongju, Korea; 2 BK21 Chungbuk Biomedical Science Center, Chungbuk National University School of Medicine, Cheongju, Korea; 3 Department of Pharmacology and Cancer Biology, Duke University Medical School, Durham, North Carolina, United States of America; 4 Department of Pathology, Chungbuk National University College of Medicine, Cheongju, Korea; 5 Department of Biochemistry, Chungbuk National University College of Medicine, Cheongju, Korea; University of Chicago, United States of America

## Abstract

**Background:**

Urinary bladder cancer is often a result of exposure to chemical carcinogens such as cigarette smoking. Because of histological similarity, chemically-induced rodent cancer model was largely used for human bladder cancer studies. Previous investigations have suggested that nicotinamide, water-soluble vitamin B3, may play a key role in cancer prevention through its activities in cellular repair. However, to date, evidence towards identifying the genetic alterations of nicotinamide in cancer prevention has not been provided. Here, we search for the molecular signatures of cancer prevention by nicotinamide using a N-butyl-N-(4-hydroxybutyl)-nitrosamine (BBN)-induced urinary bladder cancer model in mice.

**Methodology/Principal Findings:**

Via microarray gene expression profiling of 20 mice and 233 human bladder samples, we performed various statistical analyses and immunohistochemical staining for validation. The expression patterns of 893 genes associated with nicotinamide activity in cancer prevention were identified by microarray data analysis. Gene network analyses of these 893 genes revealed that the *Myc* and its associated genes may be the most important regulator of bladder cancer prevention, and the gene expression signature correlated well with protein expression data. Comparison of gene expression between human and mouse revealed that BBN-induced mouse bladder cancers exhibited gene expression profiles that were more similar to those of invasive human bladder cancers than to those of non-invasive human bladder cancers.

**Conclusions/Significance:**

This study demonstrates that nicotinamide plays an important role as a chemo-preventive and therapeutic agent in bladder cancer through the regulation of the *Myc* oncogenic signature. Nicotinamide may represent a promising therapeutic modality in patients with muscle-invasive bladder cancer.

## Introduction

Many bladder cancers can be linked to chemical carcinogens, and in western populations approximately one-third to one-half of bladder cancers are associated with cigarette smoking [1], [2]. Although the causative substance in cigarette smoke has not yet been identified, α- and β-naphthylamine are suspected agents. As putative carcinogens for bladder cancer, aromatic amines such as β-naphthylamine, 4-aminobiphenyl, benzidine, and 2-amino-1-naphthol may account for up to 20 to 30% of bladder cancer cases [3], [4]. There are two primary chemically-induced models of urinary bladder cancer in rodents, which are induced by administration of N-butyl-N-(4-hydroxybutyl)-nitrosamine (BBN) in either mice or rats [5], [6]. The resultant tumors are histologically similar to the human disease, and are manifested via two different carcinogenic pathways; namely, non-muscle-invasive bladder cancer (NMIBC) in rats and muscle-invasive bladder cancer (MIBC) in mice [7], [8]. Because human bladder cancer is often a result of exposure to chemical carcinogens, these rodent models are useful for understanding human bladder cancer. Williams et al illustrated the overall gene expression profiles of rodent tumors and provided a list of genes that are likely candidates for driving bladder cancer development and progression [9].

Nicotinic acid is a water-soluble vitamin belonging to the vitamin B family. It is found in many animal and plant tissues, and has antihyperlipidemic activity. In the body, nicotinic acid is converted to its active form nicotinamide, which is a component of the coenzymes nicotinamide adenine dinucleotide (NAD) and its phosphate form, NADP. These coenzymes play an important role in tissue respiration and in glycogen, lipid, amino acid, protein, and purine metabolism. Current research suggests that nicotinamide, or vitamin B3, may play a key role in cancer prevention through its activities in cellular repair. Leading scientists studying nicotinamide nutrition believe the vitamin shows promise for treating and even preventing cancer. Myron et al demonstrated that cells depleted of nicotinamide develop cancer ten times faster than those receiving sufficient amounts of the vitamin [10]. Diet is a major factor in the incidence of bladder cancer, with both beneficial and detrimental components, and nicotinamide is one of the beneficial components. Though the optimal dose of nicotinamide is still undetermined, nicotinamide is also a promising adjunct to chemotherapy because of its relationship to tumor necrosis (killing) factor, a relatively novel factor that selectively kills cancer cells [11], [12], [13]. In the current study, we sought to identify the genetic signatures of nicotinamide in cancer prevention using a BBN-induced mouse bladder cancer model, and investigated the potential efficacy of nicotinamide treatment in bladder cancer patients.

## Materials and Methods

### Mouse tissue samples

Animal studies were done in accordance with the guidelines of the Institutional Animal Care Committee of Chungbuk National University (permit numbers: CBNUA-030-0901-02). Female C3H/He mice were obtained from Japan SLC, Inc. (Shizuoka, Japan) at 6 weeks of age and were housed in cages (five per cage). The animals were kept in a lighted room for 12 hours each day, maintained at 23±2°C and 55%±5% humidity. BBN was obtained from Tokyo Kasei Kogyo Co. (Tokyo, Japan), and nicotinamide was purchased from Sigma Chemical Co. (St. Louis, MO). BBN was diluted with tap water to a final concentration of 0.05%. For analysis of the preventive effects of nicotinamide, drinking water was supplemented with 0.1%, 0.25%, 0.5%, or 1% nicotinamide with or without 0.05% BBN for 20 weeks. All mice received the first treatment at 7 weeks of age, and were sacrificed at 20 weeks after the first treatment. At necropsy, the urinary bladders of mice were inflated with normal saline and then removed, and under a high-intensity light were examined for gross lesions. After inspection, all bladders were frozen for histopathologic examination and subsequent molecular assays. A portion of each tissue was fixed and processed for routine paraffin embedding, cut into 5-µm sections, and mounted for hematoxylin and eosin staining for histopathology. Bladder tumors were classified into NMIBC and MIBC based on the extent of invasion into the muscle.

### RNA extraction, microarray experiments, and data processing

Total RNA was isolated using TRIzol reagent (Life Technologies, NY), according to the manufacturer's protocol. The quality and integrity of the RNA were confirmed by agarose gel electrophoresis and ethidium bromide staining, followed by visual examination under ultraviolet light. Five-hundred nanograms of total RNA were used for labeling and hybridization, according to the manufacturer's protocols (Illumina Mouse-6 BeadChips, version 1.0). Arrays were scanned with an Illumina Bead Array Reader confocal scanner (BeadStation 500GXDW; Illumina, Inc., San Diego, CA) according to the manufacturer's instructions. After scanning, the microarray data were filtered by detection *P*-value (*P*<0.05) which was provided by Genome Studio ^TM^ software (Illumina, Inc., San Diego, CA) and were normalized using quantile normalization in the R language environment (version 2.10.0, available at http://www.r-project.org/). Measured gene expression values were log2 transformed and median-centered across genes and samples. The full microarray data set is available in the National Center for Biotechnology Information (NCBI) Gene Expression Omnibus (GEO) public database under the data series accession number GSE21636. All microarray data is Minimum Information About a Microarray Experiment (MIAME) compliant.

### Human bladder cancer microarrays

For comparative analysis of mouse and human cancer, we used a previously published data set of human bladder cancer microarrays (Korean cohort), GSE13507, from the NCBI GEO public database [14]. The data set consisted of 165 primary bladder cancer samples (103 NMIBCs and 62 MIBCs), 58 samples of histologically normal-looking surrounding tissues, and 10 normal bladder mucosae from patients with benign diseases. These gene expression data were constructed on Illumina HumanWG-6 BeadChips (version 2). For further validation, we also used another human bladder cancer dataset (Spanish cohort, Affymetrix U133A GeneChip), which consisted of 24 NMIBCs, 66 MIBCs, and 38 normal bladder mucosae [15].

### Real time reverse transcriptase polymerase chain reaction (RT-PCR)analysis

RT-PCR was performed using a Rotor Gene 6000 PCR system (Corbett Research, Mortlake, Australia). RT-PCR reactions in micro-reaction tubes (Corbett Research) contained primers and SYBR Premix EX Taq (Takara Bio Inc., Otsu, Japan). Spectral data were captured and analyzed using Rotor-Gene Real-Time Analysis Software 6.0 Build 14 (Corbett Research). Gene expression was normalized to *Gapdh* expression.

### Immunohistochemical analysis

Immunohistochemical staining was performed on formalin-fixed, paraffin-embedded specimens of mouse normal and tumor bladders. Sections were incubated with the primary antibodies anti-Myc (Santa Cruz Biotechnology Inc., Santa Cruz, CA) and anti-Pmp22 (Abcam, UK). For automated staining, Benchmark XT autostainer (Roche Diagnostics, Indianapolis, IN) was used. After staining, we evaluated both the staining intensity and proportion of stained epithelial cells. Staining intensity was classified as follows: 1, weak; 2, moderate; and 3, strong. The proportion of positive cells was expressed as a percentage of the total number of epithelial cells examined, and assigned to one of five categories: 0, <5%; 1, 5–25%; 2, 26–50%; 3, 51–75%; and 4, >75%. Scores for the percentage of positive cells and staining intensity were multiplied together to produce the immunoreactivity score (IS) for each specimen. The IS of Myc and Pmp22 was measured in the nucleus and cytoplasm, respectively. Each specimen was examined and scored separately by three investigators, and discrepant scores were discussed until agreement was reached.

### Western blot analysis

After rinsing in distilled water, samples in glass tube containing 1 ml of cell lysis buffer were mechanically homogenized with a tissue homogenizer. The homogenate was transferred to a 1.5 ml tube and were centrifuged at 14,000 rpm for 5 minutes at 4°C. The upper aqueous phase was discarded and mixed with 2ul of phenylmethylsulfonyl fluoride and 200 ul of protein analysis buffer. After incubation for 1 hour at 4°C, samples were centrifuged at 14,000 rpm for 30 minutes at 4°C. The upper aqueous phase was transferred to new tube and stored at −80°C until use. Protein concentrations were determined by Bio-Rad protein assay (Bio-Rad Laboratories, Hercules, CA). Each sample (70 ug) was loaded on 10% (weight per volume) sodium dodecyl sulfate gel and transferred to polyvinylidene fluoride membrane (GE Healthcare, San Antonio, TX). Western blots were analyzed with antibodies against Pmp22 (abcam, Cambridge, UK) and Myc (LifeSpan BioSciences, Seattle, WA).

### Statistical analysis

To compare the molecular characteristics between different mouse groups, we performed a hierarchical clustering analysis. A hierarchical clustering algorithm, using the centered correlation coefficient as the measure of similarity and average linkage clustering, was applied as described in Eisen et al [16]. We identified genes that were differentially expressed between two groups using a random-variance two-sample t-test [17]. Genes were considered to have statistically significant differences in expression if the P-value was less than 0.001. We also performed a global test of whether the expression profiles differed between the classes by performing permutations. For each permutation, the *P*-values were re-computed and the number of genes significant at the 0.001 level was noted. The proportion of the permutations that resulted in at least as many significant genes as the actual data was taken as the significance level of the global test. While obtaining differentially expression genes, we selected genes that had a 1.2 fold or more difference of mean expression values of two groups.

To explore the relationships between tumorigenic genes that responded to nicotinamide, we examined functional associations among the genes and generated gene networks based on whether they had more interconnected genes than would be expected to occur by chance. The significance of each network was estimated using the scoring system provided by the Ingenuity Pathway Analysis Tool (version 7.5). The scores were determined by the number of differentially expressed genes within each of the networks and the strength of the associations among the network members.

As a means of comparing gene expression patterns between human and mouse samples, the microarray data of mouse models were pooled with the data set of human bladder cancers using HomoloGene (NCBI), a system for detection of the homologues genes in several eukaryotic genomes [18]. Before integration of the two data sets, the expression levels of each gene in each data set were standardized independently, to a mean of zero and a standard deviation of 1. To predict mouse models against human tissues, we applied four different prediction models: compound covariate predictor [19], linear discriminator analysis [20], nearest centroid classification [20], and support vector machines [21]. The models incorporated genes that were differentially expressed between the two classes using a two-sample t-test. Genes were considered to have statistically significant differences in expression if the *P*-value was less than 0.001. We estimated the prediction error of each model using leave-one-out cross-validation (LOOCV), as described by Simon et al [22]. For each LOOCV training set, the entire model-building procedure was repeated, including the gene selection process. Cross-species prediction procedure was performed in BRB ArrayTools (version 3.8.0).

## Results

### The efficacy of nicotinamide as a protective agent against BBN-induced mouse bladder cancers

**Figure 1:**
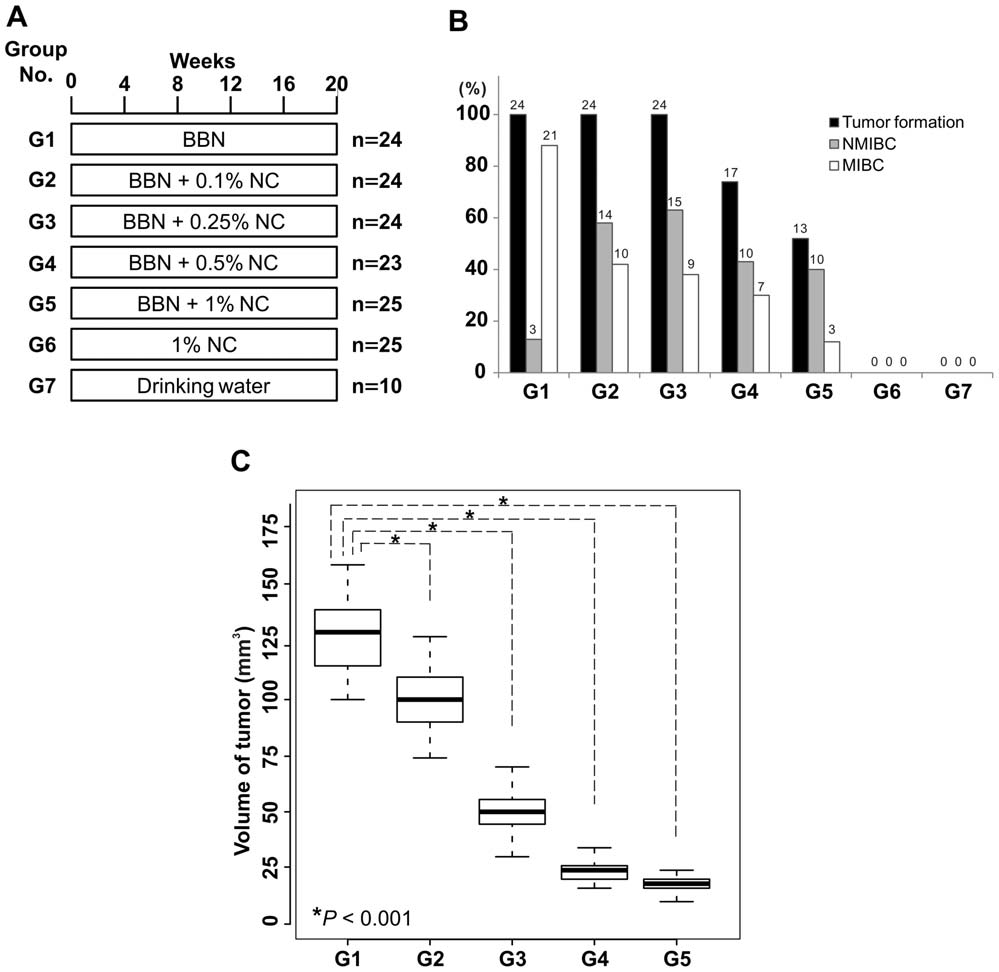
Efficacy of nicotinamide (NC) against N-butyl-N-(4-hydroxybutyl)-nitrosamine (BBN)-induced tumors. (A) Experimental protocol for analysis of the preventive effects of NC. Mice were treated with BBN alone or in combination with the indicated amounts of NC for 20 weeks. “n” indicates the number of mice in each group. A dose of 0.1% NC in drinking water is roughly equivalent to 100 mg/Kg/day. (B) Summary of the incidence of tumor formation, non-muscle invasive bladder cancer (NMIBC), and muscle invasive bladder cancer (MIBC) for each treatment group. The number above each bar indicates the number of mice in each histological category. (C) The box plot comparing tumor volumes of the treatment groups. The *P*-values were obtained by two sample t-tests between group G1 and other groups.

To investigate the effect of nicotinamide on tumor formation in the BBN-induced bladder cancer model, mice received 0.05% BBN alone or BBN plus increasing concentrations of nicotinamide in their drinking water for 20 weeks (Figure 1A). Noticeably, nicotinamide supplementation inhibited tumor formation. Supplementation with 0.5% nicotinamide reduced the tumor formation ratio from 100% to 74%, and 1% nicotinamide reduced the ratio even further, to 52% (Figure 1B). Histological analysis of bladder tumors from the various treatment groups revealed that the development of MIBC was markedly inhibited by nicotinamide. Treatment with 0.1% nicotinamide reduced the incidence of MIBC from 88 to 42%, and 1% nicotinamide reduced it even further, to 12% (Figure 1B), which indicated that nicotinamide strongly inhibits bladder tumor progression. Tumor volume was also remarkably reduced by nicotinamide. Most of the BBN-treated mice developed large tumors (average size, 129 mm^3^). Increasing the treatment of nicotinamide significantly reduced the average tumor size (99 to 18 mm^3^ when 0.1 to 1% nicotinamide were treated, Figure 1C). These results demonstrated that nicotinamide effectively prevents tumor formation and suppresses tumor growth and progression to MIBC.

### Differential gene expression patterns among experimental groups

To characterize the gene expression patterns of nicotinamide in cancer prevention, we randomly select 20 mouse bladders and performed a gene expression profiling; five BBN-induced mouse bladder tumors (MIBCs from Group G1, Figure 1A), five non-tumor-bearing bladders treated with BBN and nicotinamide (normal samples from Group G5, Figure 1A), five normal bladders treated with 1% nicotinamide (Group G6 in Figure 1A), and five normal bladders without treatment (Group G7 in Figure 1A). The 5 samples in each group were histologically identical each other. We first applied hierarchical clustering analysis of gene expression patterns to assess the molecular characteristics of the different experimental groups. As expected, hierarchical clustering analysis of gene expression data from all tissues yielded three major clusters, one representing the normal bladder group, a second representing the BBN-induced mouse bladder tumor group, and a third representing the BBN + nicotinamide-treated non-tumorigenic bladder group (Figure S1). Thus, gene expression patterns reflecting the molecular configuration are readily distinguishable between bladder tumors and non-tumor tissues.

**Figure 2:**
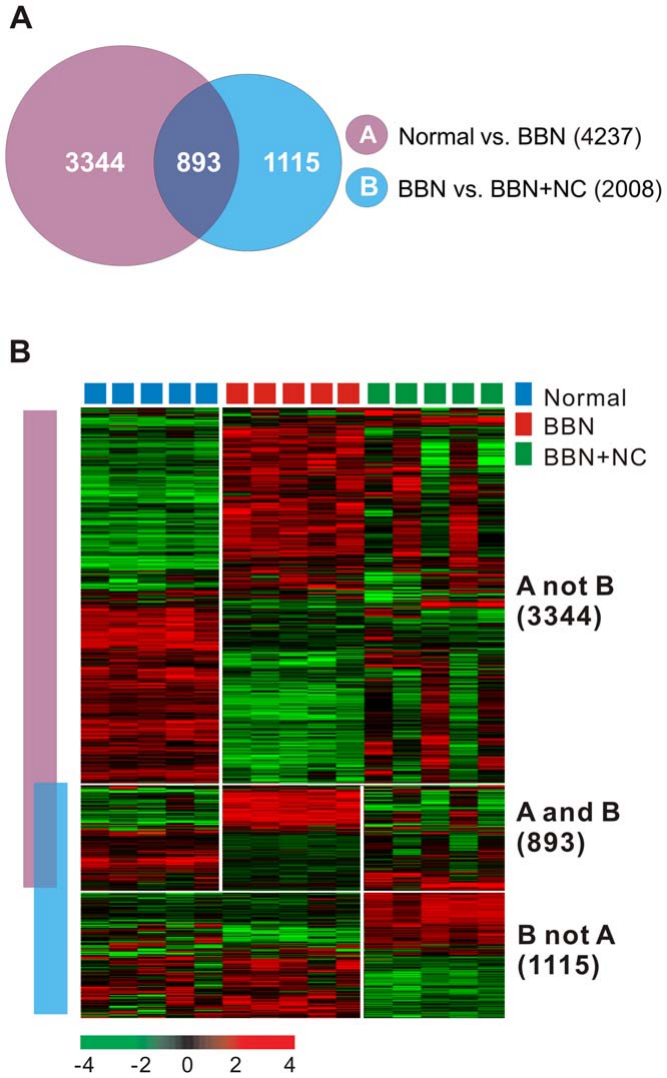
Comparative analysis of differentially expressed genes among the experimental groups. (A) Venn diagram of genes selected by two-sample t-test in the mouse model. The purple circle (gene list A) represents genes differentially expressed between normal bladder tissues and N-butyl-N-(4-hydroxybutyl)-nitrosamine (BBN)-induced bladder tumors. The blue circle (gene list B) represents genes differentially expressed between BBN-induced bladder tumors and BBN + nicotinamide (NC) treated non-tumor tissues. We applied a cut-off *P*-value of less than 0.001 to select genes whose expression was significantly different between the two groups. (B) Expression patterns of selected genes in the Venn diagram. Among the genes in A not B category, 1,525 genes (45.6%) showed up-down patterns and 1,819 genes (54.4%) showed down-up patterns in normal and BBN-induced cancer. In the A and B category, 290 genes (32.5%) and 603 genes (67.5%) represented down-up-down patterns and up-down-up patterns, respectively, in normal, BBN-induced cancer, and BBN + NC-treated bladder groups. In the B not A category, 606 genes (54.3%) displayed down-up patterns and 509 genes (45.7%) exhibited up-down patterns in BBN-induced cancer and BBN + NC-treated bladder groups. The data are presented in matrix format in which rows represent individual genes and columns represent each tissue. The red and green colors reflect high and low expression levels, respectively.

We next aimed to identify gene sets that were differentially expressed among the three different groups. Before analysis, normal bladders treated with nicotinamide were excluded, since there were no histopathologic or molecular differences between normal and nicotinamide-treated bladder (Figure S1). We applied Venn diagram comparison of two gene lists to select gene expression patterns common to carcinogenesis and cancer prevention. First, we generated two different gene lists by applying the two-sample t-test (Figure 2A, *P*<0.001). Gene list A represents the genes that were differentially expressed between the normal bladder and BBN-induced tumor groups, and gene list B represents the genes that were differentially expressed between the BBN- and BBN + nicotinamide-treated groups. When comparing the two gene lists, three different patterns were observed: A not B (3,344 genes), A and B (893 genes), and B not A (1,115 genes) (Figure 2B). Genes in the A not B category showed BBN-induced tumor-specific expression patterns, while genes in the B not A category displayed the expression patterns of genes that responded to nicotinamide. Genes in the A and B category exhibited both BBN-induced tumorigenic as well as nicotinamide-responsive expression patterns. These results signified that 893 genes in the A and B category were common to both carcinogenesis induced by BBN and to cancer prevention induced by nicotinamide.

### Biological insight to the gene expression patterns for cancer prevention

To identify the predominant signaling networks active in the prevention of bladder cancer by nicotinamide, gene network analysis of the 893 genes in the cancer prevention signature (Figure 2) was performed using Ingenuity^TM^ Pathways Analysis software. Of the 893 genes, 477 were mapped to gene networks defined by this tool. This analysis revealed a series of putative networks and associated functional categories. The 10 putative networks with the highest scores are listed in Table S1 and their associated functions are illustrated in Figure S2.

As expected, genes involved in carcinogenesis-related functions such as cellular growth and proliferation, cancer, cell death, and DNA-replication and -repair were enriched, providing confidence in our results. We also found that genes involved in inflammatory response, cell-mediated immune response, infectious disease, inflammatory disease, and immunological disease were also present in significant numbers. Interestingly, a significant abundance of genes involved in skeletal/muscular system development and disorders was observed (Figure S2).

**Figure 3:**
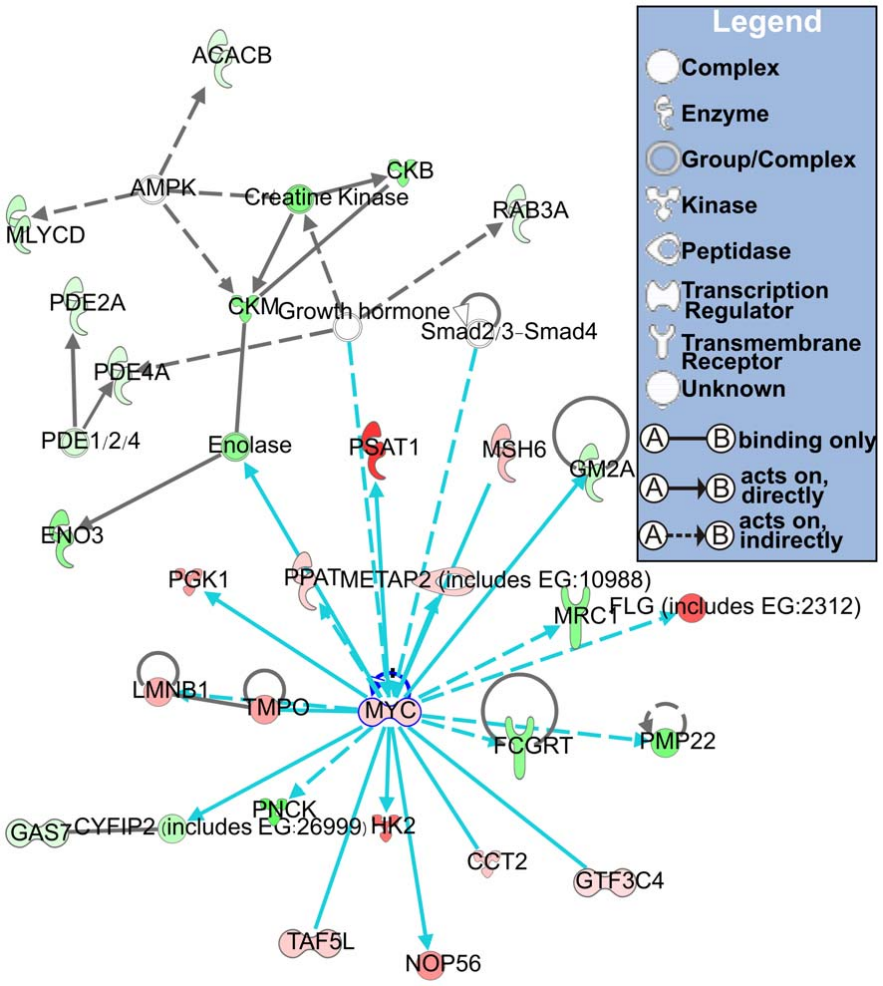
Gene networks enriched with genes modulated by nicotinamide in cancer prevention. Up- and down-regulated genes in the mouse bladder cancers are indicated in red and green, respectively. The intensity of the color is indicative of the degree of over- or under-expression. Genes without highlighted color are not part of the progression signature but are associated with the regulated genes. Each line and arrow represents functional and physical interactions between the genes and the direction of regulation reported in the literature.

Examination of the enriched genes revealed several important signaling networks (Table S1), the most striking of which showed predominant activation of the *Myc*, one of the oncogenic signatures (Figure 3). *Myc* formed the primary hub of the gene network, with the highest connectivity to the rest of the genes in the network, suggesting that *Myc* could be a critical molecular factor in both carcinogenesis induced by BBN and cancer prevention induced by nicotinamide. Many of the satellite genes connected to *Myc* (i.e., *MSH6* [*Msh6*], *METAP2* [*Metap2*], *LMNB1* [*Lmnb1*], *HK2* [*Hk2*], *PPAT* [*Ppat*], *PGK1* [*Pgk1*], *CCT2* [*Cct2*], and *PMP22* [*Pmp22*]) (Figure 3) participate in tumorigenesis, cell proliferation, cell growth, or apoptosis, which correspond to the best-known activities of *Myc*. These findings strongly indicated that oncogenic function of the *Myc* and its associated genes may be regulated by nicotinamide treatment to prevent bladder carcinogenesis.

### Validation of the *Myc* signature for cancer prevention by nicotinamide

**Figure 4:**
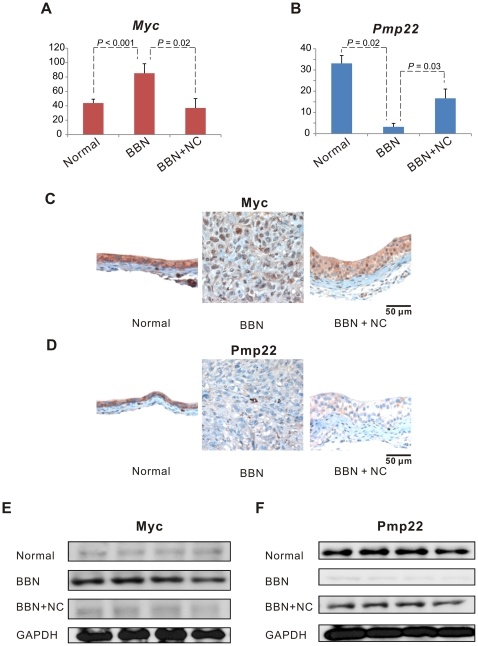
Validation of the gene exression signature in the mouse models (Normal, N-butyl-N-(4-hydroxybutyl)-nitrosamine [BBN], and BBN + nicotinamide [NC]). Real-time reverse transcriptase polymerase chain reaction (RT-PCR) analysis was performed for *Myc* (A) and *Pmp22* (B). Differences (*P* values) between mouse groups were obtained by two-sample t-test. The y-axis indicates (expression ratio of each gene / *Gapdh*) ×1000. Immunohistochemical analysis of Myc (C) and Pmp22 (D) was carried out to confirm protein expression levels. Detection of Myc (E) and Pmp22 (F) protein expression was also determined by Western blot analysis.

To validate our newly identified gene expression signature for cancer prevention by nicotinamide, we additionally carried out gene expression and protein expression analyses. Among the genes of the *Myc* signature identified by gene network analysis (Figure 3), *Myc* as a primary hub oncogene and *Pmp22* as a tumor-suppressed gene which has the strongest signal intensity were chosen for validation, and the results are presented in Table 1 and Figure 4.

**Table 1:** Protein expression levels of Myc and Pmp22 in the mouse model.

Experimental groups	Nucleic Myc (mean ± SD)	Cytoplasmic Myc (mean ± SD)	Nucleic Pmp22 (mean ± SD)	Cytoplasmic Pmp22 (mean ± SD)
Normal (n = 5)	0.4±0.54	4.6±1.34	0.4±0.54	10.8±1.64
NC (n = 5)	0.6±0.54	4.2±1.10	0.4±0.54	10.2±2.68
BBN (n = 5)	11.4±1.34	2.0±1.22	0.2±0.45	0.8±0.44
BBN + NC (n = 5)	2.2±2.28	3.2±1.09	0.6±0.55	9.2±3.89

The protein expression levels of Myc and Pmp22 were measured in the nucleus and cytoplasm. The *P*-values were obtained for the following categories: Normal vs. N-butyl-N-(4-hydroxybutyl)-nitrosamine (BBN) (Nucleic Myc, *P*<0.001; Cytoplasmic Pmp22, *P*<0.001), BBN vs. BBN + nicotinamide (NC) (Nucleic Myc, *P*<0.001; Cytoplasmic Pmp22, *P* = 0.001), and Normal vs. BBN + NC (Nucleic Myc, *P* = 0.12; Cytoplasmic Pmp22, *P* = 0.42). The *P*-values were calculated by two sample t-tests to each comparison. When applying correlation test between nucleic Myc and cytoplasmic Pmp22, two molecules were strongly inversely correlated. (*r* = −0.83, *P*<0.001). Correlation test was carried out by Pearson correlation test. SD indicates standard deviation.

We first performed gene expression analysis by RT-PCR. The expression levels of *Myc* in BBN-induced bladder cancer group were significantly higher than normal or BBN + nicotinamide-treated groups (*P*<0.001 and P = 0.02 by two sample t-test respectively, Figure 4, A). On the other hand, the expression levels of *Pmp22* in BBN-induced bladder cancer group were significantly decreased as compared with normal or BBN + nicotinamide-treated groups (*P* = 0.02 and P = 0.03 by two sample t-test respectively, Figure 4, B).

To determine whether gene expression signature we identified agree with protein expression levels, immunohistochemical analysis was carried out. We observed that Myc was highly expressed in the cytoplasm of normal mouse urothelium cells, but was not expressed in the nucleus. Myc was mainly localized to the nucleus of the tumor cells in the BBN-induced mouse bladder tumor. Both a nuclear and a cytoplasmic distribution of Myc was observed; however, the number of Myc-positive nuclei in the mouse urothelium treated with BBN and nicotinamide was significantly lower than in the BBN-induced bladder tumor, and the cytoplasmic staining intensity of Myc in either BBN group was weaker than that in normal urothelium (Figure 4, C and Table 1). We also found that Pmp22 was distributed in the cytoplasm of normal mouse urothelium, whereas there was no evidence of either cytoplasmic or nuclear expression of Pmp22 in the BBN-induced mouse bladder tumor. In the BBN + nicotinamide-treated urothelium, cytoplasmic Pmp22 expression was observed; however, the level of Pmp22 expression was reduced relative to that in normal urothelium (Figure 4, D and Table 1).

We also performed western blots for Myc and Pmp22 to rigorously verify our gene expression profiling analysis. On western blot analysis, Myc was highly expressed in the BBN-induced mouse bladder tumor group compared to normal and the BBN + nicotinamide-treated non-tumorigenic bladder group, but Myc expression did not show any difference between normal and the BBN + nicotinamide-treated non-tumorigenic bladder group. On the other hand, Pmp22 expression level in normal bladder group was significantly higher than in the BBN-induced mouse bladder tumor group. Although Pmp22 expression was observed in the BBN + nicotinamide-treated non-tumorigenic bladder group, the level of Pmp22 expression was reduced relative to that in normal. These results indicated that the changes in the protein expression levels of the *Myc* and its associated genes agreed well with the changes in the gene expression levels revealed by microarray analysis. The results also provided strong evidence for the reliability of the gene expression signature identification.

### Comparison of gene expression between human and mouse bladder cancers

**Figure 5:**
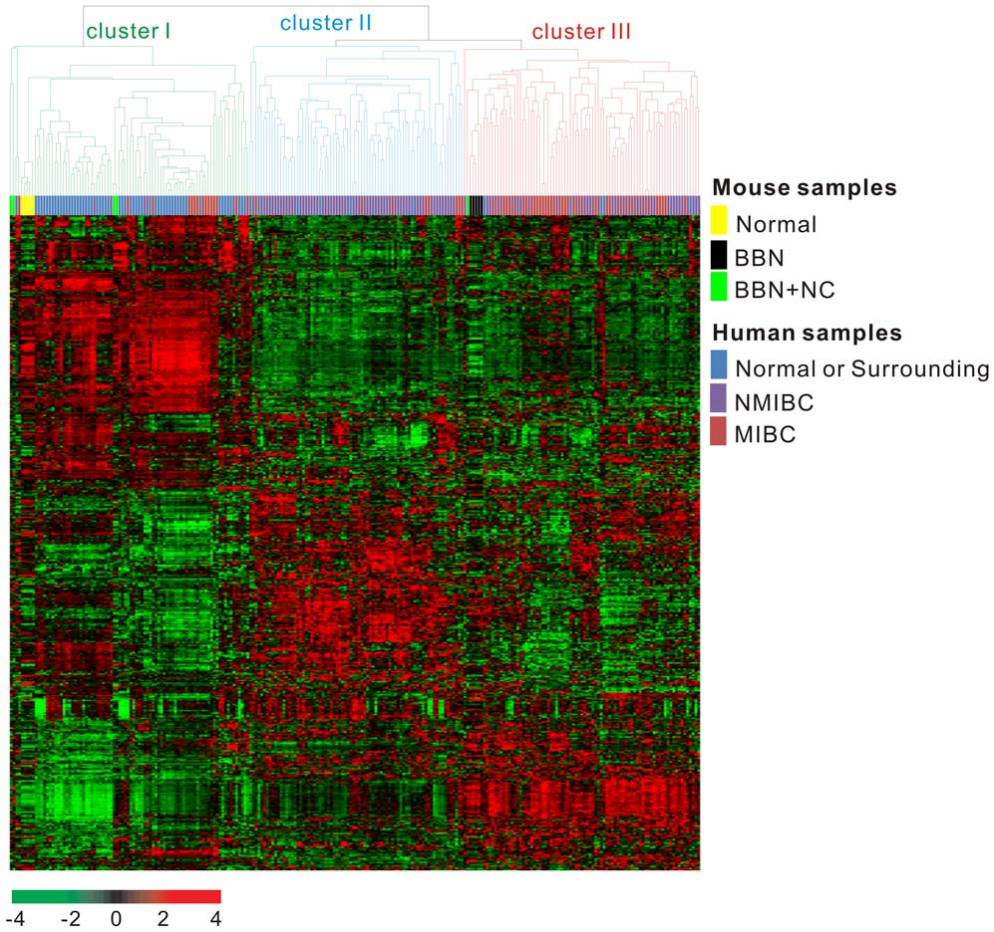
Unsupervised hierarchical clustering of mouse homologous genes with human cancers (Korean cohort). Genes with expression values that had a standard deviation of at least 0.9 were selected for hierarchical analysis (1,059 gene features). The red and green colors reflect high and low expression levels, respectively. BBN, NC, NMIBC, and MIBC indicate N-butyl-N-(4-hydroxybutyl)-nitrosamine, nicotinamide, non-muscle invasive bladder cancer, and muscle invasive bladder cancer, respectively.

Given the three distinct subgroups of the mouse experimental model, we examined how well these models recapitulate human bladder cancer phenotypes as defined by gene expression patterns. Because two different microarray platforms were used to study mouse and human bladder cancer (Korean cohort), we selected homologous genes that were present in both microarrays using HomoloGene, which provided information on homologous mouse and human genes. A total of 11,565 homologous genes were present in both microarrays. In hierarchical clustering analysis of the integrated data, the three subgroups were well separated from each other (Figure 5). Except for only one mouse sample, the gene expression patterns of the bladder in normal mice and BBN + nicotinamide-treated mice were very similar to those of normal mucosa or mucosa surrounding (adjacent to) cancers in human (cluster I in Figure 5). In contrast, the expression patterns of mouse bladder cancers from the BBN-induced tumor group were very similar to those of MIBCs in humans (cluster III in Figure 5).

We next applied supervised learning methods to validate the unsupervised cluster analysis. We applied four independent prediction methods to determine which of the mouse models might best mimic the human phenotypes. We used the gene expression data sets from the two subclasses of human bladders to train prediction methods (Normal vs. MIBC, NMIBC vs. MIBC, and Normal vs. NMIBC). All prediction methods predicted that most normal and BBN + nicotinamide-treated mouse bladders are relatively similar to normal mucosa or mucosa surrounding (adjacent to) cancers in human, whereas BBN-induced mouse bladder cancers are relatively similar to MIBC in human (Table 2). In addition, when we applied prediction methods trained with NMIBCs and MIBCs, we observed that all BBN-induced mouse bladder cancers were predicted as MIBC (Table 2). Furthermore, when applying prediction methods trained with normal and NMIBCs, most BBN + nicotinamide-treated mouse bladders were relatively similar to normal mucosa or mucosa surrounding (adjacent to) cancers in human (Table 2). The statistically significant gene lists of all comparisons (Normal vs. MIBC, NMIBC vs. MIBC, and Normal vs. NMIBC) were described in Table S3, S4, and S5, respectively. The biological implications of these genes in each comparison were also illustrated in Figure S4, S5, and S6, respectively. Consistent results were obtained when comparing mouse and another human bladder cancer dataset (Spanish cohort, Figure S3 and Table S2). Taken together, these data indicate that chemically-induced mouse bladder cancer models are very similar with human MIBCs and BBN + nicotinamide-treated mouse bladders are very similar with normal mucosa histologically and genetically, which supports the proposal that nicotinamide treatment might have a potential efficacy as chemo-preventive agent for MIBC development.

**Table 2:** Outcomes of the gene expression-based prediction methods (Korean cohort).

	CCP		LDA		NEC		SVM	
Predicted subclass	**NORMAL**	**MIBC**	**NORMAL**	**MIBC**	**NORMAL**	**MIBC**	**NORMAL**	**MIBC**
**Human samples**								
NORMAL (n = 68)	58	10	57	11	58	10	57	11
MIBC (n = 63)	14	49	14	49	14	49	12	51
Percentage correctly classified*	81.7		80.9		81.7		82.4	
**Mouse samples**								
NORMAL (n = 5)	5	0	5	0	5	0	4	1
BBN (n = 5)	0	5	0	5	0	5	1	4
BBN + NC (n = 5)	3	2	3	2	3	2	4	1
Percentage correctly classified†	86.7		86.7		86.7		80	
Predicted subclass	**NMIBC**	**MIBC**	**NMIBC**	**MIBC**	**NMIBC**	**MIBC**	**NMIBC**	**MIBC**
**Human samples**								
NMIBC (n = 102)	82	20	82	20	82	20	72	30
MIBC (n = 63)	17	46	17	46	17	46	28	35
Percentage correctly classified*	77.5		77.5		77.5		64.8	
**Mouse samples**								
BBN (n = 5)	0	5	0	5	0	5	0	5
Percentage correctly classified†	100		100		100		100	
Predicted subclass	**NORMAL**	**NMIBC**	**NORMAL**	**NMIBC**	**NORMAL**	**NMIBC**	**NORMAL**	**NMIBC**
**Human samples**								
NORMAL (n = 68)	59	9	59	9	60	8	60	8
NMIBC (n = 102)	5	97	5	97	5	97	7	95
Percentage correctly classified*	91.8		91.8		92.4		91.2	
**Mouse samples**								
BBN + NC (n = 5)	4	1	4	1	4	1	3	2
Percentage correctly classified†	80		80		80		60	

*Percentage for correct prediction during leave-one-out cross-validation.

† Percentage for histopathologically correct prediction of mouse tissues.

Abbreviations: CCP, compound covariate predictor; LDA, linear discriminator analysis; NEC, nearest centroid; SVM, support vector machines; NMIBC, non-muscle invasive bladder cancer; MIBC, muscle invasive bladder cancer; BBN, N-butyl-N-(4-hydroxybutyl)-nitrosamine; NC, nicotinamide.

## Discussion

In this study, we clearly demonstrated three different gene expression patterns among normal bladder, BBN-induced bladder tumor, and BBN + nicotinamide-treated non-tumorigenic bladder. Based on the results of the gene network analysis, we identified a putative molecular signature that might be responsible for bladder cancer prevention by nicotinamide, and the validity of this signature was confirmed by immunohistochemical analysis. In addition, through cross-species comparison of gene expression patterns, we identified similarity between the mouse bladder cancer model and human bladder cancer, which opens the gate to examining the usefulness of nicotinamide treatment for human bladder cancer.

The accumulated evidence from previous reports documenting the association between nicotinamide and human cancer implies that nicotinamide plays a key role in cancer prevention through its activities in cellular repair [10], [23], [24]. Recent study of nicotinamide suggests that nicotinamide deficiency is strongly associated with human cancers, and nicotinamide supplementation for cancer patients decreases the severity of side effects of chemotherapy [25], [26]. Furthermore, it was reported more recently that nicotinamide treatment increased the efficacy of radiotherapy in locally advanced bladder carcinoma [27]. However, to date, evidence towards identifying the genetic alterations of nicotinamide in cancer prevention has not been provided. Therefore, we investigated a potential functional association between nicotinamide and cancer prevention by performing gene expression profiling of a chemically-treated mouse bladder tumor model.

On the basis of the success of previous genome-wide gene expression profile studies in animal cancer models [9], [28], [29], [30], [31], we characterized different gene expression patterns among normal bladders, BBN-induced bladder cancers, and BBN + nicotinamide-treated non-tumorigenic bladders. We began by assessing the molecular characteristics of the different experimental groups via an unsupervised clustering analysis (Figure S1), and then applied a Venn diagram approach (Figure 2) to identify gene sets that were differentially expressed among the three mouse groups (normal, BBN, and BBN + nicotinamide). These results indicated that the gene expression pattern of each experimental group is strongly correlated with its histological classification, and the gene expression signature (893 genes in Figure 2) we identified presents the molecular characteristics of bladder cancer prevention by nicotinamide.

The identification of stable and reliable gene-to-gene relationships is an essential step towards unraveling the interactions and functional correlations between genes [32]. Therefore, we performed gene network analysis to identify the association of these 893 genes (Figure 2) with cancer prevention by nicotinamide. Interestingly, the *Myc* and its associated genes were the most important putative effectors of nicotinamide in preventing bladder carcinogenesis (Figure 3). This finding was further supported by immunohistochemical analysis, which showed that changes of protein levels in the *Myc* network agreed well with the microarray gene expression patterns (Table 1 and Figure 4). These data underscore the functional association between the *Myc* signature and nicotinamide activity in cancer prevention.

Based on the analysis of the gene expression signature in the context of gene networks, we identified a series of putative networks and functional categories associated with nicotinamide activity in cancer prevention (Table S1 and Figure S2). This functional enrichment test revealed that nicotinamide actively influences cell growth, cell proliferation, cancer, and the cell cycle, which are the well-known characteristics of *Myc*. Because *Myc* is an independent predictor of bladder cancer progression and an important gene in the bladder cancer signaling pathway [33], [34], the results strongly support the potential involvement of *Myc* in the prevention of bladder cancer by nicotinamide. It was reported previously that nicotinamide could epigenetically stabilize *RUNX3*, one of the important tumor suppressors [35]. Interestingly, another study demonstrated that transcriptional activity of the *MYC* promoter was down-regulated by up-regulation of *RUNX3* in colorectal cancer cells [36]. Moreover, RUNX3 is a direct binding partner of histone deacetylase 2 (HDAC2) [37], transcriptional up-regluation of which promotes *Myc*-induced oncogeneic effects [38]. Thus, we putatively suggest that nicotinamide treatment may be effective in decreasing the risk of bladder cancer progression by regulation of *Myc* which is modulated by epigenetic alteration of *Runx3*, although further experimental validation is needed.

The comparison of evolutionarily-conserved genes across different species has been frequently used to infer the function or evolutionary origin of a gene or protein. Cross-species comparative profiling of the tumor-related expression patterns of homologous genes has been applied in several human cancer studies including those examining liver cancer [29], [31], [39], lung cancer [40], breast cancer [41], and bladder cancer [9]. In the current study, we investigated the potential value of nicotinamide in the prevention of human cancer based on comparative analysis of global expression patterns of homologous genes in human and mouse bladder cancer. When compared with human gene expression patterns, those of BBN-induced mouse bladder cancers were more similar to those of invasive than non-invasive human cancers, and those of the BBN + nicotinamide-treated group were most similar to normal tissues in humans (Figure 5 and Table 2). These results not only illustrated a strong correlation between human and mouse gene expression patterns, but also demonstrated the potential utility of nicotinamide as a cancer preventive agent for MIBC. It was further supported by a recent report that radiotherapy with nicotinamide treatment significantly reduced the risk of disease progression and death of MIBC [27].

Our results based on gene expression profiling of a mouse bladder cancer model demonstrate that nicotinamide may be a reliable agent of cancer prevention, via modulation of the *Myc* and its associated genes. The use of nicotinamide could be a potentially valuable chemo-preventive and therapeutic approach for prevention of bladder cancer, and may improve the effectiveness of currently available treatments.

## Supporting Information

Figure S1 **Gene expression patterns of the mouse bladder cancer model.** Genes were selected when at least 5 samples have detection *P*-value less than 0.05 at each probe. The detection *P*-value was provided by Genome Studio ^TM^ software from Illumina, Inc. A total of 28,253 probes (21,611 genes) were selected in this analysis. After filtering of probes, the data were normalized by quantile normalization method, log2-transformed, and median-centered across genes and samples. Finally, probes with expression values that had a standard deviation of at least 0.5 were selected. A total of 6,659 probes (5,643 genes) were selected in the cluster analysis. The red and green colors reflect high and low expression levels, respectively. BBN indicates N-butyl-N-(4-hydroxybutyl)-nitrosamine.
(TIF)

Figure S2 **Functional classification of the genes modulated by nicotinamide in cancer prevention.** Classification enrichment was determined using Ingenuity Pathway Analysis software. The threshold of significance was -log (*P* = 0.05).
(TIF)

Figure S3 **Unsupervised hierarchical clustering of mouse homologous genes with human cancers (Spanish cohort).** Genes with expression values that had a standard deviation of at least 0.9 were selected for hierarchical analysis (1,414 gene features). The red and green colors reflect high and low expression levels, respectively. BBN indicates N-butyl-N-(4-hydroxybutyl)-nitrosamine.
(TIF)

Figure S4 **Functional classification of the significant genes applied to gene expression-based prediction methods (Comparison between normal and MIBC in human).** Top 20 highly scored biological functions were illustrated. Genes involved in carcinogenesis-related functions such as cancer, cell growth & proliferation, cell death, and DNA-replication and -repair were enriched. Genes involved in immune cell trafficking, inflammatory response, and immunological disease were also present in significant numbers, consistent with function enrichment test of mouse data (Figure S3). Classification enrichment was determined using Ingenuity Pathway Analysis software. The threshold of significance was −log (*P* = 0.05).
(TIF)

Figure S5 **Functional classification of the significant genes applied to gene expression-based prediction methods (Comparison between NMIBC and MIBC in human).** Top 20 highly scored biological functions were illustrated. It is found that genes involved in carcinogenesis-related functions such as cancer, cell growth & proliferation, cell cycle were enriched. Interestingly, genes involved in renal and urological system development were also significantly enriched. Classification enrichment was determined using Ingenuity Pathway Analysis software. The threshold of significance was −log (*P* = 0.05).
(TIF)

Figure S6 **Functional classification of the significant genes applied to gene expression-based prediction methods (Comparison between normal and NMIBC in human).** Top 20 highly scored biological functions were illustrated. Genes involved in carcinogenesis-related functions such as cancer, cell growth & proliferation, cell death, and DNA-replication and -repair were enriched. Genes involved in immune- or inflammation-associated functions such as immune cell trafficking, inflammatory response, immunological disease, and inflammatory disease were also present in significant numbers. Classification enrichment was determined using Ingenuity Pathway Analysis software. The threshold of significance was −log (*P* = 0.05).
(TIF)

Table S1 **Top 10 list of gene networks from Ingenuity^TM^ Pathway Analysis.**
(DOC)

Table S2 **Outcomes of the gene expression-based prediction methods (Spanish cohort).**
(DOC)

Table S3 **Significant gene list (510 genes) applied to gene expression-based prediction methods (Comparison between Normal and MIBC in human).**
(DOC)

Table S4 **Significant gene list (417 genes) applied to gene expression-based prediction methods (Comparison between NMIBC and MIBC in human).**
(DOC)

Table S5 **Significant gene list (623 genes) applied to gene expression-based prediction methods (Comparison between Normal and NMIBC in human).**
(DOC)

## References

[1] Wynder EL, Goldsmith R (1977). The epidemiology of bladder cancer: a second look.. Cancer.

[2] Howe GR, Burch JD, Miller AB, Cook GM, Esteve J (1980). Tobacco use, occupation, coffee, various nutrients, and bladder cancer.. J Natl Cancer Inst.

[3] Cole P, Hoover R, Friedell GH (1972). Occupation and cancer of the lower urinary tract.. Cancer.

[4] Matanoski GM, Elliott EA (1981). Bladder cancer epidemiology.. Epidemiol Rev.

[5] Grubbs CJ, Lubet RA, Koki AT, Leahy KM, Masferrer JL (2000). Celecoxib inhibits N-butyl-N-(4-hydroxybutyl)-nitrosamine-induced urinary bladder cancers in male B6D2F1 mice and female Fischer-344 rats.. Cancer Res.

[6] Grubbs CJ, Moon RC, Squire RA, Farrow GM, Stinson SF (1977). 13-cis-Retinoic acid: inhibition of bladder carcinogenesis induced in rats by N-butyl-N-(4-hydroxybutyl)nitrosamine.. Science.

[7] Druckrey H, Preussmann R, Ivankovic S, Schmidt CH, Mennel HD (1964). [Selective Induction of Bladder Cancer in Rats by Dibutyl- and N-Butyl-N-Butanol(4)-Nitrosamine.].. Z Krebsforsch.

[8] Ohtani M, Kakizoe T, Nishio Y, Sato S, Sugimura T (1986). Sequential changes of mouse bladder epithelium during induction of invasive carcinomas by N-butyl-N-(4-hydroxybutyl)nitrosamine.. Cancer Res.

[9] Williams PD, Lee JK, Theodorescu D (2008). Molecular credentialing of rodent bladder carcinogenesis models.. Neoplasia.

[10] Jacobson EL, Jacobson MK (1993). A biomarker for the assessment of niacin nutriture as a potential preventive factor in carcinogenesis.. J Intern Med.

[11] Chen J, Cui X, Zacharek A, Ding GL, Shehadah A (2009). Niaspan treatment increases tumor necrosis factor-alpha-converting enzyme and promotes arteriogenesis after stroke.. J Cereb Blood Flow Metab.

[12] Wu BJ, Yan L, Charlton F, Witting P, Barter PJ (2010). Evidence that niacin inhibits acute vascular inflammation and improves endothelial dysfunction independent of changes in plasma lipids.. Arteriosclerosis, thrombosis, and vascular biology.

[13] Gurujeyalakshmi G, Wang Y, Giri SN (2000). Taurine and niacin block lung injury and fibrosis by down-regulating bleomycin-induced activation of transcription nuclear factor-kappaB in mice.. J Pharmacol Exp Ther.

[14] Lee JS, Leem SH, Lee SY, Kim SC, Park ES (2010). Expression signature of E2F1 and its associated genes predict superficial to invasive progression of bladder tumors.. J Clin Oncol.

[15] Sanchez-Carbayo M, Socci ND, Lozano J, Saint F, Cordon-Cardo C (2006). Defining molecular profiles of poor outcome in patients with invasive bladder cancer using oligonucleotide microarrays.. J Clin Oncol.

[16] Eisen MB, Spellman PT, Brown PO, Botstein D (1998). Cluster analysis and display of genome-wide expression patterns.. Proc Natl Acad Sci U S A.

[17] Wright GW, Simon RM (2003). A random variance model for detection of differential gene expression in small microarray experiments.. Bioinformatics.

[18] Sayers EW, Barrett T, Benson DA, Bryant SH, Canese K (2009). Database resources of the National Center for Biotechnology Information.. Nucleic Acids Res.

[19] Radmacher MD, McShane LM, Simon R (2002). A paradigm for class prediction using gene expression profiles.. J Comput Biol.

[20] Dudoit S, Fridlyand J, Speed TP (2002). Comparison of Discrimination Methods for the Classification of Tumors Using Gene Expression Data.. J Am Stat Assoc.

[21] Ramaswamy S, Tamayo P, Rifkin R, Mukherjee S, Yeang CH (2001). Multiclass cancer diagnosis using tumor gene expression signatures.. Proc Natl Acad Sci U S A.

[22] Simon R, Radmacher MD, Dobbin K, McShane LM (2003). Pitfalls in the use of DNA microarray data for diagnostic and prognostic classification.. J Natl Cancer Inst.

[23] Kirkland JB (2003). Niacin and carcinogenesis.. Nutr Cancer.

[24] Jacobson EL, Dame AJ, Pyrek JS, Jacobson MK (1995). Evaluating the role of niacin in human carcinogenesis.. Biochimie.

[25] Shah GM, Shah RG, Veillette H, Kirkland JB, Pasieka JL (2005). Biochemical assessment of niacin deficiency among carcinoid cancer patients.. Am J Gastroenterol.

[26] Kirkland JB (2009). Niacin status and treatment-related leukemogenesis.. Mol Cancer Ther.

[27] Hoskin PJ, Rojas AM, Bentzen SM, Saunders MI (2010). Radiotherapy with concurrent carbogen and nicotinamide in bladder carcinoma.. Journal of clinical oncology: official journal of the American Society of Clinical Oncology.

[28] Yao R, Lemon WJ, Wang Y, Grubbs CJ, Lubet RA (2004). Altered gene expression profile in mouse bladder cancers induced by hydroxybutyl(butyl)nitrosamine.. Neoplasia.

[29] Lee JS, Chu IS, Mikaelyan A, Calvisi DF, Heo J (2004). Application of comparative functional genomics to identify best-fit mouse models to study human cancer.. Nat Genet.

[30] Yao R, Yi Y, Grubbs CJ, Lubet RA, You M (2007). Gene expression profiling of chemically induced rat bladder tumors.. Neoplasia.

[31] Lee JS, Heo J, Libbrecht L, Chu IS, Kaposi-Novak P (2006). A novel prognostic subtype of human hepatocellular carcinoma derived from hepatic progenitor cells.. Nat Med.

[32] Prieto C, Risueno A, Fontanillo C, De las Rivas J (2008). Human gene coexpression landscape: confident network derived from tissue transcriptomic profiles.. PLoS One.

[33] Baffa R, Letko J, McClung C, LeNoir J, Vecchione A (2006). Molecular genetics of bladder cancer: targets for diagnosis and therapy.. J Exp Clin Cancer Res.

[34] Schultz L, Albadine R, Hicks J, Jadallah S, DeMarzo AM (2010). Expression status and prognostic significance of mammalian target of rapamycin pathway members in urothelial carcinoma of urinary bladder after cystectomy.. Cancer.

[35] Kim WJ, Lee JW, Quan C, Youn HJ, Kim HM (2011). Nicotinamide Inhibits Growth of Carcinogen Induced Mouse Bladder Tumor and Human Bladder Tumor Xenograft Through Up-Regulation of RUNX3 and p300.. The Journal of urology.

[36] Lee CW, Ito K, Ito Y (2010). Role of RUNX3 in bone morphogenetic protein signaling in colorectal cancer.. Cancer research.

[37] Jin YH, Jeon EJ, Li QL, Lee YH, Choi JK (2004). Transforming growth factor-beta stimulates p300-dependent RUNX3 acetylation, which inhibits ubiquitination-mediated degradation.. J Biol Chem.

[38] Marshall GM, Gherardi S, Xu N, Neiron Z, Trahair T (2010). Transcriptional upregulation of histone deacetylase 2 promotes Myc-induced oncogenic effects.. Oncogene.

[39] Lam SH, Wu YL, Vega VB, Miller LD, Spitsbergen J (2006). Conservation of gene expression signatures between zebrafish and human liver tumors and tumor progression.. Nat Biotechnol.

[40] Sweet-Cordero A, Mukherjee S, Subramanian A, You H, Roix JJ (2005). An oncogenic KRAS2 expression signature identified by cross-species gene-expression analysis.. Nat Genet.

[41] Herschkowitz JI, Simin K, Weigman VJ, Mikaelian I, Usary J (2007). Identification of conserved gene expression features between murine mammary carcinoma models and human breast tumors.. Genome Biol.

