# Supplementary figures and images for: Identification of Gene Expression Signature Modulated by Nicotinamide in a Mouse Bladder Cancer Model
Source: PLoS One. 2011 Oct 10;6(10):e26131. doi: 10.1371/journal.pone.0026131 (PMC3189956; doi:10.1371/journal.pone.0026131)

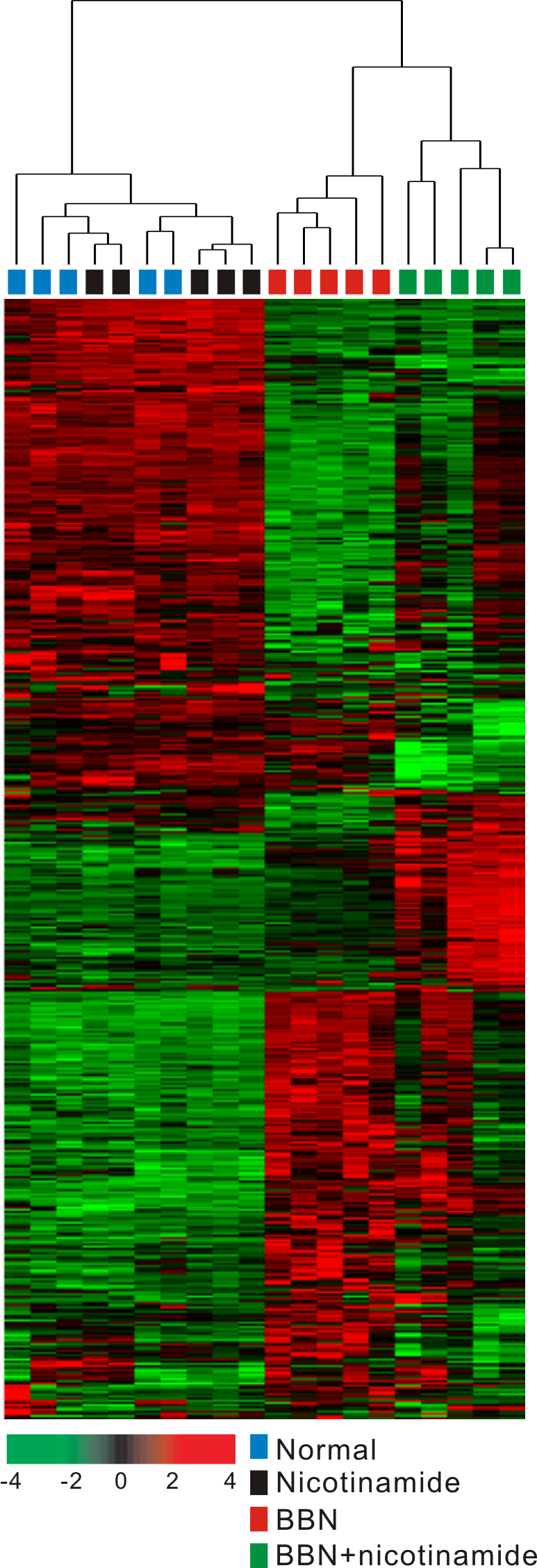

Supplement: Figure S1 — Gene expression patterns of the mouse bladder cancer model. Genes were selected when at least 5 samples have detection P-value less than 0.05 at each probe. The detection P-value was provided by Genome Studio TM software from Illumina, Inc. A total of 28,253 probes (21,611 genes) were selected in this analysis. After filtering of probes, the data were normalized by quantile normalization method, log2-transformed, and median-centered across genes and samples. Finally, probes with expression values that had a standard deviation of at least 0.5 were selected. A total of 6,659 probes (5,643 genes) were selected in the cluster analysis. The red and green colors reflect high and low expression levels, respectively. BBN indicates N-butyl-N-(4-hydroxybutyl)-nitrosamine. (TIF) [file pone.0026131.s001.tif]

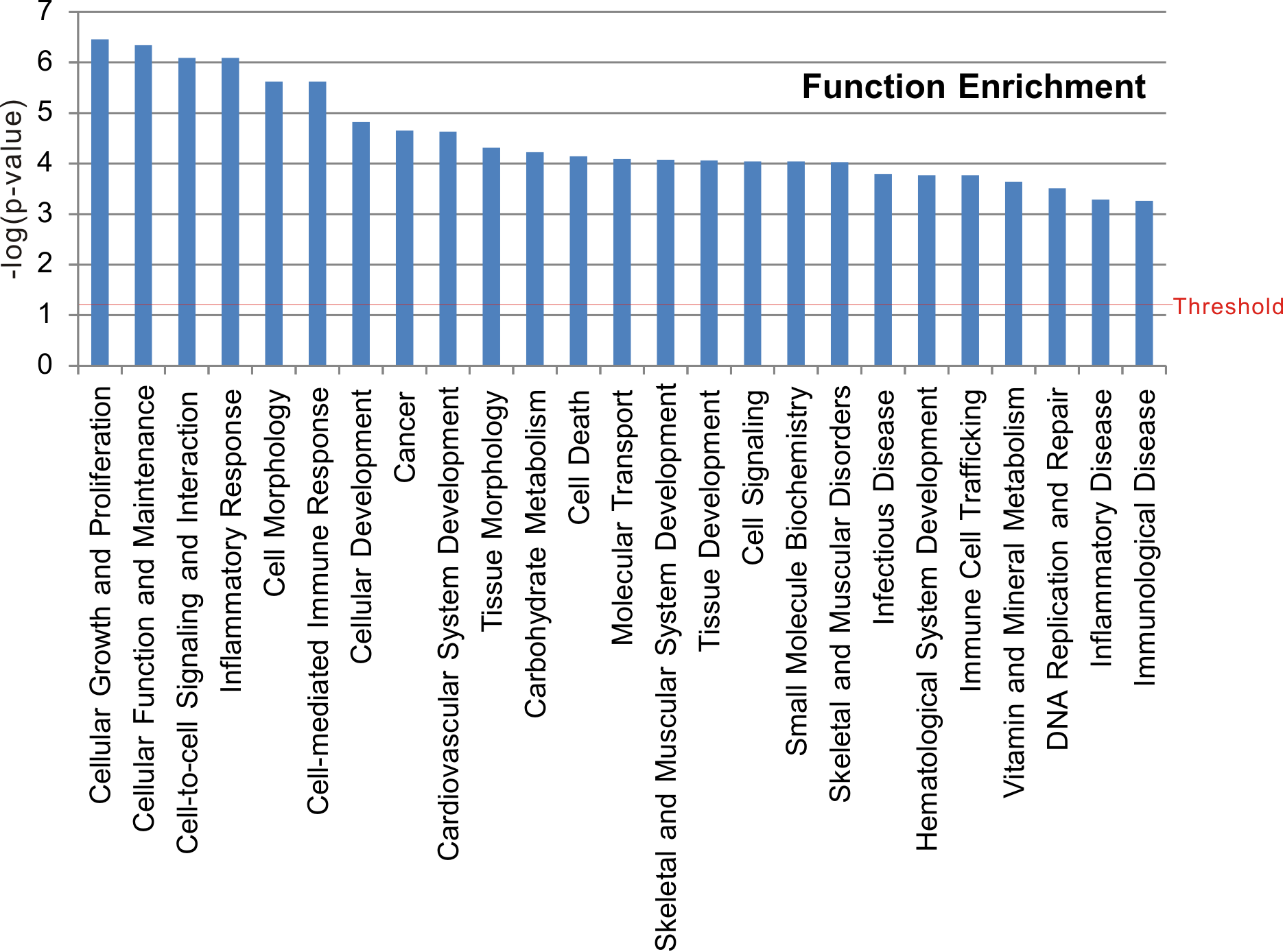

Supplement: Figure S2 — Functional classification of the genes modulated by nicotinamide in cancer prevention. Classification enrichment was determined using Ingenuity Pathway Analysis software. The threshold of significance was -log (P = 0.05). (TIF) [file pone.0026131.s002.tif]

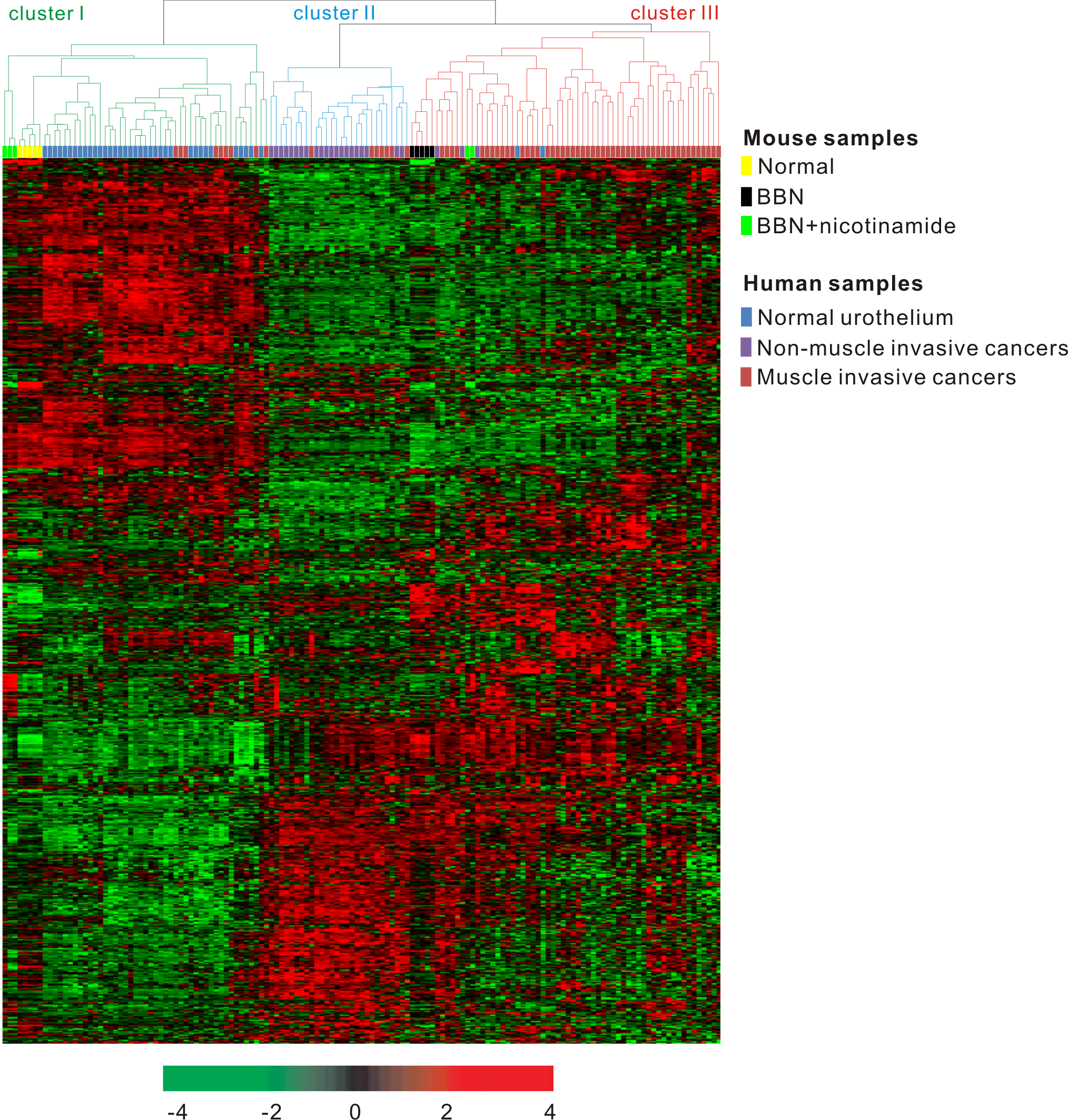

Supplement: Figure S3 — Unsupervised hierarchical clustering of mouse homologous genes with human cancers (Spanish cohort). Genes with expression values that had a standard deviation of at least 0.9 were selected for hierarchical analysis (1,414 gene features). The red and green colors reflect high and low expression levels, respectively. BBN indicates N-butyl-N-(4-hydroxybutyl)-nitrosamine. (TIF) [file pone.0026131.s003.tif]

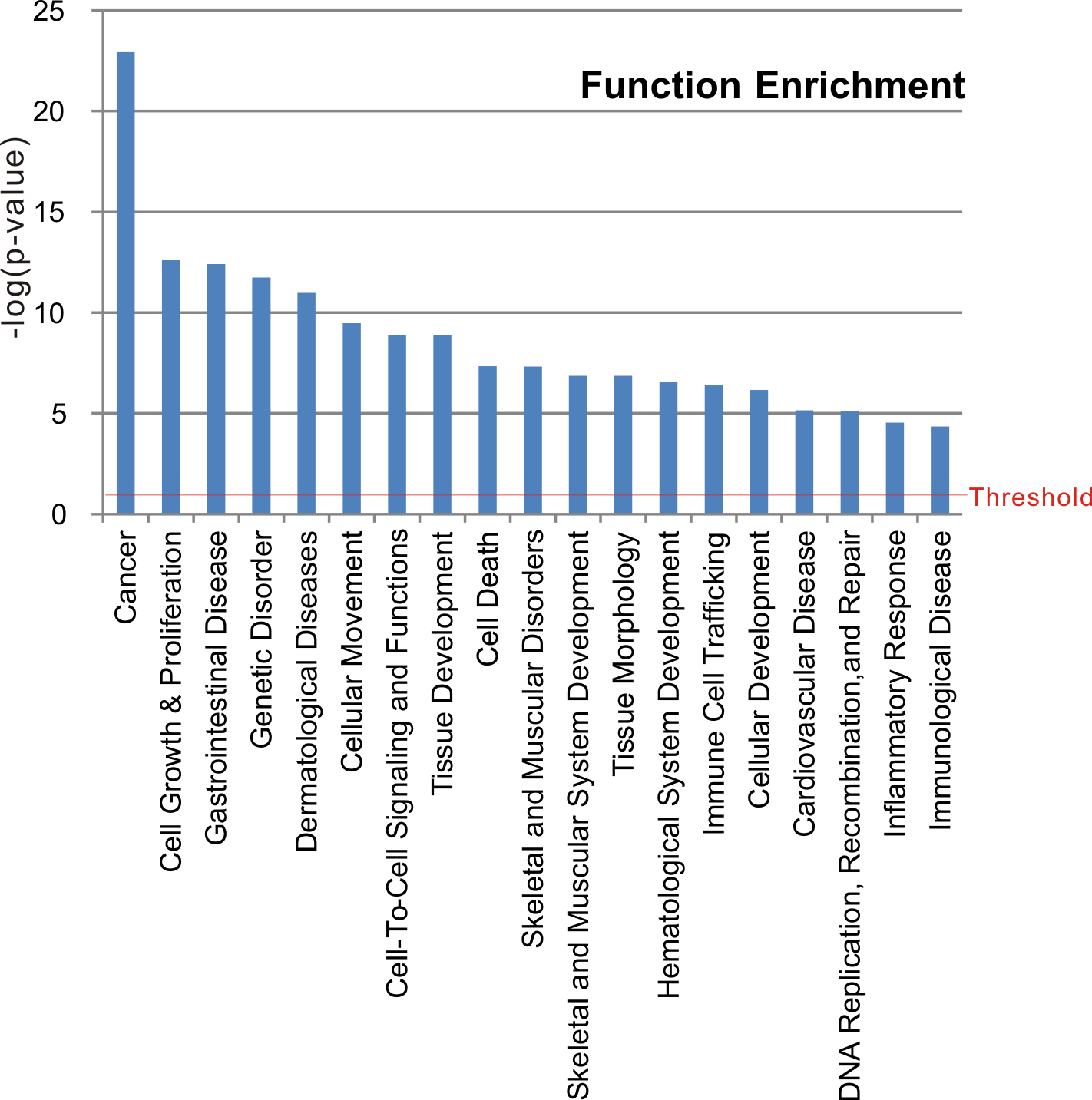

Supplement: Figure S4 — Functional classification of the significant genes applied to gene expression-based prediction methods (Comparison between normal and MIBC in human). Top 20 highly scored biological functions were illustrated. Genes involved in carcinogenesis-related functions such as cancer, cell growth & proliferation, cell death, and DNA-replication and -repair were enriched. Genes involved in immune cell trafficking, inflammatory response, and immunological disease were also present in significant numbers, consistent with function enrichment test of mouse data (Figure S3). Classification enrichment was determined using Ingenuity Pathway Analysis software. The threshold of significance was −log (P = 0.05). (TIF) [file pone.0026131.s004.tif]

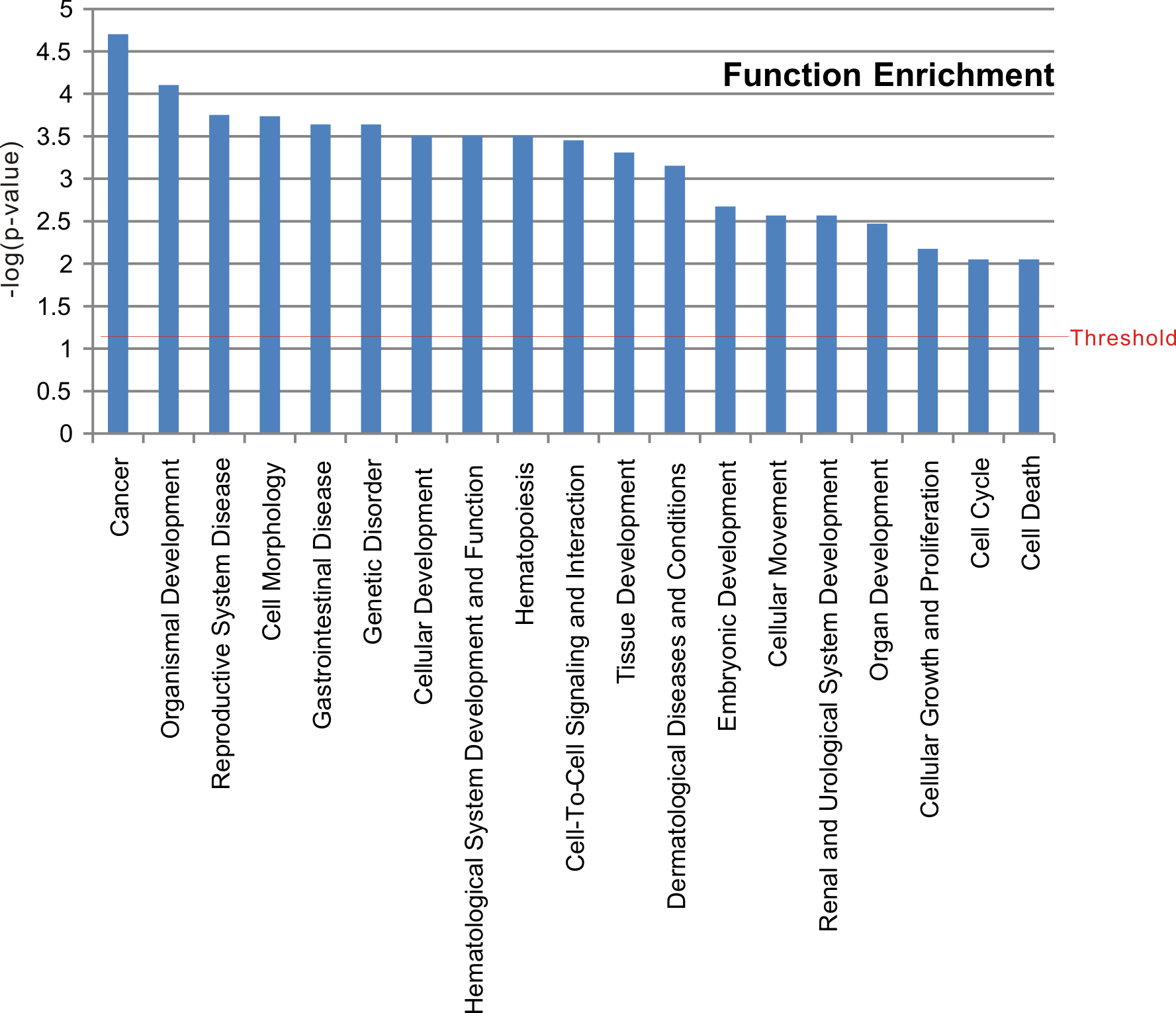

Supplement: Figure S5 — Functional classification of the significant genes applied to gene expression-based prediction methods (Comparison between NMIBC and MIBC in human). Top 20 highly scored biological functions were illustrated. It is found that genes involved in carcinogenesis-related functions such as cancer, cell growth & proliferation, cell cycle were enriched. Interestingly, genes involved in renal and urological system development were also significantly enriched. Classification enrichment was determined using Ingenuity Pathway Analysis software. The threshold of significance was −log (P = 0.05). (TIF) [file pone.0026131.s005.tif]

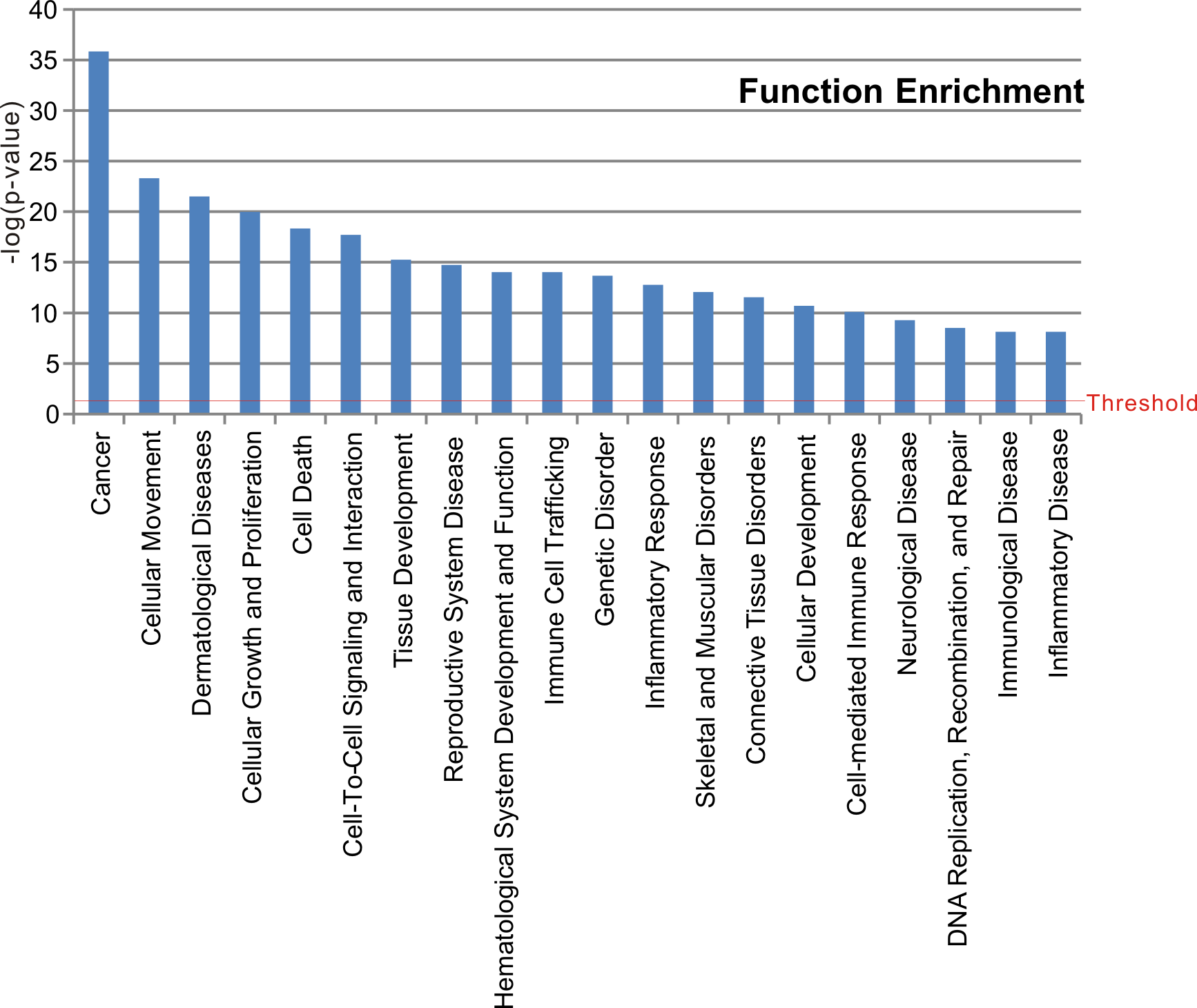

Supplement: Figure S6 — Functional classification of the significant genes applied to gene expression-based prediction methods (Comparison between normal and NMIBC in human). Top 20 highly scored biological functions were illustrated. Genes involved in carcinogenesis-related functions such as cancer, cell growth & proliferation, cell death, and DNA-replication and -repair were enriched. Genes involved in immune- or inflammation-associated functions such as immune cell trafficking, inflammatory response, immunological disease, and inflammatory disease were also present in significant numbers. Classification enrichment was determined using Ingenuity Pathway Analysis software. The threshold of significance was −log (P = 0.05). (TIF) [file pone.0026131.s006.tif]
