# Supplementary material for: Identification of Gene Expression Signature Modulated by Nicotinamide in a Mouse Bladder Cancer Model
Source: PLoS One. 2011 Oct 10;6(10):e26131. doi: 10.1371/journal.pone.0026131 (PMC3189956; doi:10.1371/journal.pone.0026131)
Supplement: Table S1 — Top 10 list of gene networks from IngenuityTM Pathway Analysis. (DOC) [file pone.0026131.s007.doc]

**Table S1. Top 10 list of gene networks from Ingenuity^TM^ Pathway Analysis**

| **Network ID** | ***Score** | **# of genes in list** | **Genes in network** |
| --- | --- | --- | --- |
| 1 | 46 | 29 | **↓ACACB,** AMPK**, ↑CCT2, ↓CKB, ↓CKM,** Creatine Kinase**, ↓CYFIP2 (includes EG:26999), ↓ENO3,** Enolase**, ↓FCGRT, ↑FLG (includes EG:2312), ↓GAS7, ↓GM2A,** Growth hormone**, ↑GTF3C4, ↑HK2, ↑LMNB1, ↑METAP2 (includes EG:10988), ↓MLYCD, ↓MRC1, ↑MSH6, ↑MYC, ↑NOP56,** PDE1/2/4**, ↓PDE2A, ↓PDE4A, ↑PGK1, ↓PMP22, ↓PNCK, ↑PPAT, ↑PSAT1, ↓RAB3A,** Smad2/3-Smad4**, ↑TAF5L, ↑TMPO** |
| 2 | 42 | 28 | **↓AKAP12, ↓BICC1, ↓CSK,** ELK3**, ↓FBLN1, ↑FGR, ↑FRG1,** Gata**, ↓GATA6,** Glutathione peroxidase**, ↑GTF2H1**, hCG**, ↓HCK, ↑KHDRBS1, ↓KLF2, ↓LMO2, ↓LTC4S, ↓LYL1, ↑NUBP2,** P38 MAPK**, ↓PKIA, ↓PTP4A3, ↓RHOJ, ↓SATB1,** Scf**,** SRC**, ↑STRAP, ↓TAL1, ↑TNPO2, ↓TUBG2,** Vegf**, ↑WEE1, ↓WIPF1, ↑XPO7, ↑ZW10** |
| 3 | 35 | 25 | **↓ACTG2 (includes EG:72), ↓ATP1A2, ↓ATP1B2, ↓C1QTNF2, ↑CDK6,** Collagen(s)**,** Complement component 1**, ↑EFNA1,** ERK**,** Fibrin**, ↓GYPC, ↓IL17RD,** Integrin**,** Integrin&alpha;**, ↑ITGA3, ↓ITGA9, ↑ITGAV, ↓LAMA2, ↓LAMC1, ↑LAMC2,** Laminin**,** Laminin2**,** Metalloprotease**, ↓MFN2, ↓NID1, ↓NTN1,** Pdgfr**, ↑PDIA3, ↑PIK3CB, ↓SERPING1, ↑SOCS6, ↓UNC5C, ↑VAV3, ↓VEGFC, ↓VTN** |
| 4 | 34 | 24 | **↓ABCA1, ↓APOE, ↓CCBP2, ↓CCL8, ↓CD36, ↓CITED4,** Fibrinogen**, ↓G0S2, ↓GFPT2, HDL, ↓HSD11B1, ↑IGFBP3,** Integrin alpha 3 beta 1**, ↑KLF3, LDL, ↓LSP1, ↑MEFV,** NCOR-LXR-Oxysterol-RXR-9 cis RA**, ↓NDN,** NFkB (complex)**, ↑NR2C1,** Pi4k**, ↑PI4K2B, ↓PI4KA, ↑PMAIP1,** PRKAC**,** Rar**, ↑RPL7A,** Rxr**, ↓SMPD2, ↑ST14, ↓STX12, ↓SYP, ↓TGFBR3,** Thyroid hormone receptor |
| 5 | 30 | 22 | 3',5'-cyclic-nucleotide phosphodiesterase**, ↑ADAM10, ↓ADAP1,** ADCY**, ↓ADCY5, ↓ADCY9, ↓ADCYAP1R1, ↑ARF5,** G**,** G alphai, G-protein beta**, ↓GNB4,** GUCY**, ↓GUCY1A3, ↓GUCY1B3, ↓KCNC4, ↑LPAR2, ↑LTB4R2,** Mapk**, ↓NPR2,** Pde**, ↓PDE4B, ↑PDE7A,** Pka**,** Pkc(s)**,** PLC**,** PLC gamma**,** Pld**, ↑PRKAR2B, ↓PRKCB, ↓PTGIR, ↓RAB11FIP3, ↓RGS2, ↑SMO, ↓TBXA2R** |
| 6 | 30 | 22 | **↓ACTA1,** Actin**, ↓ACTN2, ↓AIP,** Alcohol group acceptor phosphotransferase**, ↑ARL4C, ↓ASB2,** Calcineurin protein(s)**,** Calpain**,** Caspase**, ↑CDC34 (includes EG:997), Ck2, ↑CSNK1D,** Cytochrome c**, ↑EPS8L2, ERK1/2,** FSH**, ↓HCRTR1, ↑HNRNPA1,** Hsp70**,** Hsp90**, ↓IGFBP4, ↑NOP2, ↓P2RX1, ↓PDLIM3, ↓PDLIM4, ↓PHACTR1, ↑PRKX,** Proteasome**, ↑PSMD10, ↑PSME2, ↓RASSF2,** SGK1**,** TGIF1**,** Ubiquitin |
| 7 | 30 | 23 | Akt**, ↓BANK1,** Calcineurin A**,** Calmodulin**,** CaMKII**, ↓DES,** F Actin**, ↓GRB10, ↓ITPR1,** Mlc**, ↓MRVI1, ↓MYH10, ↓MYH11, ↓MYL4, ↓MYL9 (includes EG:10398), ↓MYLK, ↓MYO7A,** Myosin**,** Pak**,** Pkg**, ↓PLEKHB1, ↓PLN,** PP1**,** Pp2b**, ↓PPP1R12A, ↓PPP1R1A, ↓PPP1R3C, ↓PPP3CB, ↓PPP3CC, Rock, ↑RUNX1, ↓STK17B, ↓SYNM, ↓SYT11, ↑TRPM6** |
| 8 | 26 | 20 | Adaptor protein 2**, ↑ADM, ↑ANXA7,** AOC3**, ↑AP2B1, ↓ATP2A3, ↓CALCRL, ↓CD34,** Cyclin A**,** Cyclin B**,** Cyclin D**,** Cyclin E**,** E2f**, ↑EIF4B, ↓ENSA, ↓GPR182, ↓IL6ST,** Insulin**,** MAP2K1/2**,** Pdgf**,** PDGF BB**, ↓PDGFRA, ↑PDK1,** PI3K**, ↓PODXL,** PP2A**,** Rb**, ↑RBL1, ↑RPS6KB1,** Shc**, ↑SMARCC1, ↓SMARCD3, ↓TBX5, ↓TK2,** tyrosine kinase |
| 9 | 25 | 20 | **↓ADAM19,** Ap1**, ↓C2,** C1q**, ↑C1QBP, ↓C1R, ↓C1S,** CD44**,** COL14A1**, ↑DSG3, ↓FAAH, ↓FGL2, ↑FURIN,** IFN Beta**,** IgG**,** IKK (complex)**,** IL1**,** IL12 (complex)**,** Il12 (family)**,** Interferon alpha**,** MHC Class I (complex)**,** MHC CLASS I (family), Mmp**, ↓MMP17, ↓MMRN1, ↓NAALAD2, ↑PADI4, ↑PCSK4,** peptidase**, ↓PLP1,** Tgf beta**, ↓TLR7, ↑TMPRSS4, ↑TNFRSF11B,** Trypsin |
| 10 | 24 | 19 | **↓ADAMTS5,** ALB**,** ARTN**,** CRB1**,** DGKQ**, ↓DTX1, ↑EXOC5, ↓EXOC6,** F2**,** FAP**, ↓FHL1,** FXR2**, ↓GDF1, ↓GFRA3,** GJC2**,** HIVEP3**,** HTT**,** IL1B**, ↑KCNRG, ↑MPP5,** MTSS1**, ↓MYH11, ↓ MYL4,** PLA2G6**,** RET**, ↓SCARA3, ↓SDK1, ↓SEPP1, ↓SERPING1, ↓SFXN4,** SHH**,** SOD1**,** ST13**, ↓TBX1,** TSC22D1 |

* The score is a numerical value used to rank networks according to how relevant they are to the genes in the input dataset (893 genes). The score takes into account the number of genes in the network and the size of the network to approximate how relevant this network is to the input gene list.
