# Supplementary material for: Identification of Gene Expression Signature Modulated by Nicotinamide in a Mouse Bladder Cancer Model
Source: PLoS One. 2011 Oct 10;6(10):e26131. doi: 10.1371/journal.pone.0026131 (PMC3189956; doi:10.1371/journal.pone.0026131)
Supplement: Table S2 — Outcomes of the gene expression-based prediction methods (Spanish cohort). (DOC) [file pone.0026131.s008.doc]

**Table S2. Outcomes of the gene expression-based prediction methods (Spanish cohort)**

|  | **CCP** | | | **LDA** | | | **NEC** | | | **SVM** | |
| --- | --- | --- | --- | --- | --- | --- | --- | --- | --- | --- | --- |
| Predicted subclass | **NORMAL** | **MIBC** | | **NORMAL** | **MIBC** | | **NORMAL** | | **MIBC** | **NORMAL** | **MIBC** |
| **Human samples** |  |  | |  |  | |  | |  |  |  |
| NORMAL (n=38) | 36 | 2 | | 36 | 2 | | 36 | | 2 | 33 | 5 |
| MIBC (n=66) | 10 | 56 | | 10 | 56 | | 10 | | 56 | 5 | 61 |
| Percentage correctly classified* | 88.4 | | | 88.4 | | | 88.4 | | | 90.3 | |
| **Mouse samples** |  |  | |  |  | |  | |  |  |  |
| NORMAL (n=5) | 5 | 0 | | 5 | 0 | | 5 | | 0 | 5 | 0 |
| BBN (n=5) | 0 | 5 | | 0 | 5 | | 0 | | 5 | 0 | 5 |
| BBN + NC (n=5) | 3 | 2 | | 3 | 2 | | 3 | | 2 | 5 | 0 |
| Percentage correctly classified^†^ | 86.7 | | | 86.7 | | | 86.7 | | | 100 | |
| Predicted subclass | **NMIBC** | **MIBC** | | **NMIBC** | **MIBC** | | **NMIBC** | | **MIBC** | **NMIBC** | **MIBC** |
| **Human samples** |  |  | |  |  | |  | |  |  |  |
| NMIBC (n=24) | 22 | 2 | | 22 | 2 | | 22 | | 2 | 20 | 4 |
| MIBC (n=66) | 10 | 56 | | 11 | 55 | | 10 | | 56 | 3 | 63 |
| Percentage correctly classified* | 87 | | | 86 | | | 87 | | | 92 | |
| **Mouse samples** |  |  | |  |  | |  | |  |  |  |
| BBN (n=5) | 0 | 5 | | 0 | 5 | | 0 | | 5 | 0 | 5 |
| Percentage correctly classified^†^ | 100 | | | 100 | | | 100 | | | 100 | |
| Predicted subclass | **NORMAL** | | **NMIBC** | **NORMAL** | | **NMIBC** | **NORMAL** | **NMIBC** | | **NORMAL** | **NMIBC** |
| **Human samples** |  | |  |  | |  |  |  | |  |  |
| NORMAL (n=38) | 36 | | 2 | 37 | | 1 | 37 | 1 | | 37 | 1 |
| NMIBC (n=24) | 0 | | 24 | 0 | | 24 | 0 | 24 | | 0 | 24 |
| Percentage correctly classified* | 96.8 | | | 98.4 | | | 98.4 | | | 98.4 | |
| **Mouse samples** |  | |  |  | |  |  |  | |  |  |
| BBN + NC (n=5) | 4 | | 1 | 4 | | 1 | 4 | 1 | | 4 | 1 |
| Percentage correctly classified^†^ | 80 | | | 80 | | | 80 | | | 80 | |

* Percentage for correct prediction during leave-one-out cross-validation.

^†^ Percentage for histopathologically correct prediction of mouse tissues.

Abbreviations: CCP, compound covariate predictor; LDA, linear discriminator analysis; NEC, nearest centroid; SVM, support vector machines; NMIBC, non-muscle invasive bladder cancer; MIBC, muscle invasive bladder cancer; BBN, N-butyl-N-(4-hydroxybutyl)-nitrosamine; NC, nicotinamide
