# Supplementary material for: Identification of Gene Expression Signature Modulated by Nicotinamide in a Mouse Bladder Cancer Model
Source: PLoS One. 2011 Oct 10;6(10):e26131. doi: 10.1371/journal.pone.0026131 (PMC3189956; doi:10.1371/journal.pone.0026131)
Supplement: Table S3 — Significant gene list (510 genes) applied to gene expression-based prediction methods (Comparison between Normal and MIBC in human). (DOC) [file pone.0026131.s009.doc]

**Table S3. Significant gene list (510 genes) applied to gene expression-based prediction methods (Comparison between Normal and MIBC in human)**

| **No.** | **Gene symbol** | **Entrez ID** | ***Parametric**  ***P*-value** | **^†^Fold-change (Expression ratio of Normal/MIBC)** |
| --- | --- | --- | --- | --- |
| **1** | **CDC20** | 991 | <0.001 | 0.17 |
| **2** | **TOP2A** | 7153 | <0.001 | 0.20 |
| **3** | **UBE2C** | 11065 | <0.001 | 0.23 |
| **4** | **TPX2** | 22974 | <0.001 | 0.24 |
| **5** | **NUSAP1** | 51203 | <0.001 | 0.24 |
| **6** | **CDCA5** | 113130 | <0.001 | 0.24 |
| **7** | **TTK** | 7272 | <0.001 | 0.25 |
| **8** | **CCNB2** | 9133 | <0.001 | 0.26 |
| **9** | **CENPF** | 1063 | <0.001 | 0.27 |
| **10** | **KIF20A** | 10112 | <0.001 | 0.28 |
| **11** | **PRC1** | 9055 | <0.001 | 0.28 |
| **12** | **UHRF1** | 29128 | <0.001 | 0.28 |
| **13** | **CELSR3** | 1951 | <0.001 | 0.29 |
| **14** | **TK1** | 7083 | <0.001 | 0.29 |
| **15** | **TRIP13** | 9319 | <0.001 | 0.29 |
| **16** | **TUBB3** | 10381 | <0.001 | 0.30 |
| **17** | **E2F2** | 1870 | <0.001 | 0.30 |
| **18** | **AURKB** | 9212 | <0.001 | 0.31 |
| **19** | **TACC3** | 10460 | <0.001 | 0.31 |
| **20** | **KIF2C** | 11004 | <0.001 | 0.32 |
| **21** | **TROAP** | 10024 | <0.001 | 0.32 |
| **22** | **TNNT1** | 7138 | <0.001 | 0.33 |
| **23** | **ESM1** | 11082 | <0.001 | 0.33 |
| **24** | **RAD54L** | 8438 | <0.001 | 0.34 |
| **25** | **POLQ** | 10721 | <0.001 | 0.35 |
| **26** | **ASF1B** | 55723 | <0.001 | 0.35 |
| **27** | **C9orf140** | 89958 | <0.001 | 0.36 |
| **28** | **SPAG5** | 10615 | <0.001 | 0.36 |
| **29** | **SPP1** | 6696 | <0.001 | 0.36 |
| **30** | **NMU** | 10874 | <0.001 | 0.36 |
| **31** | **CDCA3** | 83461 | <0.001 | 0.36 |
| **32** | **CALML3** | 810 | <0.001 | 0.37 |
| **33** | **C17orf53** | 78995 | <0.001 | 0.37 |
| **34** | **CDCA8** | 55143 | <0.001 | 0.37 |
| **35** | **MMP1** | 4312 | <0.001 | 0.37 |
| **36** | **RECQL4** | 9401 | <0.001 | 0.38 |
| **37** | **DLG7** | 9787 | <0.001 | 0.38 |
| **38** | **PAQR4** | 124222 | <0.001 | 0.38 |
| **39** | **BUB1B** | 701 | <0.001 | 0.38 |
| **40** | **ANLN** | 54443 | <0.001 | 0.39 |
| **41** | **S100A8** | 6279 | <0.001 | 0.39 |
| **42** | **MELK** | 9833 | <0.001 | 0.40 |
| **43** | **CRH** | 1392 | <0.001 | 0.40 |
| **44** | **HIG2** | 29923 | <0.001 | 0.40 |
| **45** | **FAM64A** | 54478 | <0.001 | 0.40 |
| **46** | **SALL4** | 57167 | <0.001 | 0.40 |
| **47** | **MCM2** | 4171 | <0.001 | 0.41 |
| **48** | **CCNA2** | 890 | <0.001 | 0.41 |
| **49** | **BUB1** | 699 | <0.001 | 0.41 |
| **50** | **PTTG1** | 9232 | <0.001 | 0.42 |
| **51** | **KIAA0101** | 9768 | <0.001 | 0.42 |
| **52** | **CENPA** | 1058 | <0.001 | 0.42 |
| **53** | **TYMS** | 7298 | <0.001 | 0.42 |
| **54** | **CDC2** | 983 | <0.001 | 0.42 |
| **55** | **S100A9** | 6280 | <0.001 | 0.42 |
| **56** | **PBK** | 55872 | <0.001 | 0.43 |
| **57** | **HIST2H2AC** | 8338 | <0.001 | 0.43 |
| **58** | **HIST1H1C** | 3006 | <0.001 | 0.43 |
| **59** | **MMP11** | 4320 | <0.001 | 0.44 |
| **60** | **HES6** | 55502 | <0.001 | 0.44 |
| **61** | **CA9** | 768 | <0.001 | 0.44 |
| **62** | **MTHFD2** | 10797 | <0.001 | 0.45 |
| **63** | **CDKN3** | 1033 | <0.001 | 0.46 |
| **64** | **HMMR** | 3161 | <0.001 | 0.46 |
| **65** | **ETV4** | 2118 | <0.001 | 0.47 |
| **66** | **CHAF1B** | 8208 | <0.001 | 0.47 |
| **67** | **RAD51AP1** | 10635 | <0.001 | 0.47 |
| **68** | **PRSS8** | 5652 | <0.001 | 0.47 |
| **69** | **CENPE** | 1062 | <0.001 | 0.48 |
| **70** | **C20orf46** | 55321 | <0.001 | 0.48 |
| **71** | **GPC2** | 221914 | <0.001 | 0.48 |
| **72** | **PAFAH1B3** | 5050 | <0.001 | 0.48 |
| **73** | **FASN** | 2194 | <0.001 | 0.49 |
| **74** | **HOOK1** | 51361 | <0.001 | 0.49 |
| **75** | **DSCR6** | 53820 | <0.001 | 0.49 |
| **76** | **CLIC3** | 9022 | <0.001 | 0.49 |
| **77** | **PODXL2** | 50512 | <0.001 | 0.50 |
| **78** | **PYCR1** | 5831 | <0.001 | 0.50 |
| **79** | **GGH** | 8836 | <0.001 | 0.50 |
| **80** | **IFI27** | 3429 | <0.001 | 0.50 |
| **81** | **CABP4** | 57010 | <0.001 | 0.51 |
| **82** | **IGSF9** | 57549 | <0.001 | 0.51 |
| **83** | **SOX4** | 6659 | <0.001 | 0.51 |
| **84** | **ENO2** | 2026 | <0.001 | 0.52 |
| **85** | **ATP6V1B1** | 525 | <0.001 | 0.52 |
| **86** | **SLC5A3** | 6526 | <0.001 | 0.52 |
| **87** | **F12** | 2161 | <0.001 | 0.52 |
| **88** | **CDCA7** | 83879 | <0.001 | 0.52 |
| **89** | **SLC7A5** | 8140 | <0.001 | 0.52 |
| **90** | **DSC2** | 1824 | <0.001 | 0.53 |
| **91** | **KPNA2** | 3838 | <0.001 | 0.53 |
| **92** | **SFN** | 2810 | <0.001 | 0.54 |
| **93** | **FGF11** | 2256 | <0.001 | 0.54 |
| **94** | **ANGPTL4** | 51129 | <0.001 | 0.54 |
| **95** | **DHCR24** | 1718 | <0.001 | 0.54 |
| **96** | **SBSN** | 374897 | <0.001 | 0.54 |
| **97** | **APOC1** | 341 | <0.001 | 0.55 |
| **98** | **MESP1** | 55897 | <0.001 | 0.55 |
| **99** | **KIAA1199** | 57214 | <0.001 | 0.55 |
| **100** | **NUP210** | 23225 | <0.001 | 0.55 |
| **101** | **SCD** | 6319 | <0.001 | 0.55 |
| **102** | **CA2** | 760 | <0.001 | 0.55 |
| **103** | **HIST1H2BD** | 3017 | <0.001 | 0.55 |
| **104** | **GPT2** | 84706 | <0.001 | 0.56 |
| **105** | **COL4A1** | 1282 | <0.001 | 0.56 |
| **106** | **RHPN2** | 85415 | <0.001 | 0.56 |
| **107** | **MYB** | 4602 | <0.001 | 0.56 |
| **108** | **HMGB2** | 3148 | <0.001 | 0.56 |
| **109** | **MMP12** | 4321 | <0.001 | 0.57 |
| **110** | **S100A14** | 57402 | <0.001 | 0.57 |
| **111** | **THY1** | 7070 | <0.001 | 0.57 |
| **112** | **SLC6A8** | 6535 | <0.001 | 0.57 |
| **113** | **CDC14B** | 8555 | <0.001 | 0.57 |
| **114** | **SLC29A4** | 222962 | <0.001 | 0.57 |
| **115** | **MYT1** | 4661 | <0.001 | 0.57 |
| **116** | **FSCN1** | 6624 | <0.001 | 0.57 |
| **117** | **RAB3IP** | 117177 | <0.001 | 0.57 |
| **118** | **CNFN** | 84518 | <0.001 | 0.57 |
| **119** | **SOX21** | 11166 | <0.001 | 0.58 |
| **120** | **NDRG1** | 10397 | <0.001 | 0.58 |
| **121** | **GP1BB** | 2812 | <0.001 | 0.58 |
| **122** | **MAGEA10** | 4109 | <0.001 | 0.59 |
| **123** | **HOXC4** | 3221 | <0.001 | 0.59 |
| **124** | **CXCL1** | 2919 | <0.001 | 0.59 |
| **125** | **PVRL4** | 81607 | <0.001 | 0.60 |
| **126** | **BAG4** | 9530 | <0.001 | 0.60 |
| **127** | **TFAP2A** | 7020 | <0.001 | 0.60 |
| **128** | **SOX9** | 6662 | <0.001 | 0.60 |
| **129** | **COCH** | 1690 | <0.001 | 0.61 |
| **130** | **HIC2** | 23119 | <0.001 | 0.61 |
| **131** | **TLE6** | 79816 | <0.001 | 0.61 |
| **132** | **PLOD2** | 5352 | <0.001 | 0.61 |
| **133** | **CKS2** | 1164 | <0.001 | 0.61 |
| **134** | **C10orf10** | 11067 | <0.001 | 0.61 |
| **135** | **FST** | 10468 | <0.001 | 0.62 |
| **136** | **LTB4R** | 1241 | <0.001 | 0.62 |
| **137** | **MCM4** | 4173 | <0.001 | 0.62 |
| **138** | **HMOX1** | 3162 | <0.001 | 0.62 |
| **139** | **ALOX12** | 239 | <0.001 | 0.62 |
| **140** | **FAM112B** | 121355 | <0.001 | 0.62 |
| **141** | **TEAD2** | 8463 | <0.001 | 0.63 |
| **142** | **EPHA1** | 2041 | <0.001 | 0.63 |
| **143** | **STC2** | 8614 | <0.001 | 0.63 |
| **144** | **C20orf94** | 128710 | <0.001 | 0.63 |
| **145** | **MDK** | 4192 | <0.001 | 0.63 |
| **146** | **EPS8L1** | 54869 | <0.001 | 0.63 |
| **147** | **MAD2L1** | 4085 | <0.001 | 0.64 |
| **148** | **LOC642393** | 642393 | <0.001 | 0.65 |
| **149** | **NETO2** | 81831 | <0.001 | 0.65 |
| **150** | **MYH14** | 79784 | <0.001 | 0.65 |
| **151** | **SLC15A1** | 6564 | <0.001 | 0.66 |
| **152** | **HIST2H2BE** | 8349 | <0.001 | 0.68 |
| **153** | **FADS1** | 3992 | <0.001 | 0.68 |
| **154** | **TNFRSF25** | 8718 | <0.001 | 0.69 |
| **155** | **CITED4** | 163732 | <0.001 | 0.69 |
| **156** | **FZD2** | 2535 | <0.001 | 0.69 |
| **157** | **FCHO1** | 23149 | <0.001 | 0.69 |
| **158** | **TBX1** | 6899 | <0.001 | 0.71 |
| **159** | **RHPN1** | 114822 | <0.001 | 0.71 |
| **160** | **APOB48R** | 55911 | <0.001 | 1.36 |
| **161** | **NOXA1** | 10811 | <0.001 | 1.39 |
| **162** | **TMEM14A** | 28978 | <0.001 | 1.40 |
| **163** | **SMAD6** | 4091 | <0.001 | 1.45 |
| **164** | **MGST1** | 4257 | <0.001 | 1.46 |
| **165** | **CD2AP** | 23607 | <0.001 | 1.48 |
| **166** | **UNC5B** | 219699 | <0.001 | 1.50 |
| **167** | **IFI16** | 3428 | <0.001 | 1.50 |
| **168** | **DC2** | 58505 | <0.001 | 1.50 |
| **169** | **SLC16A5** | 9121 | <0.001 | 1.51 |
| **170** | **TEPP** | 374739 | <0.001 | 1.51 |
| **171** | **DSG2** | 1829 | <0.001 | 1.51 |
| **172** | **CPVL** | 54504 | <0.001 | 1.51 |
| **173** | **DNALI1** | 7802 | <0.001 | 1.52 |
| **174** | **NXN** | 64359 | <0.001 | 1.53 |
| **175** | **PLCD3** | 113026 | <0.001 | 1.53 |
| **176** | **CAV2** | 858 | <0.001 | 1.54 |
| **177** | **MTAP** | 4507 | <0.001 | 1.54 |
| **178** | **ALDH1A2** | 8854 | <0.001 | 1.55 |
| **179** | **SERPING1** | 710 | <0.001 | 1.55 |
| **180** | **HLA-DMA** | 3108 | <0.001 | 1.57 |
| **181** | **C10orf58** | 84293 | <0.001 | 1.57 |
| **182** | **HCST** | 10870 | <0.001 | 1.57 |
| **183** | **LXN** | 56925 | <0.001 | 1.58 |
| **184** | **SERPINB11** | 89778 | <0.001 | 1.58 |
| **185** | **ITGA2** | 3673 | <0.001 | 1.58 |
| **186** | **PLS3** | 5358 | <0.001 | 1.60 |
| **187** | **C9orf19** | 152007 | <0.001 | 1.60 |
| **188** | **RUTBC2** | 129049 | <0.001 | 1.61 |
| **189** | **CD37** | 951 | <0.001 | 1.61 |
| **190** | **PALM** | 5064 | <0.001 | 1.61 |
| **191** | **SLIT3** | 6586 | <0.001 | 1.61 |
| **192** | **RRAS** | 6237 | <0.001 | 1.62 |
| **193** | **COL15A1** | 1306 | <0.001 | 1.62 |
| **194** | **ZNF135** | 7694 | <0.001 | 1.62 |
| **195** | **NELL2** | 4753 | <0.001 | 1.62 |
| **196** | **PLS1** | 5357 | <0.001 | 1.63 |
| **197** | **AP3S1** | 1176 | <0.001 | 1.63 |
| **198** | **DKFZP564J102** | 25854 | <0.001 | 1.63 |
| **199** | **SLC24A3** | 57419 | <0.001 | 1.63 |
| **200** | **ZFP36L1** | 677 | <0.001 | 1.64 |
| **201** | **COL5A1** | 1289 | <0.001 | 1.64 |
| **202** | **ITPR1** | 3708 | <0.001 | 1.64 |
| **203** | **RPLP0** | 6175 | <0.001 | 1.64 |
| **204** | **POU2AF1** | 5450 | <0.001 | 1.65 |
| **205** | **SOX15** | 6665 | <0.001 | 1.65 |
| **206** | **CHPT1** | 56994 | <0.001 | 1.66 |
| **207** | **CD44** | 960 | <0.001 | 1.66 |
| **208** | **FLJ21438** | 64926 | <0.001 | 1.66 |
| **209** | **EHF** | 26298 | <0.001 | 1.66 |
| **210** | **PRDM8** | 56978 | <0.001 | 1.66 |
| **211** | **TIMP2** | 7077 | <0.001 | 1.66 |
| **212** | **KIT** | 3815 | <0.001 | 1.67 |
| **213** | **EOMES** | 8320 | <0.001 | 1.67 |
| **214** | **NT5E** | 4907 | <0.001 | 1.67 |
| **215** | **CCL5** | 6352 | <0.001 | 1.67 |
| **216** | **TGFBI** | 7045 | <0.001 | 1.67 |
| **217** | **MS4A6A** | 64231 | <0.001 | 1.68 |
| **218** | **BMP4** | 652 | <0.001 | 1.68 |
| **219** | **RPL22** | 6146 | <0.001 | 1.68 |
| **220** | **TGFB1I1** | 7041 | <0.001 | 1.68 |
| **221** | **C10orf33** | 84795 | <0.001 | 1.69 |
| **222** | **CAP2** | 10486 | <0.001 | 1.69 |
| **223** | **PDE5A** | 8654 | <0.001 | 1.69 |
| **224** | **IGFBP7** | 3490 | <0.001 | 1.70 |
| **225** | **BMP7** | 655 | <0.001 | 1.70 |
| **226** | **F2RL1** | 2150 | <0.001 | 1.71 |
| **227** | **COL6A3** | 1293 | <0.001 | 1.71 |
| **228** | **GMFG** | 9535 | <0.001 | 1.71 |
| **229** | **RHOB** | 388 | <0.001 | 1.72 |
| **230** | **C10orf38** | 221061 | <0.001 | 1.72 |
| **231** | **RAPGEF3** | 10411 | <0.001 | 1.74 |
| **232** | **CD6** | 923 | <0.001 | 1.74 |
| **233** | **CRYZ** | 1429 | <0.001 | 1.74 |
| **234** | **PTPLA** | 9200 | <0.001 | 1.75 |
| **235** | **GPR126** | 57211 | <0.001 | 1.75 |
| **236** | **ARMCX2** | 9823 | <0.001 | 1.75 |
| **237** | **ARMCX1** | 51309 | <0.001 | 1.75 |
| **238** | **NFIX** | 4784 | <0.001 | 1.75 |
| **239** | **GSPT2** | 23708 | <0.001 | 1.76 |
| **240** | **PCOLCE2** | 26577 | <0.001 | 1.76 |
| **241** | **EBI2** | 1880 | <0.001 | 1.76 |
| **242** | **QPCT** | 25797 | <0.001 | 1.77 |
| **243** | **RPL7** | 6129 | <0.001 | 1.77 |
| **244** | **PTRF** | 284119 | <0.001 | 1.77 |
| **245** | **CSF1R** | 1436 | <0.001 | 1.77 |
| **246** | **HLA-DMB** | 3109 | <0.001 | 1.77 |
| **247** | **LEPREL1** | 55214 | <0.001 | 1.78 |
| **248** | **FOXF2** | 2295 | <0.001 | 1.78 |
| **249** | **HOXA9** | 3205 | <0.001 | 1.78 |
| **250** | **GATA2** | 2624 | <0.001 | 1.78 |
| **251** | **RARRES2** | 5919 | <0.001 | 1.78 |
| **252** | **GPNMB** | 10457 | <0.001 | 1.79 |
| **253** | **OSBPL10** | 114884 | <0.001 | 1.80 |
| **254** | **STAB1** | 23166 | <0.001 | 1.80 |
| **255** | **GSTM5** | 2949 | <0.001 | 1.81 |
| **256** | **DOCK2** | 1794 | <0.001 | 1.81 |
| **257** | **PPP1R14A** | 94274 | <0.001 | 1.81 |
| **258** | **LOC388743** | 388743 | <0.001 | 1.82 |
| **259** | **GCLC** | 2729 | <0.001 | 1.82 |
| **260** | **D4S234E** | 27065 | <0.001 | 1.82 |
| **261** | **DPYSL2** | 1808 | <0.001 | 1.82 |
| **262** | **PRICKLE1** | 144165 | <0.001 | 1.82 |
| **263** | **C9orf152** | 401546 | <0.001 | 1.83 |
| **264** | **DKK3** | 27122 | <0.001 | 1.83 |
| **265** | **CKB** | 1152 | <0.001 | 1.83 |
| **266** | **B2M** | 567 | <0.001 | 1.83 |
| **267** | **ALDH4A1** | 8659 | <0.001 | 1.83 |
| **268** | **MLPH** | 79083 | <0.001 | 1.83 |
| **269** | **KLF2** | 10365 | <0.001 | 1.83 |
| **270** | **CAV1** | 857 | <0.001 | 1.85 |
| **271** | **WEE1** | 7465 | <0.001 | 1.85 |
| **272** | **TRIM2** | 23321 | <0.001 | 1.85 |
| **273** | **BCAS1** | 8537 | <0.001 | 1.85 |
| **274** | **IL7R** | 3575 | <0.001 | 1.85 |
| **275** | **KIAA0746** | 23231 | <0.001 | 1.86 |
| **276** | **C8orf42** | 157695 | <0.001 | 1.86 |
| **277** | **C9orf95** | 54981 | <0.001 | 1.86 |
| **278** | **LITAF** | 9516 | <0.001 | 1.87 |
| **279** | **LOC63928** | 63928 | <0.001 | 1.87 |
| **280** | **HLA-DOA** | 3111 | <0.001 | 1.87 |
| **281** | **SERPINB5** | 5268 | <0.001 | 1.88 |
| **282** | **CENTB1** | 9744 | <0.001 | 1.88 |
| **283** | **RASD1** | 51655 | <0.001 | 1.88 |
| **284** | **PLSCR4** | 57088 | <0.001 | 1.88 |
| **285** | **NFIA** | 4774 | <0.001 | 1.89 |
| **286** | **LPXN** | 9404 | <0.001 | 1.89 |
| **287** | **ANKRD25** | 25959 | <0.001 | 1.89 |
| **288** | **PTPN13** | 5783 | <0.001 | 1.90 |
| **289** | **PLEKHC1** | 10979 | <0.001 | 1.90 |
| **290** | **GPX3** | 2878 | <0.001 | 1.91 |
| **291** | **ADHFE1** | 137872 | <0.001 | 1.91 |
| **292** | **STEAP2** | 261729 | <0.001 | 1.92 |
| **293** | **MGC33846** | 220382 | <0.001 | 1.92 |
| **294** | **C10orf56** | 219654 | <0.001 | 1.93 |
| **295** | **GAS6** | 2621 | <0.001 | 1.93 |
| **296** | **EDNRA** | 1909 | <0.001 | 1.93 |
| **297** | **SLC39A6** | 25800 | <0.001 | 1.94 |
| **298** | **EMP3** | 2014 | <0.001 | 1.94 |
| **299** | **PRDX3** | 10935 | <0.001 | 1.94 |
| **300** | **ROR2** | 4920 | <0.001 | 1.95 |
| **301** | **FBP1** | 2203 | <0.001 | 1.95 |
| **302** | **C6orf105** | 84830 | <0.001 | 1.95 |
| **303** | **HOXD1** | 3231 | <0.001 | 1.95 |
| **304** | **CD48** | 962 | <0.001 | 1.95 |
| **305** | **MATN2** | 4147 | <0.001 | 1.95 |
| **306** | **C1S** | 716 | <0.001 | 1.96 |
| **307** | **TNC** | 3371 | <0.001 | 1.96 |
| **308** | **SGCE** | 8910 | <0.001 | 1.96 |
| **309** | **CYP3A5** | 1577 | <0.001 | 1.97 |
| **310** | **RNASE4** | 6038 | <0.001 | 1.97 |
| **311** | **EGR1** | 1958 | <0.001 | 1.97 |
| **312** | **CYGB** | 114757 | <0.001 | 1.98 |
| **313** | **SRPX2** | 27286 | <0.001 | 1.98 |
| **314** | **CX3CL1** | 6376 | <0.001 | 1.98 |
| **315** | **AEBP1** | 165 | <0.001 | 1.98 |
| **316** | **AQP1** | 358 | <0.001 | 1.99 |
| **317** | **PROM1** | 8842 | <0.001 | 2.00 |
| **318** | **CSRP1** | 1465 | <0.001 | 2.00 |
| **319** | **PMP22** | 5376 | <0.001 | 2.00 |
| **320** | **ALDH7A1** | 501 | <0.001 | 2.00 |
| **321** | **CAPNS2** | 84290 | <0.001 | 2.01 |
| **322** | **GJA1** | 2697 | <0.001 | 2.02 |
| **323** | **IGFBP6** | 3489 | <0.001 | 2.02 |
| **324** | **MAOB** | 4129 | <0.001 | 2.02 |
| **325** | **FXYD6** | 53826 | <0.001 | 2.03 |
| **326** | **TMPRSS4** | 56649 | <0.001 | 2.03 |
| **327** | **SYNPO2** | 171024 | <0.001 | 2.04 |
| **328** | **SORL1** | 6653 | <0.001 | 2.04 |
| **329** | **APOD** | 347 | <0.001 | 2.05 |
| **330** | **ENTPD3** | 956 | <0.001 | 2.05 |
| **331** | **THBS2** | 7058 | <0.001 | 2.05 |
| **332** | **HOXA5** | 3202 | <0.001 | 2.06 |
| **333** | **CNTN3** | 5067 | <0.001 | 2.06 |
| **334** | **RPS4X** | 6191 | <0.001 | 2.06 |
| **335** | **HLA-F** | 3134 | <0.001 | 2.07 |
| **336** | **PLA2G10** | 8399 | <0.001 | 2.08 |
| **337** | **UST** | 10090 | <0.001 | 2.08 |
| **338** | **COLEC12** | 81035 | <0.001 | 2.08 |
| **339** | **COL6A2** | 1292 | <0.001 | 2.09 |
| **340** | **LOC92196** | 92196 | <0.001 | 2.09 |
| **341** | **PELI2** | 57161 | <0.001 | 2.10 |
| **342** | **GPR124** | 25960 | <0.001 | 2.11 |
| **343** | **ALOX5AP** | 241 | <0.001 | 2.11 |
| **344** | **SLIT2** | 9353 | <0.001 | 2.11 |
| **345** | **C6orf189** | 221303 | <0.001 | 2.12 |
| **346** | **TSPYL1** | 7259 | <0.001 | 2.12 |
| **347** | **CCL19** | 6363 | <0.001 | 2.12 |
| **348** | **SPARC** | 6678 | <0.001 | 2.12 |
| **349** | **PAM** | 5066 | <0.001 | 2.13 |
| **350** | **EGR2** | 1959 | <0.001 | 2.13 |
| **351** | **TGFB3** | 7043 | <0.001 | 2.14 |
| **352** | **UPK1B** | 7348 | <0.001 | 2.14 |
| **353** | **RNF150** | 57484 | <0.001 | 2.14 |
| **354** | **ATOH8** | 84913 | <0.001 | 2.14 |
| **355** | **CXCL12** | 6387 | <0.001 | 2.15 |
| **356** | **CD2** | 914 | <0.001 | 2.16 |
| **357** | **GHR** | 2690 | <0.001 | 2.17 |
| **358** | **PTGDS** | 5730 | <0.001 | 2.18 |
| **359** | **DPYSL3** | 1809 | <0.001 | 2.18 |
| **360** | **EPB41L3** | 23136 | <0.001 | 2.18 |
| **361** | **PDK4** | 5166 | <0.001 | 2.19 |
| **362** | **LHFP** | 10186 | <0.001 | 2.19 |
| **363** | **EMILIN1** | 11117 | <0.001 | 2.20 |
| **364** | **GNG10** | 2790 | <0.001 | 2.20 |
| **365** | **DKK1** | 22943 | <0.001 | 2.20 |
| **366** | **TPM2** | 7169 | <0.001 | 2.21 |
| **367** | **ITM2C** | 81618 | <0.001 | 2.21 |
| **368** | **ZBTB16** | 7704 | <0.001 | 2.22 |
| **369** | **TPM1** | 7168 | <0.001 | 2.23 |
| **370** | **DUSP2** | 1844 | <0.001 | 2.23 |
| **371** | **C21orf63** | 59271 | <0.001 | 2.24 |
| **372** | **TGFBR3** | 7049 | <0.001 | 2.24 |
| **373** | **SERPINF1** | 5176 | <0.001 | 2.24 |
| **374** | **VIM** | 7431 | <0.001 | 2.25 |
| **375** | **RGS1** | 5996 | <0.001 | 2.25 |
| **376** | **HOXA13** | 3209 | <0.001 | 2.26 |
| **377** | **COL1A2** | 1278 | <0.001 | 2.26 |
| **378** | **OLFML3** | 56944 | <0.001 | 2.26 |
| **379** | **CDC42EP5** | 148170 | <0.001 | 2.26 |
| **380** | **BTF3** | 689 | <0.001 | 2.27 |
| **381** | **WFDC1** | 58189 | <0.001 | 2.28 |
| **382** | **GNG11** | 2791 | <0.001 | 2.28 |
| **383** | **RBPMS2** | 348093 | <0.001 | 2.31 |
| **384** | **COL6A1** | 1291 | <0.001 | 2.31 |
| **385** | **SH3BGRL** | 6451 | <0.001 | 2.31 |
| **386** | **HSD17B2** | 3294 | <0.001 | 2.32 |
| **387** | **RGS2** | 5997 | <0.001 | 2.33 |
| **388** | **DKFZP586H2123** | 25891 | <0.001 | 2.34 |
| **389** | **C2orf32** | 25927 | <0.001 | 2.34 |
| **390** | **NR2F1** | 7025 | <0.001 | 2.35 |
| **391** | **ALDH2** | 217 | <0.001 | 2.36 |
| **392** | **PRICKLE2** | 166336 | <0.001 | 2.37 |
| **393** | **C8orf4** | 56892 | <0.001 | 2.38 |
| **394** | **PI16** | 221476 | <0.001 | 2.38 |
| **395** | **AXIN2** | 8313 | <0.001 | 2.39 |
| **396** | **TMOD1** | 7111 | <0.001 | 2.39 |
| **397** | **IDH1** | 3417 | <0.001 | 2.39 |
| **398** | **A2M** | 2 | <0.001 | 2.39 |
| **399** | **CYBRD1** | 79901 | <0.001 | 2.39 |
| **400** | **TAGLN** | 6876 | <0.001 | 2.39 |
| **401** | **ANXA10** | 11199 | <0.001 | 2.40 |
| **402** | **ANTXR2** | 118429 | <0.001 | 2.40 |
| **403** | **BIN1** | 274 | <0.001 | 2.41 |
| **404** | **FCER1A** | 2205 | <0.001 | 2.41 |
| **405** | **MYL9** | 10398 | <0.001 | 2.41 |
| **406** | **FGL2** | 10875 | <0.001 | 2.41 |
| **407** | **LGALS3** | 3958 | <0.001 | 2.41 |
| **408** | **LMOD1** | 25802 | <0.001 | 2.43 |
| **409** | **ALDH1A3** | 220 | <0.001 | 2.43 |
| **410** | **CACNA1H** | 8912 | <0.001 | 2.43 |
| **411** | **HLA-DRB1** | 3123 | <0.001 | 2.44 |
| **412** | **P2RX1** | 5023 | <0.001 | 2.44 |
| **413** | **RGS5** | 8490 | <0.001 | 2.44 |
| **414** | **FOS** | 2353 | <0.001 | 2.45 |
| **415** | **BTG2** | 7832 | <0.001 | 2.46 |
| **416** | **HSPB8** | 26353 | <0.001 | 2.46 |
| **417** | **TGFBR2** | 7048 | <0.001 | 2.47 |
| **418** | **JAM3** | 83700 | <0.001 | 2.47 |
| **419** | **CASQ2** | 845 | <0.001 | 2.47 |
| **420** | **GATM** | 2628 | <0.001 | 2.48 |
| **421** | **COL4A5** | 1287 | <0.001 | 2.50 |
| **422** | **PLA2G4A** | 5321 | <0.001 | 2.51 |
| **423** | **CYP1B1** | 1545 | <0.001 | 2.52 |
| **424** | **ACACB** | 32 | <0.001 | 2.52 |
| **425** | **PDGFD** | 80310 | <0.001 | 2.53 |
| **426** | **PLAT** | 5327 | <0.001 | 2.54 |
| **427** | **ACOX2** | 8309 | <0.001 | 2.56 |
| **428** | **TESC** | 54997 | <0.001 | 2.57 |
| **429** | **ITM2A** | 9452 | <0.001 | 2.57 |
| **430** | **PDE7B** | 27115 | <0.001 | 2.57 |
| **431** | **NDN** | 4692 | <0.001 | 2.59 |
| **432** | **CYP27A1** | 1593 | <0.001 | 2.59 |
| **433** | **CDH11** | 1009 | <0.001 | 2.60 |
| **434** | **RASL12** | 51285 | <0.001 | 2.60 |
| **435** | **UPK3A** | 7380 | <0.001 | 2.61 |
| **436** | **ADAMTS1** | 9510 | <0.001 | 2.61 |
| **437** | **C3** | 718 | <0.001 | 2.64 |
| **438** | **ABCC4** | 10257 | <0.001 | 2.64 |
| **439** | **DIXDC1** | 85458 | <0.001 | 2.64 |
| **440** | **LTB4DH** | 22949 | <0.001 | 2.65 |
| **441** | **DPT** | 1805 | <0.001 | 2.67 |
| **442** | **LPPR4** | 9890 | <0.001 | 2.68 |
| **443** | **PTGIS** | 5740 | <0.001 | 2.69 |
| **444** | **SYT8** | 90019 | <0.001 | 2.69 |
| **445** | **KCNMB1** | 3779 | <0.001 | 2.71 |
| **446** | **MAMDC2** | 256691 | <0.001 | 2.73 |
| **447** | **CRYAB** | 1410 | <0.001 | 2.74 |
| **448** | **TCF21** | 6943 | <0.001 | 2.76 |
| **449** | **UPK1A** | 11045 | <0.001 | 2.76 |
| **450** | **MMP7** | 4316 | <0.001 | 2.76 |
| **451** | **LGALS4** | 3960 | <0.001 | 2.78 |
| **452** | **SDPR** | 8436 | <0.001 | 2.78 |
| **453** | **ACTA2** | 59 | <0.001 | 2.78 |
| **454** | **ADAMTS8** | 11095 | <0.001 | 2.80 |
| **455** | **PEG3** | 5178 | <0.001 | 2.82 |
| **456** | **TNNT3** | 7140 | <0.001 | 2.82 |
| **457** | **ITGA8** | 8516 | <0.001 | 2.84 |
| **458** | **ALDH1A1** | 216 | <0.001 | 2.84 |
| **459** | **SCN11A** | 11280 | <0.001 | 2.85 |
| **460** | **LTBP4** | 8425 | <0.001 | 2.86 |
| **461** | **HLA-DRA** | 3122 | <0.001 | 2.90 |
| **462** | **COX7A1** | 1346 | <0.001 | 2.91 |
| **463** | **DMN** | 23336 | <0.001 | 2.92 |
| **464** | **ATP1A2** | 477 | <0.001 | 2.92 |
| **465** | **ENPP2** | 5168 | <0.001 | 2.93 |
| **466** | **FGF9** | 2254 | <0.001 | 2.93 |
| **467** | **HLA-DQA1** | 3117 | <0.001 | 2.94 |
| **468** | **PLAC9** | 219348 | <0.001 | 2.94 |
| **469** | **SORBS1** | 10580 | <0.001 | 2.96 |
| **470** | **SCUBE2** | 57758 | <0.001 | 3.02 |
| **471** | **KIAA0367** | 23273 | <0.001 | 3.02 |
| **472** | **SMOC2** | 64094 | <0.001 | 3.02 |
| **473** | **SH3GL2** | 6456 | <0.001 | 3.04 |
| **474** | **MOXD1** | 26002 | <0.001 | 3.05 |
| **475** | **MYLK** | 4638 | <0.001 | 3.05 |
| **476** | **FLJ21986** | 79974 | <0.001 | 3.08 |
| **477** | **IGFBP5** | 3488 | <0.001 | 3.09 |
| **478** | **COL16A1** | 1307 | <0.001 | 3.10 |
| **479** | **SLC14A1** | 6563 | <0.001 | 3.12 |
| **480** | **FOXF1** | 2294 | <0.001 | 3.22 |
| **481** | **PDGFRA** | 5156 | <0.001 | 3.28 |
| **482** | **MRGPRF** | 219928 | <0.001 | 3.28 |
| **483** | **IGFBP2** | 3485 | <0.001 | 3.31 |
| **484** | **DKFZP564O0823** | 25849 | <0.001 | 3.32 |
| **485** | **PCP4** | 5121 | <0.001 | 3.37 |
| **486** | **LAMC3** | 10319 | <0.001 | 3.41 |
| **487** | **FHL1** | 2273 | <0.001 | 3.43 |
| **488** | **SEPP1** | 6414 | <0.001 | 3.43 |
| **489** | **ISL1** | 3670 | <0.001 | 3.44 |
| **490** | **DCN** | 1634 | <0.001 | 3.45 |
| **491** | **PGM5** | 5239 | <0.001 | 3.46 |
| **492** | **LUM** | 4060 | <0.001 | 3.50 |
| **493** | **PIGR** | 5284 | <0.001 | 3.56 |
| **494** | **WNT5A** | 7474 | <0.001 | 3.57 |
| **495** | **CLIC6** | 54102 | <0.001 | 3.60 |
| **496** | **ABCA8** | 10351 | <0.001 | 3.86 |
| **497** | **PTGS1** | 5742 | <0.001 | 3.88 |
| **498** | **IGJ** | 3512 | <0.001 | 3.90 |
| **499** | **SPARCL1** | 8404 | <0.001 | 3.98 |
| **500** | **CRTAC1** | 55118 | <0.001 | 4.02 |
| **501** | **CCND2** | 894 | <0.001 | 4.07 |
| **502** | **DES** | 1674 | <0.001 | 4.08 |
| **503** | **FAM107A** | 11170 | <0.001 | 4.15 |
| **504** | **SPON1** | 10418 | <0.001 | 4.28 |
| **505** | **CNN1** | 1264 | <0.001 | 4.56 |
| **506** | **SRPX** | 8406 | <0.001 | 4.62 |
| **507** | **CLCA4** | 22802 | <0.001 | 4.62 |
| **508** | **FABP4** | 2167 | <0.001 | 5.72 |
| **509** | **MYH11** | 4629 | <0.001 | 5.95 |
| **510** | **MFAP4** | 4239 | <0.001 | 6.29 |

* The parametic *P*-values were obtained by two sample t-tests.

^†^ Genes were sorted by fold change values.

Abbreviations: MIBC, muscle invasive bladder cancer
