# Supplementary material for: Identification of Gene Expression Signature Modulated by Nicotinamide in a Mouse Bladder Cancer Model
Source: PLoS One. 2011 Oct 10;6(10):e26131. doi: 10.1371/journal.pone.0026131 (PMC3189956; doi:10.1371/journal.pone.0026131)
Supplement: Table S4 — Significant gene list (417 genes) applied to gene expression-based prediction methods (Comparison between NMIBC and MIBC in human). (DOC) [file pone.0026131.s010.doc]

**Table S4. Significant gene list (417 genes) applied to gene expression-based prediction methods (Comparison between NMIBC** and MIBC in human)

| **No.** | **Gene symbol** | **Entrez ID** | ***Parametric**  ***P*-value** | **^†^Fold-change (Expression ratio of NMIBC/MIBC)** |
| --- | --- | --- | --- | --- |
| **1** | **S100A8** | 6279 | <0.001 | 0.26 |
| **2** | **MT2A** | 4502 | <0.001 | 0.32 |
| **3** | **CTGF** | 1490 | <0.001 | 0.33 |
| **4** | **ATF3** | 467 | <0.001 | 0.35 |
| **5** | **SLC2A3** | 6515 | <0.001 | 0.36 |
| **6** | **SERPINE2** | 5270 | <0.001 | 0.40 |
| **7** | **FOSB** | 2354 | <0.001 | 0.40 |
| **8** | **AEBP1** | 165 | <0.001 | 0.40 |
| **9** | **RBP1** | 5947 | <0.001 | 0.40 |
| **10** | **CTHRC1** | 115908 | <0.001 | 0.41 |
| **11** | **CYR61** | 3491 | <0.001 | 0.41 |
| **12** | **NNMT** | 4837 | <0.001 | 0.41 |
| **13** | **RGS1** | 5996 | <0.001 | 0.41 |
| **14** | **CNN1** | 1264 | <0.001 | 0.42 |
| **15** | **UCHL1** | 7345 | <0.001 | 0.43 |
| **16** | **TAGLN** | 6876 | <0.001 | 0.43 |
| **17** | **GFPT2** | 9945 | <0.001 | 0.43 |
| **18** | **TUBB3** | 10381 | <0.001 | 0.44 |
| **19** | **COL1A1** | 1277 | <0.001 | 0.44 |
| **20** | **MTHFD2** | 10797 | <0.001 | 0.44 |
| **21** | **PTRF** | 284119 | <0.001 | 0.44 |
| **22** | **TEAD2** | 8463 | <0.001 | 0.45 |
| **23** | **EGR1** | 1958 | <0.001 | 0.45 |
| **24** | **IMPA2** | 3613 | <0.001 | 0.46 |
| **25** | **PCP4** | 5121 | <0.001 | 0.46 |
| **26** | **SFRP1** | 6422 | <0.001 | 0.46 |
| **27** | **ITGA5** | 3678 | <0.001 | 0.46 |
| **28** | **DES** | 1674 | <0.001 | 0.46 |
| **29** | **CCL2** | 6347 | <0.001 | 0.46 |
| **30** | **C9orf19** | 152007 | <0.001 | 0.46 |
| **31** | **COL5A2** | 1290 | <0.001 | 0.47 |
| **32** | **NMU** | 10874 | <0.001 | 0.47 |
| **33** | **RBPMS2** | 348093 | <0.001 | 0.47 |
| **34** | **RGS2** | 5997 | <0.001 | 0.47 |
| **35** | **ADAMTS4** | 9507 | <0.001 | 0.47 |
| **36** | **MYH11** | 4629 | <0.001 | 0.47 |
| **37** | **TXNRD1** | 7296 | <0.001 | 0.48 |
| **38** | **CRIP1** | 1396 | <0.001 | 0.48 |
| **39** | **KIAA0367** | 23273 | <0.001 | 0.48 |
| **40** | **ANXA1** | 301 | <0.001 | 0.48 |
| **41** | **GALNAC4S-6ST** | 51363 | <0.001 | 0.48 |
| **42** | **IER3** | 8870 | <0.001 | 0.48 |
| **43** | **CRYAB** | 1410 | <0.001 | 0.48 |
| **44** | **S100A9** | 6280 | <0.001 | 0.48 |
| **45** | **TNC** | 3371 | <0.001 | 0.49 |
| **46** | **DUSP1** | 1843 | <0.001 | 0.49 |
| **47** | **PMP22** | 5376 | <0.001 | 0.49 |
| **48** | **PHLDA1** | 22822 | <0.001 | 0.49 |
| **49** | **SNCAIP** | 9627 | <0.001 | 0.49 |
| **50** | **ACTN1** | 87 | <0.001 | 0.49 |
| **51** | **COL3A1** | 1281 | <0.001 | 0.49 |
| **52** | **COL6A3** | 1293 | <0.001 | 0.49 |
| **53** | **TM4SF1** | 4071 | <0.001 | 0.50 |
| **54** | **SGCE** | 8910 | <0.001 | 0.50 |
| **55** | **TIMP2** | 7077 | <0.001 | 0.50 |
| **56** | **SFRP2** | 6423 | <0.001 | 0.50 |
| **57** | **CDCA5** | 113130 | <0.001 | 0.50 |
| **58** | **PGM5** | 5239 | <0.001 | 0.50 |
| **59** | **DPYSL3** | 1809 | <0.001 | 0.51 |
| **60** | **LGALS1** | 3956 | <0.001 | 0.51 |
| **61** | **COL5A1** | 1289 | <0.001 | 0.51 |
| **62** | **TPM2** | 7169 | <0.001 | 0.51 |
| **63** | **SULF1** | 23213 | <0.001 | 0.51 |
| **64** | **GP1BB** | 2812 | <0.001 | 0.51 |
| **65** | **EMP1** | 2012 | <0.001 | 0.51 |
| **66** | **LDLR** | 3949 | <0.001 | 0.51 |
| **67** | **DMN** | 23336 | <0.001 | 0.52 |
| **68** | **MMP3** | 4314 | <0.001 | 0.52 |
| **69** | **TOP2A** | 7153 | <0.001 | 0.52 |
| **70** | **C1QA** | 712 | <0.001 | 0.52 |
| **71** | **LMCD1** | 29995 | <0.001 | 0.52 |
| **72** | **RGS11** | 8786 | <0.001 | 0.52 |
| **73** | **S100A10** | 6281 | <0.001 | 0.52 |
| **74** | **C1S** | 716 | <0.001 | 0.52 |
| **75** | **LUM** | 4060 | <0.001 | 0.52 |
| **76** | **SMOC2** | 64094 | <0.001 | 0.52 |
| **77** | **TPM1** | 7168 | <0.001 | 0.52 |
| **78** | **ACTA2** | 59 | <0.001 | 0.53 |
| **79** | **CDC20** | 991 | <0.001 | 0.53 |
| **80** | **TGM2** | 7052 | <0.001 | 0.53 |
| **81** | **SALL4** | 57167 | <0.001 | 0.53 |
| **82** | **BIN1** | 274 | <0.001 | 0.53 |
| **83** | **FXYD6** | 53826 | <0.001 | 0.53 |
| **84** | **CD14** | 929 | <0.001 | 0.53 |
| **85** | **EMP3** | 2014 | <0.001 | 0.54 |
| **86** | **TPX2** | 22974 | <0.001 | 0.54 |
| **87** | **CCNB2** | 9133 | <0.001 | 0.54 |
| **88** | **KCNMB1** | 3779 | <0.001 | 0.54 |
| **89** | **APOE** | 348 | <0.001 | 0.54 |
| **90** | **PLEKHC1** | 10979 | <0.001 | 0.54 |
| **91** | **PDK4** | 5166 | <0.001 | 0.55 |
| **92** | **C5orf13** | 9315 | <0.001 | 0.55 |
| **93** | **MYOM1** | 8736 | <0.001 | 0.55 |
| **94** | **FHL1** | 2273 | <0.001 | 0.55 |
| **95** | **VIM** | 7431 | <0.001 | 0.55 |
| **96** | **SLC29A4** | 222962 | <0.001 | 0.56 |
| **97** | **CXCR4** | 7852 | <0.001 | 0.56 |
| **98** | **THY1** | 7070 | <0.001 | 0.56 |
| **99** | **CENPF** | 1063 | <0.001 | 0.56 |
| **100** | **EGR2** | 1959 | <0.001 | 0.56 |
| **101** | **FADS1** | 3992 | <0.001 | 0.56 |
| **102** | **UBE2C** | 11065 | <0.001 | 0.56 |
| **103** | **SGK** | 6446 | <0.001 | 0.56 |
| **104** | **COL6A1** | 1291 | <0.001 | 0.56 |
| **105** | **ALDH1A1** | 216 | <0.001 | 0.56 |
| **106** | **COL4A1** | 1282 | <0.001 | 0.56 |
| **107** | **DPYSL2** | 1808 | <0.001 | 0.56 |
| **108** | **TRIP13** | 9319 | <0.001 | 0.56 |
| **109** | **C1QB** | 713 | <0.001 | 0.56 |
| **110** | **PRKCDBP** | 112464 | <0.001 | 0.56 |
| **111** | **DHCR24** | 1718 | <0.001 | 0.57 |
| **112** | **TGFBI** | 7045 | <0.001 | 0.57 |
| **113** | **FAM46B** | 115572 | <0.001 | 0.57 |
| **114** | **HMGB2** | 3148 | <0.001 | 0.57 |
| **115** | **PLEK** | 5341 | <0.001 | 0.57 |
| **116** | **TACC3** | 10460 | <0.001 | 0.57 |
| **117** | **TNFAIP3** | 7128 | <0.001 | 0.57 |
| **118** | **P2RX1** | 5023 | <0.001 | 0.57 |
| **119** | **DSC2** | 1824 | <0.001 | 0.57 |
| **120** | **COL16A1** | 1307 | <0.001 | 0.57 |
| **121** | **CENPA** | 1058 | <0.001 | 0.57 |
| **122** | **KIF1A** | 547 | <0.001 | 0.57 |
| **123** | **MRGPRF** | 219928 | <0.001 | 0.57 |
| **124** | **COCH** | 1690 | <0.001 | 0.57 |
| **125** | **CLIC4** | 25932 | <0.001 | 0.57 |
| **126** | **TK1** | 7083 | <0.001 | 0.58 |
| **127** | **MMP11** | 4320 | <0.001 | 0.58 |
| **128** | **C9orf58** | 83543 | <0.001 | 0.58 |
| **129** | **PRC1** | 9055 | <0.001 | 0.58 |
| **130** | **IL6** | 3569 | <0.001 | 0.58 |
| **131** | **NUSAP1** | 51203 | <0.001 | 0.58 |
| **132** | **CASQ2** | 845 | <0.001 | 0.58 |
| **133** | **CYBRD1** | 79901 | <0.001 | 0.58 |
| **134** | **COL1A2** | 1278 | <0.001 | 0.58 |
| **135** | **ROR2** | 4920 | <0.001 | 0.58 |
| **136** | **SPP1** | 6696 | <0.001 | 0.58 |
| **137** | **SERPINE1** | 5054 | <0.001 | 0.58 |
| **138** | **TYROBP** | 7305 | <0.001 | 0.59 |
| **139** | **CXCL1** | 2919 | <0.001 | 0.59 |
| **140** | **MYLK** | 4638 | <0.001 | 0.59 |
| **141** | **THBS1** | 7057 | <0.001 | 0.59 |
| **142** | **NR4A2** | 4929 | <0.001 | 0.59 |
| **143** | **LMO3** | 55885 | <0.001 | 0.59 |
| **144** | **LAMA4** | 3910 | <0.001 | 0.59 |
| **145** | **APBB1IP** | 54518 | <0.001 | 0.59 |
| **146** | **CDKN3** | 1033 | <0.001 | 0.59 |
| **147** | **TTK** | 7272 | <0.001 | 0.59 |
| **148** | **ANGPTL2** | 23452 | <0.001 | 0.59 |
| **149** | **UHRF1** | 29128 | <0.001 | 0.59 |
| **150** | **MMP9** | 4318 | <0.001 | 0.59 |
| **151** | **MFAP5** | 8076 | <0.001 | 0.59 |
| **152** | **GADD45B** | 4616 | <0.001 | 0.59 |
| **153** | **KIF2C** | 11004 | <0.001 | 0.59 |
| **154** | **ASPN** | 54829 | <0.001 | 0.59 |
| **155** | **MELK** | 9833 | <0.001 | 0.59 |
| **156** | **SPRR1B** | 6699 | <0.001 | 0.60 |
| **157** | **ATP6V1B1** | 525 | <0.001 | 0.60 |
| **158** | **HMOX1** | 3162 | <0.001 | 0.60 |
| **159** | **CPXM2** | 119587 | <0.001 | 0.60 |
| **160** | **TROAP** | 10024 | <0.001 | 0.60 |
| **161** | **CDH2** | 1000 | <0.001 | 0.60 |
| **162** | **GGH** | 8836 | <0.001 | 0.60 |
| **163** | **E2F2** | 1870 | <0.001 | 0.60 |
| **164** | **SRPX** | 8406 | <0.001 | 0.60 |
| **165** | **CTSK** | 1513 | <0.001 | 0.60 |
| **166** | **COL15A1** | 1306 | <0.001 | 0.60 |
| **167** | **PHGDH** | 26227 | <0.001 | 0.60 |
| **168** | **DLG7** | 9787 | <0.001 | 0.60 |
| **169** | **POLQ** | 10721 | <0.001 | 0.60 |
| **170** | **RARRES2** | 5919 | <0.001 | 0.60 |
| **171** | **CPVL** | 54504 | <0.001 | 0.60 |
| **172** | **JAM3** | 83700 | <0.001 | 0.61 |
| **173** | **PDGFRB** | 5159 | <0.001 | 0.61 |
| **174** | **GAS1** | 2619 | <0.001 | 0.61 |
| **175** | **RAD54L** | 8438 | <0.001 | 0.61 |
| **176** | **PLAC9** | 219348 | <0.001 | 0.61 |
| **177** | **LEPREL2** | 10536 | <0.001 | 0.61 |
| **178** | **COL6A2** | 1292 | <0.001 | 0.61 |
| **179** | **SMTN** | 6525 | <0.001 | 0.62 |
| **180** | **PTGDS** | 5730 | <0.001 | 0.62 |
| **181** | **IFI30** | 10437 | <0.001 | 0.62 |
| **182** | **ENO2** | 2026 | <0.001 | 0.62 |
| **183** | **EBI2** | 1880 | <0.001 | 0.62 |
| **184** | **KIF20A** | 10112 | <0.001 | 0.62 |
| **185** | **AURKB** | 9212 | <0.001 | 0.62 |
| **186** | **ANLN** | 54443 | <0.001 | 0.62 |
| **187** | **GAS6** | 2621 | <0.001 | 0.62 |
| **188** | **RNASE1** | 6035 | <0.001 | 0.63 |
| **189** | **COL4A2** | 1284 | <0.001 | 0.63 |
| **190** | **ANKRD25** | 25959 | <0.001 | 0.63 |
| **191** | **STC2** | 8614 | <0.001 | 0.63 |
| **192** | **CDCA7** | 83879 | <0.001 | 0.63 |
| **193** | **FBLN2** | 2199 | <0.001 | 0.63 |
| **194** | **IFITM3** | 10410 | <0.001 | 0.63 |
| **195** | **MSN** | 4478 | <0.001 | 0.63 |
| **196** | **OSBPL10** | 114884 | <0.001 | 0.63 |
| **197** | **AXUD1** | 64651 | <0.001 | 0.63 |
| **198** | **ANTXR2** | 118429 | <0.001 | 0.63 |
| **199** | **HDC** | 3067 | <0.001 | 0.64 |
| **200** | **SPAG5** | 10615 | <0.001 | 0.64 |
| **201** | **SETBP1** | 26040 | <0.001 | 0.64 |
| **202** | **TMOD1** | 7111 | <0.001 | 0.64 |
| **203** | **ANXA5** | 308 | <0.001 | 0.64 |
| **204** | **C17orf53** | 78995 | <0.001 | 0.64 |
| **205** | **CDCA3** | 83461 | <0.001 | 0.64 |
| **206** | **CDH11** | 1009 | <0.001 | 0.64 |
| **207** | **CDCA8** | 55143 | <0.001 | 0.64 |
| **208** | **ALOX5AP** | 241 | <0.001 | 0.65 |
| **209** | **OLFML2A** | 169611 | <0.001 | 0.65 |
| **210** | **PTTG1** | 9232 | <0.001 | 0.65 |
| **211** | **RECQL4** | 9401 | <0.001 | 0.65 |
| **212** | **SOCS3** | 9021 | <0.001 | 0.65 |
| **213** | **MAFB** | 9935 | <0.001 | 0.65 |
| **214** | **CENPE** | 1062 | <0.001 | 0.65 |
| **215** | **FST** | 10468 | <0.001 | 0.65 |
| **216** | **ITGB2** | 3689 | <0.001 | 0.65 |
| **217** | **PODN** | 127435 | <0.001 | 0.65 |
| **218** | **FAM64A** | 54478 | <0.001 | 0.65 |
| **219** | **BUB1B** | 701 | <0.001 | 0.65 |
| **220** | **RAD51AP1** | 10635 | <0.001 | 0.65 |
| **221** | **EDNRA** | 1909 | <0.001 | 0.66 |
| **222** | **MCM2** | 4171 | <0.001 | 0.66 |
| **223** | **CAV1** | 857 | <0.001 | 0.66 |
| **224** | **PBK** | 55872 | <0.001 | 0.66 |
| **225** | **LTB4R** | 1241 | <0.001 | 0.66 |
| **226** | **MAP1B** | 4131 | <0.001 | 0.66 |
| **227** | **LMOD1** | 25802 | <0.001 | 0.66 |
| **228** | **TYMS** | 7298 | <0.001 | 0.66 |
| **229** | **F3** | 2152 | <0.001 | 0.66 |
| **230** | **STC1** | 6781 | <0.001 | 0.66 |
| **231** | **TRIB1** | 10221 | <0.001 | 0.66 |
| **232** | **CTSG** | 1511 | <0.001 | 0.66 |
| **233** | **SLIT2** | 9353 | <0.001 | 0.66 |
| **234** | **DOCK2** | 1794 | <0.001 | 0.66 |
| **235** | **CDC2** | 983 | <0.001 | 0.66 |
| **236** | **CAV2** | 858 | <0.001 | 0.67 |
| **237** | **NFIB** | 4781 | <0.001 | 0.67 |
| **238** | **MS4A6A** | 64231 | <0.001 | 0.67 |
| **239** | **NFKBIZ** | 64332 | <0.001 | 0.67 |
| **240** | **COMP** | 1311 | <0.001 | 0.68 |
| **241** | **PYCR1** | 5831 | <0.001 | 0.68 |
| **242** | **SLC1A3** | 6507 | <0.001 | 0.68 |
| **243** | **ECGF1** | 1890 | <0.001 | 0.68 |
| **244** | **BUB1** | 699 | <0.001 | 0.68 |
| **245** | **LIMS2** | 55679 | <0.001 | 0.69 |
| **246** | **PCOLCE2** | 26577 | <0.001 | 0.69 |
| **247** | **EMILIN1** | 11117 | <0.001 | 0.69 |
| **248** | **CHRM3** | 1131 | <0.001 | 0.69 |
| **249** | **PRDM8** | 56978 | <0.001 | 0.69 |
| **250** | **FGL2** | 10875 | <0.001 | 0.70 |
| **251** | **CHAF1B** | 8208 | <0.001 | 0.70 |
| **252** | **LOC91461** | 91461 | <0.001 | 0.70 |
| **253** | **PDE5A** | 8654 | <0.001 | 0.70 |
| **254** | **SNF1LK** | 150094 | <0.001 | 0.70 |
| **255** | **MOXD1** | 26002 | <0.001 | 0.70 |
| **256** | **HCST** | 10870 | <0.001 | 0.71 |
| **257** | **CXCL13** | 10563 | <0.001 | 0.73 |
| **258** | **COLEC12** | 81035 | <0.001 | 0.76 |
| **259** | **CD19** | 930 | <0.001 | 0.76 |
| **260** | **ZNF342** | 162979 | <0.001 | 1.44 |
| **261** | **HLA-DQB2** | 3120 | <0.001 | 1.45 |
| **262** | **GCLC** | 2729 | <0.001 | 1.45 |
| **263** | **ZNF91** | 7644 | <0.001 | 1.46 |
| **264** | **USMG5** | 84833 | <0.001 | 1.46 |
| **265** | **TBX1** | 6899 | <0.001 | 1.46 |
| **266** | **SEMA4B** | 10509 | <0.001 | 1.47 |
| **267** | **ARHGEF16** | 27237 | <0.001 | 1.47 |
| **268** | **LOC146439** | 146439 | <0.001 | 1.48 |
| **269** | **PYGL** | 5836 | <0.001 | 1.48 |
| **270** | **RPL14** | 9045 | <0.001 | 1.49 |
| **271** | **ELF5** | 2001 | <0.001 | 1.50 |
| **272** | **KIAA0746** | 23231 | <0.001 | 1.50 |
| **273** | **APOB48R** | 55911 | <0.001 | 1.50 |
| **274** | **PHACS** | 84680 | <0.001 | 1.50 |
| **275** | **GJB4** | 127534 | <0.001 | 1.51 |
| **276** | **ITM2C** | 81618 | <0.001 | 1.51 |
| **277** | **GPR126** | 57211 | <0.001 | 1.52 |
| **278** | **GDPD2** | 54857 | <0.001 | 1.52 |
| **279** | **EFNB1** | 1947 | <0.001 | 1.52 |
| **280** | **HOXB2** | 3212 | <0.001 | 1.52 |
| **281** | **ICHTHYIN** | 348938 | <0.001 | 1.52 |
| **282** | **PAX8** | 7849 | <0.001 | 1.53 |
| **283** | **SDCBP2** | 27111 | <0.001 | 1.53 |
| **284** | **CD96** | 10225 | <0.001 | 1.53 |
| **285** | **HOXB7** | 3217 | <0.001 | 1.54 |
| **286** | **C20orf42** | 55612 | <0.001 | 1.54 |
| **287** | **UST** | 10090 | <0.001 | 1.54 |
| **288** | **GJC1** | 125111 | <0.001 | 1.55 |
| **289** | **NOXA1** | 10811 | <0.001 | 1.55 |
| **290** | **BTG2** | 7832 | <0.001 | 1.56 |
| **291** | **ZNF135** | 7694 | <0.001 | 1.56 |
| **292** | **CKMT1B** | 1159 | <0.001 | 1.56 |
| **293** | **FLRT3** | 23767 | <0.001 | 1.57 |
| **294** | **FAAH** | 2166 | <0.001 | 1.57 |
| **295** | **ENTPD5** | 957 | <0.001 | 1.57 |
| **296** | **LRMP** | 4033 | <0.001 | 1.58 |
| **297** | **ARHGEF19** | 128272 | <0.001 | 1.59 |
| **298** | **SLC4A11** | 83959 | <0.001 | 1.60 |
| **299** | **VAV3** | 10451 | <0.001 | 1.60 |
| **300** | **C9orf95** | 54981 | <0.001 | 1.61 |
| **301** | **HS3ST1** | 9957 | <0.001 | 1.61 |
| **302** | **CYBA** | 1535 | <0.001 | 1.61 |
| **303** | **ITGA3** | 3675 | <0.001 | 1.62 |
| **304** | **DKK4** | 27121 | <0.001 | 1.63 |
| **305** | **LOC130576** | 130576 | <0.001 | 1.64 |
| **306** | **HOXB4** | 3214 | <0.001 | 1.64 |
| **307** | **COL4A5** | 1287 | <0.001 | 1.64 |
| **308** | **CASQ1** | 844 | <0.001 | 1.64 |
| **309** | **CXADR** | 1525 | <0.001 | 1.65 |
| **310** | **HES5** | 388585 | <0.001 | 1.66 |
| **311** | **SCAMP5** | 192683 | <0.001 | 1.66 |
| **312** | **C8orf42** | 157695 | <0.001 | 1.66 |
| **313** | **CAPN5** | 726 | <0.001 | 1.66 |
| **314** | **RAB25** | 57111 | <0.001 | 1.66 |
| **315** | **CKMT1A** | 548596 | <0.001 | 1.66 |
| **316** | **PLEK2** | 26499 | <0.001 | 1.67 |
| **317** | **DUSP2** | 1844 | <0.001 | 1.68 |
| **318** | **SRPX2** | 27286 | <0.001 | 1.69 |
| **319** | **MGC33846** | 220382 | <0.001 | 1.70 |
| **320** | **PLA2G2F** | 64600 | <0.001 | 1.70 |
| **321** | **SLC7A4** | 6545 | <0.001 | 1.72 |
| **322** | **LAMB3** | 3914 | <0.001 | 1.73 |
| **323** | **KRTCAP3** | 200634 | <0.001 | 1.73 |
| **324** | **SEMA3F** | 6405 | <0.001 | 1.73 |
| **325** | **ITGA2** | 3673 | <0.001 | 1.75 |
| **326** | **TRIM2** | 23321 | <0.001 | 1.76 |
| **327** | **LOC387934** | 387934 | <0.001 | 1.76 |
| **328** | **WNT10A** | 80326 | <0.001 | 1.76 |
| **329** | **C10orf33** | 84795 | <0.001 | 1.77 |
| **330** | **C1orf116** | 79098 | <0.001 | 1.77 |
| **331** | **SDC1** | 6382 | <0.001 | 1.79 |
| **332** | **ALDH7A1** | 501 | <0.001 | 1.79 |
| **333** | **FXYD3** | 5349 | <0.001 | 1.79 |
| **334** | **LAD1** | 3898 | <0.001 | 1.81 |
| **335** | **PTPN13** | 5783 | <0.001 | 1.81 |
| **336** | **CNTN3** | 5067 | <0.001 | 1.82 |
| **337** | **SEMA6A** | 57556 | <0.001 | 1.82 |
| **338** | **ZNF385** | 25946 | <0.001 | 1.82 |
| **339** | **GATA3** | 2625 | <0.001 | 1.83 |
| **340** | **ABCC4** | 10257 | <0.001 | 1.85 |
| **341** | **FOXQ1** | 94234 | <0.001 | 1.86 |
| **342** | **ENTPD3** | 956 | <0.001 | 1.87 |
| **343** | **HOXA5** | 3202 | <0.001 | 1.88 |
| **344** | **ERN2** | 10595 | <0.001 | 1.88 |
| **345** | **CYP4F12** | 66002 | <0.001 | 1.88 |
| **346** | **PLEKHH1** | 57475 | <0.001 | 1.88 |
| **347** | **PTK6** | 5753 | <0.001 | 1.88 |
| **348** | **MLPH** | 79083 | <0.001 | 1.89 |
| **349** | **HAS3** | 3038 | <0.001 | 1.89 |
| **350** | **ATF7IP2** | 80063 | <0.001 | 1.91 |
| **351** | **CTSH** | 1512 | <0.001 | 1.92 |
| **352** | **LOC388743** | 388743 | <0.001 | 1.92 |
| **353** | **VIPR1** | 7433 | <0.001 | 1.93 |
| **354** | **BMP7** | 655 | <0.001 | 1.93 |
| **355** | **DUOX1** | 53905 | <0.001 | 1.93 |
| **356** | **AGR2** | 10551 | <0.001 | 1.94 |
| **357** | **PLCD3** | 113026 | <0.001 | 1.94 |
| **358** | **FOXA1** | 3169 | <0.001 | 1.94 |
| **359** | **CDH23** | 64072 | <0.001 | 1.95 |
| **360** | **LOC92196** | 92196 | <0.001 | 1.96 |
| **361** | **CA12** | 771 | <0.001 | 1.97 |
| **362** | **HOXB3** | 3213 | <0.001 | 1.97 |
| **363** | **SOX15** | 6665 | <0.001 | 1.97 |
| **364** | **MST1R** | 4486 | <0.001 | 1.98 |
| **365** | **IGFBP2** | 3485 | <0.001 | 1.98 |
| **366** | **SSH3** | 54961 | <0.001 | 1.99 |
| **367** | **CYP2J2** | 1573 | <0.001 | 2.01 |
| **368** | **CAPN9** | 10753 | <0.001 | 2.01 |
| **369** | **FAM3D** | 131177 | <0.001 | 2.02 |
| **370** | **SORL1** | 6653 | <0.001 | 2.02 |
| **371** | **BHLHB3** | 79365 | <0.001 | 2.02 |
| **372** | **ALDH4A1** | 8659 | <0.001 | 2.04 |
| **373** | **MGC39715** | 169166 | <0.001 | 2.05 |
| **374** | **UNC5B** | 219699 | <0.001 | 2.05 |
| **375** | **ABCC3** | 8714 | <0.001 | 2.05 |
| **376** | **INA** | 9118 | <0.001 | 2.07 |
| **377** | **SNCG** | 6623 | <0.001 | 2.07 |
| **378** | **C10orf58** | 84293 | <0.001 | 2.07 |
| **379** | **PPFIBP2** | 8495 | <0.001 | 2.08 |
| **380** | **CAPNS2** | 84290 | <0.001 | 2.09 |
| **381** | **SMAD6** | 4091 | <0.001 | 2.09 |
| **382** | **HOXD1** | 3231 | <0.001 | 2.14 |
| **383** | **SYTL1** | 84958 | <0.001 | 2.14 |
| **384** | **CYP4B1** | 1580 | <0.001 | 2.15 |
| **385** | **FBP1** | 2203 | <0.001 | 2.16 |
| **386** | **FGFR3** | 2261 | <0.001 | 2.17 |
| **387** | **PSCA** | 8000 | <0.001 | 2.18 |
| **388** | **CYP3A5** | 1577 | <0.001 | 2.18 |
| **389** | **LOC388610** | 388610 | <0.001 | 2.19 |
| **390** | **PROM2** | 150696 | <0.001 | 2.20 |
| **391** | **C3orf54** | 389119 | <0.001 | 2.22 |
| **392** | **TP73L** | 8626 | <0.001 | 2.22 |
| **393** | **GPX2** | 2877 | <0.001 | 2.23 |
| **394** | **CARD11** | 84433 | <0.001 | 2.24 |
| **395** | **LY6D** | 8581 | <0.001 | 2.27 |
| **396** | **COL17A1** | 1308 | <0.001 | 2.29 |
| **397** | **PLA2G10** | 8399 | <0.001 | 2.32 |
| **398** | **FABP6** | 2172 | <0.001 | 2.33 |
| **399** | **GATA2** | 2624 | <0.001 | 2.40 |
| **400** | **CDC42EP5** | 148170 | <0.001 | 2.45 |
| **401** | **HOXB5** | 3215 | <0.001 | 2.45 |
| **402** | **SLITRK6** | 84189 | <0.001 | 2.57 |
| **403** | **HMGCS2** | 3158 | <0.001 | 2.60 |
| **404** | **AQP3** | 360 | <0.001 | 2.61 |
| **405** | **HOXB8** | 3218 | <0.001 | 2.63 |
| **406** | **ATOH8** | 84913 | <0.001 | 2.67 |
| **407** | **FABP4** | 2167 | <0.001 | 2.76 |
| **408** | **BCAS1** | 8537 | <0.001 | 2.76 |
| **409** | **SLC14A1** | 6563 | <0.001 | 2.82 |
| **410** | **SYT8** | 90019 | <0.001 | 2.87 |
| **411** | **HSD17B2** | 3294 | <0.001 | 2.92 |
| **412** | **D4S234E** | 27065 | <0.001 | 2.97 |
| **413** | **TMPRSS4** | 56649 | <0.001 | 3.20 |
| **414** | **CTSE** | 1510 | <0.001 | 3.30 |
| **415** | **TESC** | 54997 | <0.001 | 3.62 |
| **416** | **ANXA10** | 11199 | <0.001 | 4.50 |
| **417** | **CRTAC1** | 55118 | <0.001 | 6.23 |

* The parametic *P*-values were obtained by two sample t-tests.

^†^ Genes were sorted by fold change values.

Abbreviations: NMIBC, non-muscle invasive bladder cancer; MIBC, muscle invasive bladder cancer
