# Supplementary material for: Identification of Gene Expression Signature Modulated by Nicotinamide in a Mouse Bladder Cancer Model
Source: PLoS One. 2011 Oct 10;6(10):e26131. doi: 10.1371/journal.pone.0026131 (PMC3189956; doi:10.1371/journal.pone.0026131)
Supplement: Table S5 — Significant gene list (623 genes) applied to gene expression-based prediction methods (Comparison between Normal and NMIBC in human). (DOC) [file pone.0026131.s011.doc]

**Table S5. Significant gene list (623 genes) applied to gene expression-based prediction methods (Comparison between Normal and NMIBC in human)**

| **No.** | **Gene symbol** | **Entrez ID** | ***Parametric**  ***P*-value** | **^†^Fold-change (Expression ratio of Normal/NMIBC)** |
| --- | --- | --- | --- | --- |
| **1** | **ESM1** | 11082 | <0.001 | 0.3 |
| **2** | **IGF2** | 3481 | <0.001 | 0.31 |
| **3** | **CDC20** | 991 | <0.001 | 0.32 |
| **4** | **CA9** | 768 | <0.001 | 0.33 |
| **5** | **CTSE** | 1510 | <0.001 | 0.34 |
| **6** | **PODXL2** | 50512 | <0.001 | 0.34 |
| **7** | **C20orf46** | 55321 | <0.001 | 0.35 |
| **8** | **EEF1A2** | 1917 | <0.001 | 0.35 |
| **9** | **FABP6** | 2172 | <0.001 | 0.37 |
| **10** | **ETV4** | 2118 | <0.001 | 0.38 |
| **11** | **TOP2A** | 7153 | <0.001 | 0.39 |
| **12** | **SLC6A8** | 6535 | <0.001 | 0.4 |
| **13** | **PLA2G2F** | 64600 | <0.001 | 0.41 |
| **14** | **CALML3** | 810 | <0.001 | 0.42 |
| **15** | **NUSAP1** | 51203 | <0.001 | 0.42 |
| **16** | **UBE2C** | 11065 | <0.001 | 0.42 |
| **17** | **TTK** | 7272 | <0.001 | 0.43 |
| **18** | **CDC14B** | 8555 | <0.001 | 0.44 |
| **19** | **HOOK1** | 51361 | <0.001 | 0.44 |
| **20** | **HOXB5** | 3215 | <0.001 | 0.44 |
| **21** | **MMP1** | 4312 | <0.001 | 0.44 |
| **22** | **PAFAH1B3** | 5050 | <0.001 | 0.44 |
| **23** | **PRSS8** | 5652 | <0.001 | 0.44 |
| **24** | **PVRL4** | 81607 | <0.001 | 0.44 |
| **25** | **CELSR3** | 1951 | <0.001 | 0.45 |
| **26** | **IGSF9** | 57549 | <0.001 | 0.45 |
| **27** | **KIF20A** | 10112 | <0.001 | 0.45 |
| **28** | **TPX2** | 22974 | <0.001 | 0.45 |
| **29** | **SLC4A11** | 83959 | <0.001 | 0.46 |
| **30** | **TFAP2A** | 7020 | <0.001 | 0.46 |
| **31** | **INA** | 9118 | <0.001 | 0.47 |
| **32** | **IRX3** | 79191 | <0.001 | 0.47 |
| **33** | **LAD1** | 3898 | <0.001 | 0.47 |
| **34** | **C9orf140** | 89958 | <0.001 | 0.48 |
| **35** | **CCNB2** | 9133 | <0.001 | 0.48 |
| **36** | **CDCA5** | 113130 | <0.001 | 0.48 |
| **37** | **CENPF** | 1063 | <0.001 | 0.48 |
| **38** | **DSCR6** | 53820 | <0.001 | 0.48 |
| **39** | **EVPL** | 2125 | <0.001 | 0.48 |
| **40** | **FASN** | 2194 | <0.001 | 0.48 |
| **41** | **FGF11** | 2256 | <0.001 | 0.48 |
| **42** | **HIG2** | 29923 | <0.001 | 0.48 |
| **43** | **HOXB9** | 3219 | <0.001 | 0.48 |
| **44** | **SEMA6A** | 57556 | <0.001 | 0.48 |
| **45** | **UHRF1** | 29128 | <0.001 | 0.48 |
| **46** | **AURKB** | 9212 | <0.001 | 0.49 |
| **47** | **HOXB8** | 3218 | <0.001 | 0.49 |
| **48** | **PRC1** | 9055 | <0.001 | 0.49 |
| **49** | **TBX1** | 6899 | <0.001 | 0.49 |
| **50** | **CABP4** | 57010 | <0.001 | 0.5 |
| **51** | **CDH3** | 1001 | <0.001 | 0.5 |
| **52** | **EPHA1** | 2041 | <0.001 | 0.5 |
| **53** | **GPT2** | 84706 | <0.001 | 0.5 |
| **54** | **SDC1** | 6382 | <0.001 | 0.5 |
| **55** | **SEMA3F** | 6405 | <0.001 | 0.5 |
| **56** | **CBLC** | 23624 | <0.001 | 0.51 |
| **57** | **E2F2** | 1870 | <0.001 | 0.51 |
| **58** | **FOXJ1** | 2302 | <0.001 | 0.51 |
| **59** | **MYT1** | 4661 | <0.001 | 0.51 |
| **60** | **SCAMP5** | 192683 | <0.001 | 0.51 |
| **61** | **SLC2A1** | 6513 | <0.001 | 0.51 |
| **62** | **SLITRK6** | 84189 | <0.001 | 0.51 |
| **63** | **SOX4** | 6659 | <0.001 | 0.51 |
| **64** | **TK1** | 7083 | <0.001 | 0.51 |
| **65** | **ARHGEF19** | 128272 | <0.001 | 0.52 |
| **66** | **ASF1B** | 55723 | <0.001 | 0.52 |
| **67** | **CDS1** | 1040 | <0.001 | 0.52 |
| **68** | **EPS8L1** | 54869 | <0.001 | 0.52 |
| **69** | **F12** | 2161 | <0.001 | 0.52 |
| **70** | **MYH14** | 79784 | <0.001 | 0.52 |
| **71** | **TRIP13** | 9319 | <0.001 | 0.52 |
| **72** | **BHLHB3** | 79365 | <0.001 | 0.53 |
| **73** | **C20orf94** | 128710 | <0.001 | 0.53 |
| **74** | **KIF2C** | 11004 | <0.001 | 0.53 |
| **75** | **PAQR4** | 124222 | <0.001 | 0.53 |
| **76** | **EPHB6** | 2051 | <0.001 | 0.54 |
| **77** | **FAM83A** | 84985 | <0.001 | 0.54 |
| **78** | **HOXC4** | 3221 | <0.001 | 0.54 |
| **79** | **PROM2** | 150696 | <0.001 | 0.54 |
| **80** | **PTK6** | 5753 | <0.001 | 0.54 |
| **81** | **SLC5A3** | 6526 | <0.001 | 0.54 |
| **82** | **SLC7A4** | 6545 | <0.001 | 0.54 |
| **83** | **TROAP** | 10024 | <0.001 | 0.54 |
| **84** | **ZNF342** | 162979 | <0.001 | 0.54 |
| **85** | **MST1R** | 4486 | <0.001 | 0.55 |
| **86** | **PTPRR** | 5801 | <0.001 | 0.55 |
| **87** | **SCNN1G** | 6340 | <0.001 | 0.55 |
| **88** | **TACC3** | 10460 | <0.001 | 0.55 |
| **89** | **FGFR3** | 2261 | <0.001 | 0.56 |
| **90** | **HIC2** | 23119 | <0.001 | 0.56 |
| **91** | **HOXB4** | 3214 | <0.001 | 0.56 |
| **92** | **HOXB7** | 3217 | <0.001 | 0.56 |
| **93** | **RAD54L** | 8438 | <0.001 | 0.56 |
| **94** | **TLE6** | 79816 | <0.001 | 0.56 |
| **95** | **TNNT1** | 7138 | <0.001 | 0.56 |
| **96** | **CDCA3** | 83461 | <0.001 | 0.57 |
| **97** | **CKMT1A** | 548596 | <0.001 | 0.57 |
| **98** | **CKMT1B** | 1159 | <0.001 | 0.57 |
| **99** | **LOC642393** | 642393 | <0.001 | 0.57 |
| **100** | **MMP10** | 4319 | <0.001 | 0.57 |
| **101** | **POU5F1** | 5460 | <0.001 | 0.57 |
| **102** | **RAB25** | 57111 | <0.001 | 0.57 |
| **103** | **SFN** | 2810 | <0.001 | 0.57 |
| **104** | **SLC15A1** | 6564 | <0.001 | 0.57 |
| **105** | **SPAG5** | 10615 | <0.001 | 0.57 |
| **106** | **TH** | 7054 | <0.001 | 0.57 |
| **107** | **C17orf53** | 78995 | <0.001 | 0.58 |
| **108** | **CCNA2** | 890 | <0.001 | 0.58 |
| **109** | **CDCA8** | 55143 | <0.001 | 0.58 |
| **110** | **CYP2J2** | 1573 | <0.001 | 0.58 |
| **111** | **FAAH** | 2166 | <0.001 | 0.58 |
| **112** | **GPC2** | 221914 | <0.001 | 0.58 |
| **113** | **HAS3** | 3038 | <0.001 | 0.58 |
| **114** | **KIAA0101** | 9768 | <0.001 | 0.58 |
| **115** | **NDRG4** | 65009 | <0.001 | 0.58 |
| **116** | **POLQ** | 10721 | <0.001 | 0.58 |
| **117** | **RECQL4** | 9401 | <0.001 | 0.58 |
| **118** | **BUB1B** | 701 | <0.001 | 0.59 |
| **119** | **C3orf54** | 389119 | <0.001 | 0.59 |
| **120** | **FCHO1** | 23149 | <0.001 | 0.59 |
| **121** | **HIST1H4K** | 8362 | <0.001 | 0.59 |
| **122** | **PLEK2** | 26499 | <0.001 | 0.59 |
| **123** | **SCNN1B** | 6338 | <0.001 | 0.59 |
| **124** | **CARD11** | 84433 | <0.001 | 0.6 |
| **125** | **FAM3D** | 131177 | <0.001 | 0.6 |
| **126** | **HES6** | 55502 | <0.001 | 0.6 |
| **127** | **HIST2H2AC** | 8338 | <0.001 | 0.6 |
| **128** | **S100A14** | 57402 | <0.001 | 0.6 |
| **129** | **SLC9A2** | 6549 | <0.001 | 0.6 |
| **130** | **BUB1** | 699 | <0.001 | 0.61 |
| **131** | **FGD5** | 152273 | <0.001 | 0.61 |
| **132** | **FXYD3** | 5349 | <0.001 | 0.61 |
| **133** | **GJC1** | 125111 | <0.001 | 0.61 |
| **134** | **MDK** | 4192 | <0.001 | 0.61 |
| **135** | **PLEKHH1** | 57475 | <0.001 | 0.61 |
| **136** | **RASEF** | 158158 | <0.001 | 0.61 |
| **137** | **RHPN1** | 114822 | <0.001 | 0.61 |
| **138** | **TRPV4** | 59341 | <0.001 | 0.61 |
| **139** | **ANLN** | 54443 | <0.001 | 0.62 |
| **140** | **FAM64A** | 54478 | <0.001 | 0.62 |
| **141** | **GJB4** | 127534 | <0.001 | 0.62 |
| **142** | **GPR81** | 27198 | <0.001 | 0.62 |
| **143** | **KRTCAP3** | 200634 | <0.001 | 0.62 |
| **144** | **LOC89944** | 89944 | <0.001 | 0.62 |
| **145** | **MCM2** | 4171 | <0.001 | 0.62 |
| **146** | **RAB3IP** | 117177 | <0.001 | 0.62 |
| **147** | **SPP1** | 6696 | <0.001 | 0.62 |
| **148** | **TNFRSF25** | 8718 | <0.001 | 0.62 |
| **149** | **CLIC3** | 9022 | <0.001 | 0.63 |
| **150** | **DLG7** | 9787 | <0.001 | 0.63 |
| **151** | **EFNB1** | 1947 | <0.001 | 0.63 |
| **152** | **ITGA3** | 3675 | <0.001 | 0.63 |
| **153** | **OAS2** | 4939 | <0.001 | 0.63 |
| **154** | **SSH3** | 54961 | <0.001 | 0.63 |
| **155** | **TYMS** | 7298 | <0.001 | 0.63 |
| **156** | **ABHD9** | 79852 | <0.001 | 0.64 |
| **157** | **ARHGEF16** | 27237 | <0.001 | 0.64 |
| **158** | **BAG4** | 9530 | <0.001 | 0.64 |
| **159** | **CDC2** | 983 | <0.001 | 0.64 |
| **160** | **FAM63B** | 54629 | <0.001 | 0.64 |
| **161** | **FSCN1** | 6624 | <0.001 | 0.64 |
| **162** | **IGFBP4** | 3487 | <0.001 | 0.64 |
| **163** | **PTTG1** | 9232 | <0.001 | 0.64 |
| **164** | **ZNF385** | 25946 | <0.001 | 0.64 |
| **165** | **APOC1** | 341 | <0.001 | 0.65 |
| **166** | **PBK** | 55872 | <0.001 | 0.65 |
| **167** | **SEMA4B** | 10509 | <0.001 | 0.65 |
| **168** | **SNCG** | 6623 | <0.001 | 0.65 |
| **169** | **GATA3** | 2625 | <0.001 | 0.66 |
| **170** | **GDPD2** | 54857 | <0.001 | 0.66 |
| **171** | **HOXB3** | 3213 | <0.001 | 0.66 |
| **172** | **KIFC2** | 90990 | <0.001 | 0.66 |
| **173** | **KPNA2** | 3838 | <0.001 | 0.66 |
| **174** | **MELK** | 9833 | <0.001 | 0.66 |
| **175** | **PPFIBP2** | 8495 | <0.001 | 0.66 |
| **176** | **RGS17** | 26575 | <0.001 | 0.66 |
| **177** | **SCD** | 6319 | <0.001 | 0.66 |
| **178** | **SLC23A3** | 151295 | <0.001 | 0.66 |
| **179** | **SLC38A5** | 92745 | <0.001 | 0.66 |
| **180** | **TRPV6** | 55503 | <0.001 | 0.66 |
| **181** | **BCAS1** | 8537 | <0.001 | 0.67 |
| **182** | **CGN** | 57530 | <0.001 | 0.67 |
| **183** | **CHAF1B** | 8208 | <0.001 | 0.67 |
| **184** | **HES5** | 388585 | <0.001 | 0.67 |
| **185** | **HMMR** | 3161 | <0.001 | 0.67 |
| **186** | **LAMC2** | 3918 | <0.001 | 0.67 |
| **187** | **LOC146439** | 146439 | <0.001 | 0.67 |
| **188** | **NUP210** | 23225 | <0.001 | 0.67 |
| **189** | **SLC16A9** | 220963 | <0.001 | 0.67 |
| **190** | **SYTL1** | 84958 | <0.001 | 0.67 |
| **191** | **CCND1** | 595 | <0.001 | 0.68 |
| **192** | **CDH23** | 64072 | <0.001 | 0.68 |
| **193** | **ENTPD5** | 957 | <0.001 | 0.68 |
| **194** | **S100A16** | 140576 | <0.001 | 0.68 |
| **195** | **VAV3** | 10451 | <0.001 | 0.68 |
| **196** | **NDRG1** | 10397 | <0.001 | 0.69 |
| **197** | **PKIA** | 5569 | <0.001 | 0.69 |
| **198** | **PNMT** | 5409 | <0.001 | 0.69 |
| **199** | **SMAD6** | 4091 | <0.001 | 0.69 |
| **200** | **DKK4** | 27121 | <0.001 | 0.7 |
| **201** | **NFAT5** | 10725 | <0.001 | 0.7 |
| **202** | **PAX8** | 7849 | <0.001 | 0.7 |
| **203** | **HIST1H2BD** | 3017 | <0.001 | 0.71 |
| **204** | **KIAA1199** | 57214 | <0.001 | 0.71 |
| **205** | **FXYD5** | 53827 | <0.001 | 0.72 |
| **206** | **RAD51AP1** | 10635 | <0.001 | 0.72 |
| **207** | **CENPA** | 1058 | <0.001 | 0.73 |
| **208** | **CNFN** | 84518 | <0.001 | 0.73 |
| **209** | **UNC5B** | 219699 | <0.001 | 0.73 |
| **210** | **CENPE** | 1062 | <0.001 | 0.74 |
| **211** | **PYCR1** | 5831 | <0.001 | 0.74 |
| **212** | **GSTM4** | 2948 | <0.001 | 0.75 |
| **213** | **DSG3** | 1830 | <0.001 | 1.35 |
| **214** | **C9orf58** | 83543 | <0.001 | 1.44 |
| **215** | **FOXF2** | 2295 | <0.001 | 1.44 |
| **216** | **SMPDL3A** | 10924 | <0.001 | 1.44 |
| **217** | **NFKBIZ** | 64332 | <0.001 | 1.45 |
| **218** | **ARHGAP4** | 393 | <0.001 | 1.46 |
| **219** | **ITM2C** | 81618 | <0.001 | 1.46 |
| **220** | **SNF1LK** | 150094 | <0.001 | 1.47 |
| **221** | **SELENBP1** | 8991 | <0.001 | 1.48 |
| **222** | **RPLP0** | 6175 | <0.001 | 1.49 |
| **223** | **POU2AF1** | 5450 | <0.001 | 1.5 |
| **224** | **SNCAIP** | 9627 | <0.001 | 1.5 |
| **225** | **SULF1** | 23213 | <0.001 | 1.5 |
| **226** | **C5orf13** | 9315 | <0.001 | 1.51 |
| **227** | **COL4A5** | 1287 | <0.001 | 1.52 |
| **228** | **NT5E** | 4907 | <0.001 | 1.53 |
| **229** | **LAPTM5** | 7805 | <0.001 | 1.54 |
| **230** | **LDLR** | 3949 | <0.001 | 1.54 |
| **231** | **STEAP2** | 261729 | <0.001 | 1.54 |
| **232** | **RAPGEF3** | 10411 | <0.001 | 1.55 |
| **233** | **RPL7** | 6129 | <0.001 | 1.55 |
| **234** | **HRASLS3** | 11145 | <0.001 | 1.56 |
| **235** | **STC1** | 6781 | <0.001 | 1.56 |
| **236** | **ASPN** | 54829 | <0.001 | 1.57 |
| **237** | **FHL2** | 2274 | <0.001 | 1.57 |
| **238** | **IL1B** | 3553 | <0.001 | 1.57 |
| **239** | **LPXN** | 9404 | <0.001 | 1.57 |
| **240** | **SERPINB11** | 89778 | <0.001 | 1.57 |
| **241** | **TCEA3** | 6920 | <0.001 | 1.57 |
| **242** | **BTG2** | 7832 | <0.001 | 1.58 |
| **243** | **C10orf38** | 221061 | <0.001 | 1.58 |
| **244** | **CD44** | 960 | <0.001 | 1.58 |
| **245** | **HLA-DMA** | 3108 | <0.001 | 1.58 |
| **246** | **TM4SF1** | 4071 | <0.001 | 1.58 |
| **247** | **CCL20** | 6364 | <0.001 | 1.59 |
| **248** | **DNALI1** | 7802 | <0.001 | 1.6 |
| **249** | **TGFBR3** | 7049 | <0.001 | 1.6 |
| **250** | **GSPT2** | 23708 | <0.001 | 1.61 |
| **251** | **SLC39A6** | 25800 | <0.001 | 1.62 |
| **252** | **ZFP36L1** | 677 | <0.001 | 1.63 |
| **253** | **TAC3** | 6866 | <0.001 | 1.64 |
| **254** | **ALDH3A1** | 218 | <0.001 | 1.66 |
| **255** | **PALM** | 5064 | <0.001 | 1.66 |
| **256** | **APBB1IP** | 54518 | <0.001 | 1.67 |
| **257** | **CORO1A** | 11151 | <0.001 | 1.67 |
| **258** | **IGFBP2** | 3485 | <0.001 | 1.67 |
| **259** | **PEG3** | 5178 | <0.001 | 1.67 |
| **260** | **SLPI** | 6590 | <0.001 | 1.68 |
| **261** | **CRIP1** | 1396 | <0.001 | 1.69 |
| **262** | **CXCL9** | 4283 | <0.001 | 1.69 |
| **263** | **SOCS2** | 8835 | <0.001 | 1.69 |
| **264** | **CD79A** | 973 | <0.001 | 1.7 |
| **265** | **IDH1** | 3417 | <0.001 | 1.7 |
| **266** | **MAFB** | 9935 | <0.001 | 1.71 |
| **267** | **FZD10** | 11211 | <0.001 | 1.72 |
| **268** | **TRIM31** | 11074 | <0.001 | 1.72 |
| **269** | **CTHRC1** | 115908 | <0.001 | 1.73 |
| **270** | **DAB2** | 1601 | <0.001 | 1.73 |
| **271** | **PTGER4** | 5734 | <0.001 | 1.73 |
| **272** | **HSPA2** | 3306 | <0.001 | 1.74 |
| **273** | **UBD** | 10537 | <0.001 | 1.74 |
| **274** | **AXUD1** | 64651 | <0.001 | 1.75 |
| **275** | **RPL22** | 6146 | <0.001 | 1.75 |
| **276** | **ADAMTS4** | 9507 | <0.001 | 1.76 |
| **277** | **BHLHB2** | 8553 | <0.001 | 1.76 |
| **278** | **WEE1** | 7465 | <0.001 | 1.76 |
| **279** | **COL8A1** | 1295 | <0.001 | 1.77 |
| **280** | **IL6** | 3569 | <0.001 | 1.78 |
| **281** | **CD19** | 930 | <0.001 | 1.79 |
| **282** | **CYGB** | 114757 | <0.001 | 1.79 |
| **283** | **SCUBE2** | 57758 | <0.001 | 1.79 |
| **284** | **CHPT1** | 56994 | <0.001 | 1.81 |
| **285** | **HLA-DOA** | 3111 | <0.001 | 1.81 |
| **286** | **NXN** | 64359 | <0.001 | 1.81 |
| **287** | **RNASE1** | 6035 | <0.001 | 1.81 |
| **288** | **BTF3** | 689 | <0.001 | 1.82 |
| **289** | **DC2** | 58505 | <0.001 | 1.83 |
| **290** | **KHDRBS3** | 10656 | <0.001 | 1.83 |
| **291** | **LXN** | 56925 | <0.001 | 1.83 |
| **292** | **SCN11A** | 11280 | <0.001 | 1.83 |
| **293** | **BMP4** | 652 | <0.001 | 1.85 |
| **294** | **LTB4DH** | 22949 | <0.001 | 1.85 |
| **295** | **ARMCX1** | 51309 | <0.001 | 1.86 |
| **296** | **RPS4X** | 6191 | <0.001 | 1.86 |
| **297** | **HLA-F** | 3134 | <0.001 | 1.87 |
| **298** | **IFITM3** | 10410 | <0.001 | 1.87 |
| **299** | **LYZ** | 4069 | <0.001 | 1.88 |
| **300** | **ADH1C** | 126 | <0.001 | 1.9 |
| **301** | **PLA2G4A** | 5321 | <0.001 | 1.9 |
| **302** | **SEMA3B** | 7869 | <0.001 | 1.9 |
| **303** | **HCLS1** | 3059 | <0.001 | 1.92 |
| **304** | **TNNT3** | 7140 | <0.001 | 1.92 |
| **305** | **ODC1** | 4953 | <0.001 | 1.93 |
| **306** | **PDE7B** | 27115 | <0.001 | 1.93 |
| **307** | **SLC24A3** | 57419 | <0.001 | 1.93 |
| **308** | **NELL2** | 4753 | <0.001 | 1.94 |
| **309** | **NFIB** | 4781 | <0.001 | 1.95 |
| **310** | **RHOB** | 388 | <0.001 | 1.95 |
| **311** | **RRAS** | 6237 | <0.001 | 1.95 |
| **312** | **SOCS3** | 9021 | <0.001 | 1.95 |
| **313** | **CACNA1H** | 8912 | <0.001 | 1.96 |
| **314** | **CSPG4** | 1464 | <0.001 | 1.97 |
| **315** | **TSPYL1** | 7259 | <0.001 | 1.97 |
| **316** | **FBLN1** | 2192 | <0.001 | 1.98 |
| **317** | **LEPREL1** | 55214 | <0.001 | 1.98 |
| **318** | **POPDC2** | 64091 | <0.001 | 1.98 |
| **319** | **GADD45B** | 4616 | <0.001 | 1.99 |
| **320** | **MSN** | 4478 | <0.001 | 1.99 |
| **321** | **NCALD** | 83988 | <0.001 | 1.99 |
| **322** | **CHRM3** | 1131 | <0.001 | 2 |
| **323** | **COL5A2** | 1290 | <0.001 | 2 |
| **324** | **SCRG1** | 11341 | <0.001 | 2 |
| **325** | **AOC3** | 8639 | <0.001 | 2.02 |
| **326** | **LRIG1** | 26018 | <0.001 | 2.02 |
| **327** | **OLFML2A** | 169611 | <0.001 | 2.03 |
| **328** | **PRDX3** | 10935 | <0.001 | 2.04 |
| **329** | **S100A10** | 6281 | <0.001 | 2.04 |
| **330** | **ITPR1** | 3708 | <0.001 | 2.05 |
| **331** | **CD37** | 951 | <0.001 | 2.06 |
| **332** | **GSTM5** | 2949 | <0.001 | 2.06 |
| **333** | **TNFAIP3** | 7128 | <0.001 | 2.07 |
| **334** | **C6orf189** | 221303 | <0.001 | 2.08 |
| **335** | **NFIA** | 4774 | <0.001 | 2.08 |
| **336** | **HOXA13** | 3209 | <0.001 | 2.09 |
| **337** | **PTPLA** | 9200 | <0.001 | 2.09 |
| **338** | **AP3S1** | 1176 | <0.001 | 2.1 |
| **339** | **AQP1** | 358 | <0.001 | 2.1 |
| **340** | **GJA1** | 2697 | <0.001 | 2.1 |
| **341** | **PDGFRB** | 5159 | <0.001 | 2.1 |
| **342** | **UBE2E2** | 7325 | <0.001 | 2.1 |
| **343** | **CXCR4** | 7852 | <0.001 | 2.11 |
| **344** | **IER3** | 8870 | <0.001 | 2.11 |
| **345** | **C6orf105** | 84830 | <0.001 | 2.12 |
| **346** | **FAM46B** | 115572 | <0.001 | 2.13 |
| **347** | **RARRES1** | 5918 | <0.001 | 2.13 |
| **348** | **LEPREL2** | 10536 | <0.001 | 2.14 |
| **349** | **LOC285382** | 285382 | <0.001 | 2.14 |
| **350** | **SPRR3** | 6707 | <0.001 | 2.14 |
| **351** | **NR4A2** | 4929 | <0.001 | 2.15 |
| **352** | **PDGFD** | 80310 | <0.001 | 2.15 |
| **353** | **TGM2** | 7052 | <0.001 | 2.15 |
| **354** | **NNMT** | 4837 | <0.001 | 2.16 |
| **355** | **UPK3A** | 7380 | <0.001 | 2.16 |
| **356** | **HOXA9** | 3205 | <0.001 | 2.17 |
| **357** | **LOC91461** | 91461 | <0.001 | 2.18 |
| **358** | **SERPING1** | 710 | <0.001 | 2.18 |
| **359** | **LGALS4** | 3960 | <0.001 | 2.19 |
| **360** | **IGFBP7** | 3490 | <0.001 | 2.2 |
| **361** | **EOMES** | 8320 | <0.001 | 2.21 |
| **362** | **THBS1** | 7057 | <0.001 | 2.21 |
| **363** | **CCL5** | 6352 | <0.001 | 2.22 |
| **364** | **F3** | 2152 | <0.001 | 2.22 |
| **365** | **HCST** | 10870 | <0.001 | 2.22 |
| **366** | **NDN** | 4692 | <0.001 | 2.22 |
| **367** | **UCHL1** | 7345 | <0.001 | 2.22 |
| **368** | **LITAF** | 9516 | <0.001 | 2.23 |
| **369** | **FCER1A** | 2205 | <0.001 | 2.24 |
| **370** | **IMPA2** | 3613 | <0.001 | 2.24 |
| **371** | **ALDH1A2** | 8854 | <0.001 | 2.25 |
| **372** | **PLS3** | 5358 | <0.001 | 2.25 |
| **373** | **PTGS2** | 5743 | <0.001 | 2.25 |
| **374** | **CLCA4** | 22802 | <0.001 | 2.26 |
| **375** | **COL1A1** | 1277 | <0.001 | 2.26 |
| **376** | **FLJ21438** | 64926 | <0.001 | 2.26 |
| **377** | **LAMA4** | 3910 | <0.001 | 2.26 |
| **378** | **PLAT** | 5327 | <0.001 | 2.26 |
| **379** | **ANXA5** | 308 | <0.001 | 2.27 |
| **380** | **SETBP1** | 26040 | <0.001 | 2.27 |
| **381** | **CAP2** | 10486 | <0.001 | 2.28 |
| **382** | **PLSCR4** | 57088 | <0.001 | 2.28 |
| **383** | **ACTN1** | 87 | <0.001 | 2.29 |
| **384** | **CTSG** | 1511 | <0.001 | 2.29 |
| **385** | **ACACB** | 32 | <0.001 | 2.3 |
| **386** | **B2M** | 567 | <0.001 | 2.3 |
| **387** | **CD48** | 962 | <0.001 | 2.3 |
| **388** | **SGK** | 6446 | <0.001 | 2.3 |
| **389** | **CAV2** | 858 | <0.001 | 2.31 |
| **390** | **CD14** | 929 | <0.001 | 2.31 |
| **391** | **HLA-DMB** | 3109 | <0.001 | 2.31 |
| **392** | **WNT5A** | 7474 | <0.001 | 2.31 |
| **393** | **FBLN2** | 2199 | <0.001 | 2.32 |
| **394** | **MATN2** | 4147 | <0.001 | 2.32 |
| **395** | **DKK3** | 27122 | <0.001 | 2.33 |
| **396** | **NEXN** | 91624 | <0.001 | 2.33 |
| **397** | **TGFBR2** | 7048 | <0.001 | 2.33 |
| **398** | **CD2** | 914 | <0.001 | 2.34 |
| **399** | **LMO3** | 55885 | <0.001 | 2.34 |
| **400** | **PODN** | 127435 | <0.001 | 2.34 |
| **401** | **LIMS2** | 55679 | <0.001 | 2.37 |
| **402** | **PRICKLE2** | 166336 | <0.001 | 2.37 |
| **403** | **LMCD1** | 29995 | <0.001 | 2.38 |
| **404** | **NFIX** | 4784 | <0.001 | 2.38 |
| **405** | **TGFB1I1** | 7041 | <0.001 | 2.38 |
| **406** | **AXIN2** | 8313 | <0.001 | 2.39 |
| **407** | **GPNMB** | 10457 | <0.001 | 2.39 |
| **408** | **CENTB1** | 9744 | <0.001 | 2.4 |
| **409** | **CKB** | 1152 | <0.001 | 2.4 |
| **410** | **EMP1** | 2012 | <0.001 | 2.4 |
| **411** | **MAP1B** | 4131 | <0.001 | 2.4 |
| **412** | **PIGR** | 5284 | <0.001 | 2.4 |
| **413** | **CD6** | 923 | <0.001 | 2.41 |
| **414** | **CX3CL1** | 6376 | <0.001 | 2.41 |
| **415** | **PRDM8** | 56978 | <0.001 | 2.41 |
| **416** | **RNASE4** | 6038 | <0.001 | 2.42 |
| **417** | **SH3BGRL** | 6451 | <0.001 | 2.42 |
| **418** | **STAB1** | 23166 | <0.001 | 2.42 |
| **419** | **GPX3** | 2878 | <0.001 | 2.43 |
| **420** | **ITGB2** | 3689 | <0.001 | 2.43 |
| **421** | **PDE5A** | 8654 | <0.001 | 2.43 |
| **422** | **PLEK** | 5341 | <0.001 | 2.43 |
| **423** | **GALNAC4S-6ST** | 51363 | <0.001 | 2.44 |
| **424** | **PROM1** | 8842 | <0.001 | 2.44 |
| **425** | **CTSK** | 1513 | <0.001 | 2.46 |
| **426** | **TXNRD1** | 7296 | <0.001 | 2.46 |
| **427** | **CSF1R** | 1436 | <0.001 | 2.47 |
| **428** | **C10orf56** | 219654 | <0.001 | 2.48 |
| **429** | **C1QB** | 713 | <0.001 | 2.48 |
| **430** | **KLF2** | 10365 | <0.001 | 2.49 |
| **431** | **PAM** | 5066 | <0.001 | 2.49 |
| **432** | **CPVL** | 54504 | <0.001 | 2.5 |
| **433** | **IL7R** | 3575 | <0.001 | 2.5 |
| **434** | **LPPR4** | 9890 | <0.001 | 2.5 |
| **435** | **MS4A6A** | 64231 | <0.001 | 2.5 |
| **436** | **TYROBP** | 7305 | <0.001 | 2.5 |
| **437** | **MFAP5** | 8076 | <0.001 | 2.51 |
| **438** | **PELI2** | 57161 | <0.001 | 2.51 |
| **439** | **RASD1** | 51655 | <0.001 | 2.51 |
| **440** | **ZBTB16** | 7704 | <0.001 | 2.51 |
| **441** | **ENPP2** | 5168 | <0.001 | 2.53 |
| **442** | **IGFBP6** | 3489 | <0.001 | 2.53 |
| **443** | **CYP27A1** | 1593 | <0.001 | 2.54 |
| **444** | **PCOLCE2** | 26577 | <0.001 | 2.56 |
| **445** | **HDC** | 3067 | <0.001 | 2.57 |
| **446** | **PRKCDBP** | 112464 | <0.001 | 2.57 |
| **447** | **DKFZP586H2123** | 25891 | <0.001 | 2.58 |
| **448** | **LTBP4** | 8425 | <0.001 | 2.58 |
| **449** | **MAOB** | 4129 | <0.001 | 2.58 |
| **450** | **PPP1R14A** | 94274 | <0.001 | 2.58 |
| **451** | **WFDC1** | 58189 | <0.001 | 2.58 |
| **452** | **MMP7** | 4316 | <0.001 | 2.6 |
| **453** | **GHR** | 2690 | <0.001 | 2.61 |
| **454** | **GAS1** | 2619 | <0.001 | 2.62 |
| **455** | **CSRP1** | 1465 | <0.001 | 2.63 |
| **456** | **GPR124** | 25960 | <0.001 | 2.63 |
| **457** | **LGALS3** | 3958 | <0.001 | 2.63 |
| **458** | **GNG10** | 2790 | <0.001 | 2.64 |
| **459** | **CLIC4** | 25932 | <0.001 | 2.65 |
| **460** | **LGALS1** | 3956 | <0.001 | 2.65 |
| **461** | **SYNPO2** | 171024 | <0.001 | 2.67 |
| **462** | **SLC2A3** | 6515 | <0.001 | 2.68 |
| **463** | **COL15A1** | 1306 | <0.001 | 2.69 |
| **464** | **HSPB8** | 26353 | <0.001 | 2.7 |
| **465** | **PRICKLE1** | 144165 | <0.001 | 2.7 |
| **466** | **GFPT2** | 9945 | <0.001 | 2.71 |
| **467** | **DOCK2** | 1794 | <0.001 | 2.72 |
| **468** | **COLEC12** | 81035 | <0.001 | 2.74 |
| **469** | **CXCL12** | 6387 | <0.001 | 2.74 |
| **470** | **RGS5** | 8490 | <0.001 | 2.74 |
| **471** | **RGS11** | 8786 | <0.001 | 2.77 |
| **472** | **COL3A1** | 1281 | <0.001 | 2.79 |
| **473** | **OLFML3** | 56944 | <0.001 | 2.79 |
| **474** | **ANGPTL2** | 23452 | <0.001 | 2.81 |
| **475** | **CAV1** | 857 | <0.001 | 2.82 |
| **476** | **SFRP1** | 6422 | <0.001 | 2.82 |
| **477** | **SPARC** | 6678 | <0.001 | 2.82 |
| **478** | **NR2F1** | 7025 | <0.001 | 2.83 |
| **479** | **OSBPL10** | 114884 | <0.001 | 2.84 |
| **480** | **EBI2** | 1880 | <0.001 | 2.85 |
| **481** | **SMTN** | 6525 | <0.001 | 2.85 |
| **482** | **C21orf63** | 59271 | <0.001 | 2.87 |
| **483** | **EPB41L3** | 23136 | <0.001 | 2.89 |
| **484** | **FGF9** | 2254 | <0.001 | 2.9 |
| **485** | **LHFP** | 10186 | <0.001 | 2.93 |
| **486** | **SERPINF1** | 5176 | <0.001 | 2.94 |
| **487** | **EDNRA** | 1909 | <0.001 | 2.95 |
| **488** | **RARRES2** | 5919 | <0.001 | 2.95 |
| **489** | **ITM2A** | 9452 | <0.001 | 2.96 |
| **490** | **TGFBI** | 7045 | <0.001 | 2.96 |
| **491** | **C3** | 718 | <0.001 | 2.97 |
| **492** | **CCL19** | 6363 | <0.001 | 2.97 |
| **493** | **GATM** | 2628 | <0.001 | 2.98 |
| **494** | **DKK1** | 22943 | <0.001 | 2.99 |
| **495** | **ITGA5** | 3678 | <0.001 | 3.01 |
| **496** | **ANKRD25** | 25959 | <0.001 | 3.02 |
| **497** | **C1QA** | 712 | <0.001 | 3.02 |
| **498** | **ACOX2** | 8309 | <0.001 | 3.04 |
| **499** | **APOD** | 347 | <0.001 | 3.04 |
| **500** | **MT2A** | 4502 | <0.001 | 3.06 |
| **501** | **TCF21** | 6943 | <0.001 | 3.08 |
| **502** | **GAS6** | 2621 | <0.001 | 3.09 |
| **503** | **ITGA8** | 8516 | <0.001 | 3.09 |
| **504** | **CPXM2** | 119587 | <0.001 | 3.1 |
| **505** | **C2orf32** | 25927 | <0.001 | 3.11 |
| **506** | **GNG11** | 2791 | <0.001 | 3.12 |
| **507** | **A2M** | 2 | <0.001 | 3.13 |
| **508** | **ANXA1** | 301 | <0.001 | 3.14 |
| **509** | **ATF3** | 467 | <0.001 | 3.15 |
| **510** | **ALDH2** | 217 | <0.001 | 3.16 |
| **511** | **PI16** | 221476 | <0.001 | 3.17 |
| **512** | **RBP1** | 5947 | <0.001 | 3.17 |
| **513** | **ISL1** | 3670 | <0.001 | 3.18 |
| **514** | **SLIT2** | 9353 | <0.001 | 3.19 |
| **515** | **DPT** | 1805 | <0.001 | 3.2 |
| **516** | **EMILIN1** | 11117 | <0.001 | 3.2 |
| **517** | **COL5A1** | 1289 | <0.001 | 3.22 |
| **518** | **TGFB3** | 7043 | <0.001 | 3.22 |
| **519** | **DPYSL2** | 1808 | <0.001 | 3.24 |
| **520** | **RNF150** | 57484 | <0.001 | 3.24 |
| **521** | **ALOX5AP** | 241 | <0.001 | 3.27 |
| **522** | **CYR61** | 3491 | <0.001 | 3.28 |
| **523** | **LAMC3** | 10319 | <0.001 | 3.29 |
| **524** | **CCL2** | 6347 | <0.001 | 3.31 |
| **525** | **DIXDC1** | 85458 | <0.001 | 3.31 |
| **526** | **ROR2** | 4920 | <0.001 | 3.33 |
| **527** | **MAMDC2** | 256691 | <0.001 | 3.35 |
| **528** | **THBS2** | 7058 | <0.001 | 3.35 |
| **529** | **TIMP2** | 7077 | <0.001 | 3.35 |
| **530** | **COL6A2** | 1292 | <0.001 | 3.4 |
| **531** | **SERPINA3** | 12 | <0.001 | 3.43 |
| **532** | **C9orf19** | 152007 | <0.001 | 3.45 |
| **533** | **COL6A3** | 1293 | <0.001 | 3.46 |
| **534** | **FGL2** | 10875 | <0.001 | 3.46 |
| **535** | **C8orf4** | 56892 | <0.001 | 3.47 |
| **536** | **PLEKHC1** | 10979 | <0.001 | 3.48 |
| **537** | **SFRP2** | 6423 | <0.001 | 3.48 |
| **538** | **FOS** | 2353 | <0.001 | 3.5 |
| **539** | **SH3GL2** | 6456 | <0.001 | 3.51 |
| **540** | **MYOM1** | 8736 | <0.001 | 3.53 |
| **541** | **PTGDS** | 5730 | <0.001 | 3.53 |
| **542** | **HLA-DRA** | 3122 | <0.001 | 3.58 |
| **543** | **ALDH1A3** | 220 | <0.001 | 3.63 |
| **544** | **EMP3** | 2014 | <0.001 | 3.63 |
| **545** | **ADAMTS8** | 11095 | <0.001 | 3.67 |
| **546** | **LMOD1** | 25802 | <0.001 | 3.68 |
| **547** | **ADAMTS1** | 9510 | <0.001 | 3.69 |
| **548** | **TMOD1** | 7111 | <0.001 | 3.72 |
| **549** | **HLA-DQA1** | 3117 | <0.001 | 3.73 |
| **550** | **SEPP1** | 6414 | <0.001 | 3.73 |
| **551** | **ABCA8** | 10351 | <0.001 | 3.74 |
| **552** | **C1S** | 716 | <0.001 | 3.77 |
| **553** | **ANTXR2** | 118429 | <0.001 | 3.79 |
| **554** | **MYL9** | 10398 | <0.001 | 3.79 |
| **555** | **PDGFRA** | 5156 | <0.001 | 3.79 |
| **556** | **SDPR** | 8436 | <0.001 | 3.79 |
| **557** | **CYP1B1** | 1545 | <0.001 | 3.8 |
| **558** | **CCND2** | 894 | <0.001 | 3.81 |
| **559** | **FXYD6** | 53826 | <0.001 | 3.81 |
| **560** | **EGR2** | 1959 | <0.001 | 3.82 |
| **561** | **SERPINE2** | 5270 | <0.001 | 3.83 |
| **562** | **COL1A2** | 1278 | <0.001 | 3.88 |
| **563** | **DKFZP564O0823** | 25849 | <0.001 | 3.92 |
| **564** | **SGCE** | 8910 | <0.001 | 3.96 |
| **565** | **PTRF** | 284119 | <0.001 | 3.98 |
| **566** | **ATP1A2** | 477 | <0.001 | 3.99 |
| **567** | **RASL12** | 51285 | <0.001 | 3.99 |
| **568** | **PDK4** | 5166 | <0.001 | 4.02 |
| **569** | **IGFBP5** | 3488 | <0.001 | 4.03 |
| **570** | **TNC** | 3371 | <0.001 | 4.03 |
| **571** | **CDH11** | 1009 | <0.001 | 4.04 |
| **572** | **JAM3** | 83700 | <0.001 | 4.08 |
| **573** | **PMP22** | 5376 | <0.001 | 4.09 |
| **574** | **VIM** | 7431 | <0.001 | 4.1 |
| **575** | **COL6A1** | 1291 | <0.001 | 4.11 |
| **576** | **CYBRD1** | 79901 | <0.001 | 4.11 |
| **577** | **CASQ2** | 845 | <0.001 | 4.26 |
| **578** | **TPM1** | 7168 | <0.001 | 4.26 |
| **579** | **DPYSL3** | 1809 | <0.001 | 4.3 |
| **580** | **DUSP1** | 1843 | <0.001 | 4.3 |
| **581** | **P2RX1** | 5023 | <0.001 | 4.3 |
| **582** | **COX7A1** | 1346 | <0.001 | 4.31 |
| **583** | **PTGIS** | 5740 | <0.001 | 4.32 |
| **584** | **TPM2** | 7169 | <0.001 | 4.32 |
| **585** | **EGR1** | 1958 | <0.001 | 4.34 |
| **586** | **SORBS1** | 10580 | <0.001 | 4.34 |
| **587** | **MOXD1** | 26002 | <0.001 | 4.35 |
| **588** | **FOXF1** | 2294 | <0.001 | 4.39 |
| **589** | **FLJ21986** | 79974 | <0.001 | 4.47 |
| **590** | **BIN1** | 274 | <0.001 | 4.53 |
| **591** | **PLAC9** | 219348 | <0.001 | 4.82 |
| **592** | **AEBP1** | 165 | <0.001 | 4.91 |
| **593** | **RBPMS2** | 348093 | <0.001 | 4.93 |
| **594** | **RGS2** | 5997 | <0.001 | 4.97 |
| **595** | **KCNMB1** | 3779 | <0.001 | 5 |
| **596** | **ALDH1A1** | 216 | <0.001 | 5.06 |
| **597** | **FAM107A** | 11170 | <0.001 | 5.11 |
| **598** | **MYLK** | 4638 | <0.001 | 5.21 |
| **599** | **ACTA2** | 59 | <0.001 | 5.3 |
| **600** | **RGS1** | 5996 | <0.001 | 5.43 |
| **601** | **COL16A1** | 1307 | <0.001 | 5.46 |
| **602** | **FOSB** | 2354 | <0.001 | 5.5 |
| **603** | **SPON1** | 10418 | <0.001 | 5.56 |
| **604** | **TAGLN** | 6876 | <0.001 | 5.59 |
| **605** | **DMN** | 23336 | <0.001 | 5.65 |
| **606** | **CRYAB** | 1410 | <0.001 | 5.69 |
| **607** | **DCN** | 1634 | <0.001 | 5.69 |
| **608** | **MRGPRF** | 219928 | <0.001 | 5.73 |
| **609** | **SMOC2** | 64094 | <0.001 | 5.79 |
| **610** | **CTGF** | 1490 | <0.001 | 5.8 |
| **611** | **IGJ** | 3512 | <0.001 | 5.91 |
| **612** | **PTGS1** | 5742 | <0.001 | 5.95 |
| **613** | **SPARCL1** | 8404 | <0.001 | 6.14 |
| **614** | **FHL1** | 2273 | <0.001 | 6.26 |
| **615** | **KIAA0367** | 23273 | <0.001 | 6.35 |
| **616** | **LUM** | 4060 | <0.001 | 6.72 |
| **617** | **PGM5** | 5239 | <0.001 | 6.87 |
| **618** | **PCP4** | 5121 | <0.001 | 7.4 |
| **619** | **SRPX** | 8406 | <0.001 | 7.68 |
| **620** | **DES** | 1674 | <0.001 | 8.84 |
| **621** | **MFAP4** | 4239 | <0.001 | 9.97 |
| **622** | **CNN1** | 1264 | <0.001 | 10.95 |
| **623** | **MYH11** | 4629 | <0.001 | 12.53 |

* The parametic *P*-values were obtained by two sample t-tests.

^†^ Genes were sorted by fold change values.

Abbreviations: NMIBC, non-muscle invasive bladder cancer
